# Completion of the continuum of maternity care and associated factors among women who gave birth in the last 6 months in Chelia district, West Shoa zone, Ethiopia: A community-based cross-sectional study

**DOI:** 10.3389/fpubh.2022.1026236

**Published:** 2023-01-04

**Authors:** Temesgen Daksisa Buli, Negash Wakgari, Gemechu Ganfure, Fikadu Wondimu, Dereje Lemma Dube, Gonfa Moti, Yonas Sagni Doba

**Affiliations:** ^1^Chelia District Health Office, Oromia Regional Health Bureau, Addis Ababa, Ethiopia; ^2^Department of Midwifery, College of Medicine and Health Sciences, Ambo University, Ambo, Ethiopia; ^3^Department of Pediatrics and Child Health, College of Medicine and Health Sciences, Ambo University, Ambo, Ethiopia; ^4^Department of Obstetrics and Gynecology, Ambo University Referral Hospital, Ambo, Ethiopia; ^5^Department of Obstetrics and Gynecology, College of Medicine and Health Science, Ambo University, Ambo, Ethiopia; ^6^Department of Midwifery, College of Medicine and Health Sciences, Wachemo University, Hossana, Ethiopia

**Keywords:** maternity continuum of care, mothers, West Shoa zone, uptake of the continuum of maternity care, completion

## Abstract

**Background:**

The continuum of maternity care is a continuity of care that a woman receives during pregnancy, childbirth, and the postpartum period from skilled providers in a comprehensive and integrated manner. Despite existing evidence regarding maternal healthcare services discretely, the continuum of maternity care and its associated factors are not well-known in Ethiopia.

**Objective:**

This study assessed the completion of the maternity continuum of care and associated factors among women who gave birth 6 months prior to the study in the Chelia district.

**Methods:**

A community-based cross-sectional study with a stratified random sampling technique was conducted among 428 mothers at 10 randomly selected kebeles. Pretested and structured questionnaires were used to collect data. Bi-variable and multivariable logistic regression analyzes were performed to identify associated factors. Adjusted odds ratio with its 95% confidence interval was used to determine the degree of association, and statistical significance was declared at a *p*-value of <0.05.

**Results:**

In this study, 92 (21.5%) mothers completed the continuum of maternity care. Secondary and above education of mothers (AOR = 4.20, 95% CI:1.26–13.97), ≤30 min spent on walking by foot (AOR = 4.00, 95% CI: 1.67–9.58), using an ambulance to reach health facility (AOR = 3.68, 95% CI: 1.23–11.06), para ≥5 mothers (AOR = 0.21, 95% CI: 0.05–0.90), planned pregnancy (AOR = 3.29, 95% CI: 1.02–10.57), attending pregnant women's conference (AOR = 13.96, 95% CI: 6.22–31.30), early antenatal care booking (AOR = 3.30, 95% CI: 1.54–7.05), accompanied by partners (AOR = 3.64, 95% CI: 1.76–7.53), and informed to return for postnatal care (AOR = 3.57, 95% CI: 1.47–8.70) were the factors identified.

**Conclusion:**

In this study, completion of the maternity continuum of care was low. Therefore, appropriate strategic interventions that retain women in the continuum of maternity care by targeting those factors were recommended to increase the uptake of the continuum of maternity care.

## Introduction

Access and use of maternity care services during pregnancy, childbirth, and the postnatal period from skilled providers are essential for the survival and wellbeing of the mother and newborn (1). An uptake of maternity care services in a continuum of care approach has a fundamental role in the reduction of maternal and neonatal morbidity and mortality and saves mothers and babies. The key components of maternity care services are antenatal (ANC), skilled birth attendance (SBA), and postnatal care (PNC) (2).

The maternity continuum of care is the continuity of care received by women during pregnancy, childbirth, and the postpartum period in comprehensive and integrated ways from a skilled provider (1, 3, 4). It is the package of high-impact maternal and child survival interventions along the continuum of care (5) and a strategy designed to monitor the quality of care provided for women and newborns (6). It is a simple, cost-effective, and low-technology intervention approach that can significantly reduce most of the preventable maternal and neonatal mortalities and maximizes the potential of women and neonates to enjoy the highest achievable level of health (1, 7, 8). The continuum of maternity care gives emphasis on two key dimensions which are the time and place or level of care. The time dimension highlights the importance of linkages among the packages of maternity care service provision over the time during pregnancy, childbirth, and the postpartum period (9). The place or level of care dimension includes the home, primary, secondary, and tertiary levels of care in healthcare deliveries (9–11). Globally, in 2017, a total of 2,95,000 women died from pregnancy and childbirth-related complications; of which, the majority of the deaths (86%) were accounted by South Asia and sub-Saharan Africa. Similarly, sub-Saharan Africa alone accounted for roughly two-thirds (66%) of global maternal deaths (12). Ethiopia is one of the countries with a high alert maternal mortality ratio of 401 deaths per 1,00,000 live births and accounted for about 5% of global maternal deaths (10, 12). Most of these deaths occur during childbirth and the days before the end of the first week after childbirth (11), due to the leading causes such as hemorrhage, hypertensive disorders during pregnancy, and sepsis (13, 14), and more than 80% of them are preventable through appropriate maternity care services during pregnancy, childbirth, and the postpartum period in a continuum of care manner (6, 13, 15).

While substantial progress has been made, Ethiopia still needs to go about six and more than two-fold faster to achieve the 2030 global target of maternal and neonatal mortality reduction to less than 70 maternal deaths per 1,00,000 and less than 12 neonatal deaths per 1,000 live births, respectively (12, 16). Evidence from low- and middle-income countries (LMICs) showed that completion of the maternity continuum of care was reported ranging from 5 to 75% from the studies conducted in Cambodia and Nepal, respectively (17, 18). In Ethiopia, the completion of the maternity continuum of care was reported as ranging from 6.56 to 67.8% (19, 20). Despite this evidence, the continuum of maternity care among mothers within 6 months of delivery was not addressed (21–25).

Although a number of efforts were attempted so far to address the status of maternal healthcare service utilization discretely, there is a dearth of evidence regarding the prevalence and factors associated with the completion of the continuum of maternity care among mothers who gave birth 6 months prior to the study in Ethiopia in general and in Oromia regional state in specific including the study area. Therefore, this study aimed to assess the completion of the maternity continuum of care and associated factors among women who gave birth in the last 6 months in the Chelia district.

## Materials and methods

### Study design, setting, and population

A community-based cross-sectional study design was conducted in Chelia district, West Shoa zone, Oromia regional state, western Ethiopia, from 20 August to 30 September 2021. The district is located 178 km from Addis Ababa, the capital city of Ethiopia. The district has 20 administrative kebeles (the lowest administrative unit in Ethiopia). Regarding health facilities, there are 1 general hospital, 4 health centers, 18 health posts, and 11 different types of private health facilities in the district. All mothers who gave birth 6 months prior to the study were the source population, whereas all mothers who gave birth 6 months prior to the study in randomly selected kebeles were the study population. Moreover, all mothers who were booked for antenatal care in the last pregnancy and found between the first week and 6 months after delivery were included in the study. However, mothers who fulfilled inclusion criteria but came from other areas after receiving any one of the maternity care services and those mothers who were critically ill during the data collection period were excluded from the study.

### Sample size determination

The sample size for this study was calculated for both specific objectives and compared, and the maximum sample size was taken. First, a single population proportion formula was used by considering the following assumptions: the proportion (P) of maternity continuum of care completion of 21.6%, which was taken from the previous study (26), 95% confidence interval at the critical value of *Z*α/2 = 1.96, 5% of absolute precision, and a design effect of 1.5.

Therefore, with a 10% non-response rate, the minimum sample size for the first specific objective was 430. Similarly, the sample size was calculated for the determinant factors (19, 21, 27) to check for the adequacy of sample size by using 95% CI, power 80%, and ratio 1 (Table 1). By comparing the calculated sample size, 430 were taken as the final sample size for the study.

**Table 1:** Sample size determination for the second objective for mothers who gave birth in the last 6 months in Chelia district, West Shoa zone, Oromia region, Ethiopia, 2021.

**Factors**	**CI (%)**	**Power (1–β)**	**Ratio**	**DE**	**NRR**	**Proportion of outcome among exposed**	**Proportion of outcome among unexposed**	**OR**	**Final sample size (*n*)**
Being informed about pregnancy complications	95%	80%	1	1.5	10%	65.24%	34.76%	2.31	338
Timing of first ANC visit	95%	80%	1	1.5	10%	42.2%	15.3%	2.57	383
Gestational age during first ANC	95%	80%	1	1.5	10%	64.9%	15.8%	3.4	218

CI, confidence interval; DE, design effect; NRR, non-response rate; OR, odds ratio.

### Sampling procedure and techniques

A two-stage stratified random sampling technique was employed for the selection of study subjects. In the first stage, 10 kebeles (two from urban and eight from rural strata) were selected by simple random sampling technique from a total of 20 kebeles in the district. In the second stage, the lists of households with eligible mothers were prepared from family folders found at a health post in collaboration with Health Extension Workers (HEWs) and used as a sampling frame. Then, the sample size was proportionally allocated to each selected kebele according to their population size, and a simple random sampling technique was applied to select households where eligible mothers were available. Finally, selected mothers were interviewed by data collectors at their homes with the guidance of the women's development army (WDA) from their respective villages. Mothers who were absent on the first day were revisited for the second time and absentees after two repeated visits were replaced by an immediate neighbor who fulfills inclusion criteria (Table 2).

**Table 2:** Proportional allocation to population size to determine the sample size.

**SN**	**Kebele**	**Target**	**Proportion**	**Proportional**
		**population**		**allocation to**
				**population size**
1	Ale Hula Dhabi	138	0.12	50
2	Ale Soyema	60	0.05	22
3	Bilof Keku	113	0.10	41
4	Chobi Tulu Chori	82	0.07	30
5	Gedo-01	158	0.13	58
6	Gedo-02	167	0.14	61
7	Jarso Dire Geda	154	0.13	56
8	Tulu Kosoru	106	0.09	39
9	Tulu Mera	116	0.10	42
10	Tulu Nacha	82	0.07	30
	Total	1,176	1.00	430

### Data collection tools and procedures

A structured interviewer-administered questionnaire was adapted from different literature (19, 21–23, 26–31). The tool was arranged into three sub-titles namely socio-demographic and socioeconomic characteristics, reproductive and obstetric-related questions, and maternal healthcare service-related questions. It was initially developed in English and translated to Afan Oromo and then translated back to English by different language experts to ensure its consistency and accuracy. Data collection was conducted by four trained BSc Nurses who were recruited from outside of their catchment area. The data collection process was supervised by two health officers and a principal investigator during the data collection period.

### Data quality management

Before data collection, 1-day training was given to data collectors and supervisors regarding the objective of the study, data collection procedures, and ethical considerations during data collection. Preceding the data collection, the tool was pretested on 5% of the calculated sample size. After pretesting, necessary modifications were considered. A clear explanation of the purpose and objective of the study was provided for all respondents before beginning an interview. Close supervision was carried out by supervisors and the principal investigator during the time of data collection. All filled questionnaires were checked for completeness and accuracy before data entry.

### Data processing and analysis

Data were checked for completeness, coded, and entered into the computer using Epi-Data version 3.1 and exported to SPSS version 25 for analysis. Descriptive statistics and binary logistic regression (Bi-variable and multivariable logistic regression analyzes) model was done. Model fitness and multicollinearity effects between covariates were checked. From the bi-variable logistic regression analysis, variables having a *p*-value of ≤0.25 were considered eligible for multivariable logistic regression analysis. Finally, adjusted odds ratios (AOR) with their 95% confidence intervals were estimated to identify the presence and strength of associations, and statistical significance was declared at a *p*-value of <0.05.

### Operational definition

- The maternity continuum of care was considered complete and coded as “1” when a mother received the three maternal healthcare services along with the continuum of care pathway from a skilled provider. These include four or more antenatal care (ANC) visits, childbirth attended by a skilled birth attendant (SBA), and postnatal checkup within the first week after delivery at health facilities [excluding pre-discharge postnatal care] or home by a skilled provider in a continuum of the care pathway and otherwise considered as not complete the continuum of care and coded as “0” (15, 21, 24–26, 30).
- A skilled provider is a professionally trained health worker which includes a medical doctor, midwife, nurse, and health officer (32).

### Ethics statement

An ethical clearance letter with a Ref. No. of PGC/167/2021 was obtained from the Institutional Review Board of Ambo University, College of Medicine and Health Sciences. Permission and a support letter to conduct the study were taken from the Chelia district health office. The purpose and importance of the study were explained, and finally, informed written consent was taken from all respondents. In addition, the confidentiality and privacy of the study participants were assured and respected.

## Results

### Socio-demographic and economic characteristics

A total of 428 respondents yielding a response rate of 99.5% participated in the study. The mean age of the respondents was 28.9 (SD ± 4.871) years. One hundred and fifty-seven (36.7%) of the mothers attained a secondary and above level of education, whereas 103 (24.1%) of them have no formal education. More than half, 249 (58.2%), of the respondents spent more than 30 min walking on foot to reach the nearest health facility and 277 (73.6%) of them got the facility by walking on their feet for seeking maternity care services (Table 3).

**Table 3:** Socio-demographic and economic characteristics of mothers who gave birth in the last 6 months in Chelia district, West Shoa zone, Oromia region, Ethiopia, 2021 (*N* = 428).

**Variables**	**Category**	**Frequency**	**Percent**
Age of the respondents	< 20 years	5	1.2
	20–34 years	348	81.3
	≥35 years	75	17.5
Residence	Urban	119	27.8
	Rural	309	72.2
Educational status	No formal education	103	24.1
	Primary (1–8)	168	39.2
	Secondary and above	157	36.7
Religion	Orthodox	140	32.7
	Protestant	277	64.7
	Others*	11	2.6
Ethnicity	Oromo	420	98.1
	Others**	8	1.9
Occupation	Unemployed	387	90.4
	Employed	41	9.6
Marital status	Married	413	96.5
	Others***	15	3.5
Husband's educational status (*N* = 413)	Unable to read and write	71	17.2
	Primary (1–8)	178	43.1
	Secondary (9–12) and above	164	39.7
Husband's occupation (*N* = 413)	Unemployed	337	81.6
	Employed	76	18.4
Decision maker regarding maternal health service	Husband only	28	6.5
	Women alone	23	5.4
	Both husband and women	365	85.3
	Family members	12	2.8
Time taken to reach the nearest health center/hospital	≤ 30 min	179	41.8
	>30 min	249	58.2
Mode of transportation used to reach health facility	Walking on foot	315	73.6
	Public transport	45	10.5
	Ambulance	68	15.9
Household's average monthly income in birr	≤ 1,000 ETB	93	21.7
	1,001–2,000 ETB	121	28.3
	>2,000 ETB	214	50.0

Others:

* Muslim, Wakefata.

** Amhara, Guragie, Tigrie.

*** Single, divorced, widowed.

### Reproductive and obstetrics-related characteristics

All respondents who participated in the study believed that all women need to have maternity care services from skilled providers and birth preparedness and complication readiness plans. Three hundred and thirty-four (78%) and 252 (58.9%) mothers know an appropriate time to start the first antenatal care visit and the need to have four and more ANC visits, respectively. Two hundred and eighty-seven (67.1%) mothers mentioned three and more practices of birth preparedness and complication plans. However, nearly, three-fourth, 307 (71.7%), of them reported that they need to be confined at home for the first 6 weeks after childbirth (Table 4).

**Table 4:** Knowledge of the respondents regarding reproductive and obstetric-related services of mothers who gave birth in the last 6 months in Chelia district, West Shoa zone, Oromia region, Ethiopia, 2021 (*N* = 428).

**Variables**	**Category**	**Frequency**	**Percent**
Appropriate time to begin ANC visits	Within the first trimester	334	78.0
	After the first trimester	94	22.0
Number of visits required for ANC	Only once	6	1.4
	Two times	27	6.3
	Three times	143	33.4
	Four and above	252	58.9
Types of birth preparedness and complication readiness plan mentioned	Place of birth	343	80.1
	Supplies needed during childbirth	393	91.8
	Emergency transport	152	35.5
	Money	236	55.1
	People to support	257	60.0
	Potential for blood donor	6	1.4
Knowledge toward birth preparedness and complication readiness plan	Adequate	287	67.1
	Inadequate	141	32.9
Possible to leave home before 6 weeks for postnatal care services	Yes	121	28.3
	No	307	71.7
Home confinement days (*N* = 307)	≤ 42 days	122	39.7
	>42 days	185	60.3

More than three-fourth, 330 (77.1%), of the respondents got their first pregnancy at an age of 20 and more years. Sixty-one (14.3%) respondents were para five and more, and 52 (14.5%) mothers had experienced different types of obstetric complications during their previous reproductive lives. Among the respondents who participated in the study, 92 (21.5%) of them reported that the last pregnancy was unplanned and 116 (27.1%) respondents attended at least one session of a pregnant women's conference (Table 5).

**Table 5:** Reproductive and obstetric characteristics of mothers who gave birth in the last 6 months in Chelia district, West Shoa zone, Oromia region, Ethiopia, 2021 (*N* = 428).

**Variables**	**Category**	**Frequency**	**Percent**
Age at first pregnancy	< 20 years	98	22.9
	≥20 years	330	77.1
Number of children	1–2	182	42.5
	3–4	185	43.2
	≥5	61	14.3
Birth interval between successive birth (*N* = 358)	≤ 36 months	173	48.3
	>36 months	185	51.7
Ever experienced obstetric complication during their previous reproductive life (*N* = 358)	Yes	52	14.5
	No	306	85.5
History of previous contraceptive utilization	Yes	370	86.4
	No	58	13.6
Plan of the last pregnancy	Planned	336	78.5
	Unplanned	92	21.5
Attend pregnant women's conference	Yes	116	27.1
	No	312	72.9
Number of sessions attended (*N* = 116)	Only once	16	13.8
	Two times	34	29.3
	Three times	31	26.7
	Four times	35	30.2

### Maternal healthcare service utilization

#### Antenatal care service utilization

Of the total of 428 respondents, 148 (34.6%) of them initiated the first ANC visit early in the first trimester (within 12 weeks of gestation) and 183 (42.8%) of them attended ANC-4+ visits. About three-quarters, 318 (74.3%) and 346 (80.8%), of the respondents were informed about pregnancy-related complications and birth preparedness and complication readiness plans, respectively. Only, 141 (32.9%) of the respondents were accompanied by their partners to the maternity care service center (Table 6).

**Table 6:** Antenatal care service-related characteristics of mothers who gave birth in the last 6 months in Chelia district, West Shoa zone, Oromia region, Ethiopia, 2021 (*N* = 428).

**Variables**	**Category**	**Frequency**	**Percent**
Place at antenatal care visits made	Hospital	68	15.9
	Health center	278	65.0
	Health post	82	19.1
First time antenatal care booking	Early (≤ 12 weeks)	148	34.6
	Late (>12 weeks)	280	65.4
Number of antenatal care visits attended	Once	19	4.40
	Twice	62	14.5
	Three times	164	38.3
	Four and above	183	42.8
Told about pregnancy related complications	Yes	346	80.8
	No	82	19.2
Complications told about (*N* = 346)	Vaginal bleeding	329	95.1
	Vaginal gush of fluid	191	55.2
	Severe headache	276	79.8
	Blurred vision	202	58.4
	Fever	130	37.6
	Abdominal pain	64	18.5
Told about birth preparedness and complication readiness plan	Yes	318	74.3
	No	110	25.7
Birth preparedness and complication readiness plan ever told (*N* = 318)	Place of birth	301	94.7
	Supplies needed	274	86.2
	Emergency transport	123	38.7
	Emergency fund (Money)	173	54.4
	People to support	174	54.7
	Potential blood donor	2	0.60
Received more than one types of antenatal care services	Yes	322	75.2
	No	106	24.8
Routine examinations and tests	BP measured	358	83.6
	Urine test	332	77.6
	Blood sample taken	336	78.5
	HIV test	345	80.6
	Weight measured	368	86.0
	Nutrition counseling	343	80.1
Partner accompany during antenatal care visits	Yes	141	32.9
	No	287	67.1
Partner entered the room with the mother (*N* = 141)	Yes	41	29.1
	No	100	70.9

#### Labor and delivery service utilization

Among the total 428 respondents, 352 (82.2%) of them had childbirths attended by skilled birth attendants. Among mothers who received skilled birth attendance, only 50 (14.2%) of them stayed for 24 h and more in the health facility after giving birth. Two hundred and seventy-three (77.6%) respondents were informed about postpartum danger signs of both mothers and newborns, whereas 237 (67.3%) mothers were informed about when to return to health facilities for postnatal checkups (Table 7).

**Table 7:** Labor and delivery service-related characteristics of mothers who gave birth in the last 6 months in Chelia district, West Shoa zone, Oromia region, Ethiopia, 2021 (*N* = 428).

**Variables**	**Category**	**Frequency**	**Percent**
Place of childbirth	Hospital	130	30.4
	Health center	217	50.7
	Private clinic	5	1.2
	On the way to health facility	14	3.3
	Home	62	14.5
Mode of delivery	Spontaneous vaginal delivery	391	91.4
	Instrument assisted	15	3.5
	Cesarean section	22	5.1
Had skilled birth attendance	No	76	17.8
	Yes	352	82.2
Informed about postpartum danger signs before discharge (*N* = 352)	No	79	22.4
	Yes	273	77.6
Maternal danger signs ever informed (*N* = 273)	Vaginal bleeding	265	97.1
	Fever	205	75.1
	Smelly vaginal bleeding	116	42.5
Newborn danger sign ever informed (*N* = 273)	Feeding less	259	92.8
	Too cold or too hot	48	17.6
	Convulsion	81	29.7
	Fast breathing	186	68.1
	Umbilicus red/pus	220	80.6
	Fever	101	37.0
	Others*	18	6.6
Duration of stay in health facility after delivery (352)	< 24 h	302	85.8
	≥24 h	50	14.2
Informed when to return for postnatal care before discharge (*N* = 352)	Yes	237	67.3
	No	115	32.7
In position to come back in the future for the same services to the same provider	Yes	342	97.2
	No	10	2.8

* Too sleepy, pus from eye.

#### Postnatal care service utilization

Of the total of 428 respondents, nearly half, 213 (49.8), of them had at least one postnatal checkup in their last childbirth. However, only 139 (32.5%) mothers had at least one postnatal checkup within the first week of delivery. Most of the respondents were counseled for breastfeeding, 196 (92%), and postpartum family planning, 196 (92%). Among mothers who had postpartum checkups, 139 (65.3%) and 103 (48.4%) of their newborns got weight measurement and immunization services, respectively (Table 8).

**Table 8:** Postnatal care service-related characteristics among mothers who gave birth in the last 6 months in Chelia district, West Shoa zone, Oromia region, Ethiopia, 2021 (*N* = 213).

**Variables**	**Category**	**Frequency**	**Percent**
Have PNC check up	Yes	213	49.8
	No	215	50.2
Time when postnatal care received	Within 48 h	87	40.8
	3–7 days	52	24.4
	7–14 days	24	11.3
	2–6 weeks	50	23.5
Place of postnatal care services	Hospital	46	21.6
	Health center	108	50.7
	Health post	18	8.4
	Private clinic	4	1.9
	Home	37	17.4
Reason for having postnatal care	For checkup	86	40.4
	I was sick	63	29.6
	Baby was sick	37	17.4
	Visited at home by HEWs	27	12.7
Postpartum care services provided for mothers	BP measured	105	49.3
	Vaginal bleeding checked	94	44.1
	Temperature measured	64	30
	Counseled for breast feeding	196	92
	Counseled on nutrition	155	72.8
	Informed about postpartum danger sign	97	45.5
	Counseled for postpartum family planning	196	92
	Provided postpartum family planning	55	25.8
Postnatal care services provided for newborns	Temperature measured	94	44.1
	Weight measured	139	65.3
	Observed for breast feeding	172	80.8
	Encouraged for exclusive breast feeding	198	93
	Immunized	103	48.4
	Advised to exposure to sun light	34	16
	Informed about danger sign	55	25.8

**Figure 1:**
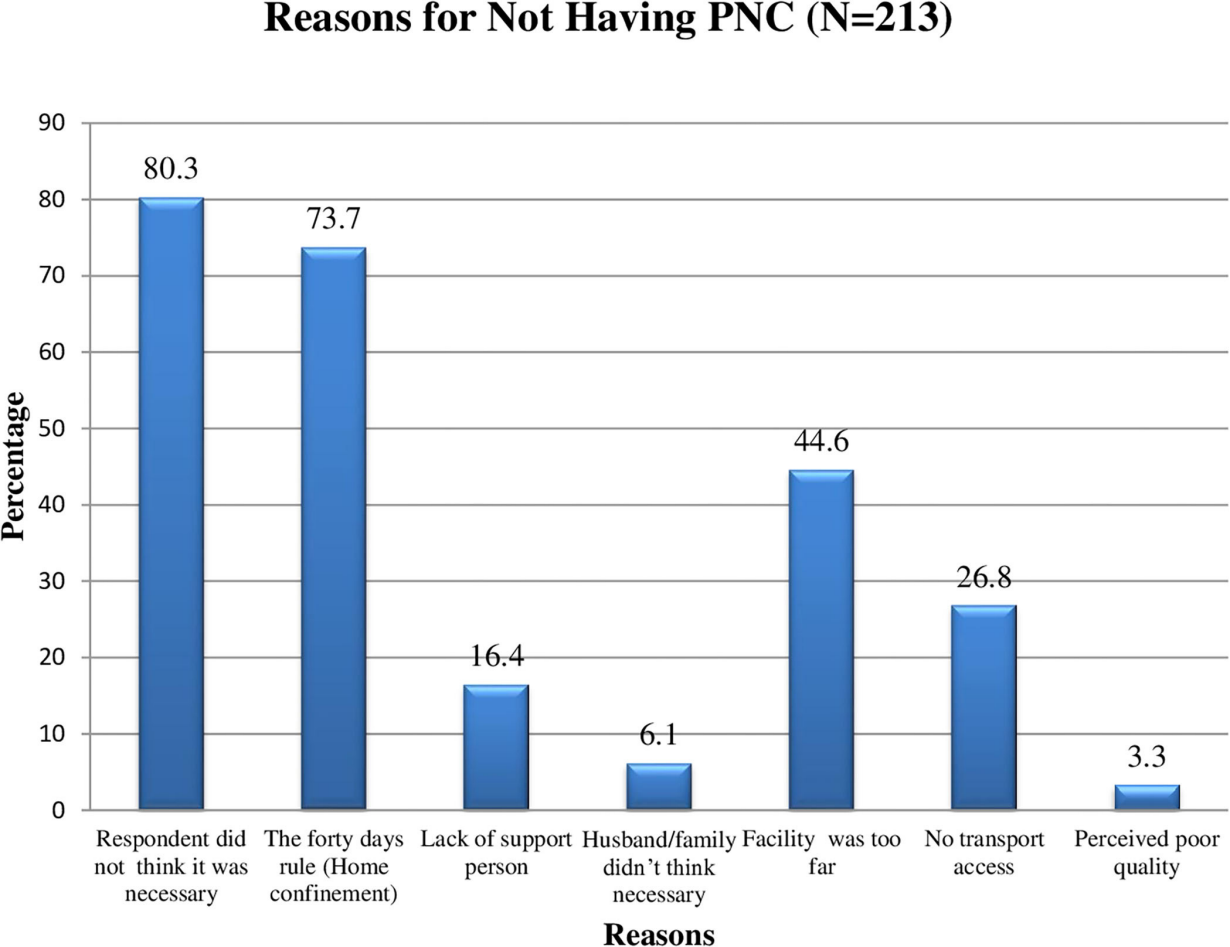
Reasons for not having postpartum check-up among mothers who gave birth in the last 6 months in Chelia district, West Shoa zone, Oromia, Ethiopia, 2021.

The majority of the respondents reported that they failed to have postnatal checkups due to being unaware of the need to have postnatal checkups, 171 (80.3%), and the 40-day rule of home confinement in their communities, 157 (73.7%). Moreover, 95 (44.6%) and 57 (26.8%) respondents claimed that the facility was too far and no access to transport to have a postnatal visit, respectively (Figure 1).

### Completion of maternity continuum of care

Among all study participants, 183 (42.8%) had attended ANC-4+ visits and 167 (39%) of them retained in the continuum of care and received childbirth services from skilled birth attendants. However, only 92 (21.5%) mothers retained in the continuum of the care pathway and received at least one postnatal care service within the first week of delivery. Therefore, in this study, the overall completion of the maternity continuum of care was 21.5% (95% CI: 17.8–25.5).

**Figure 2:**
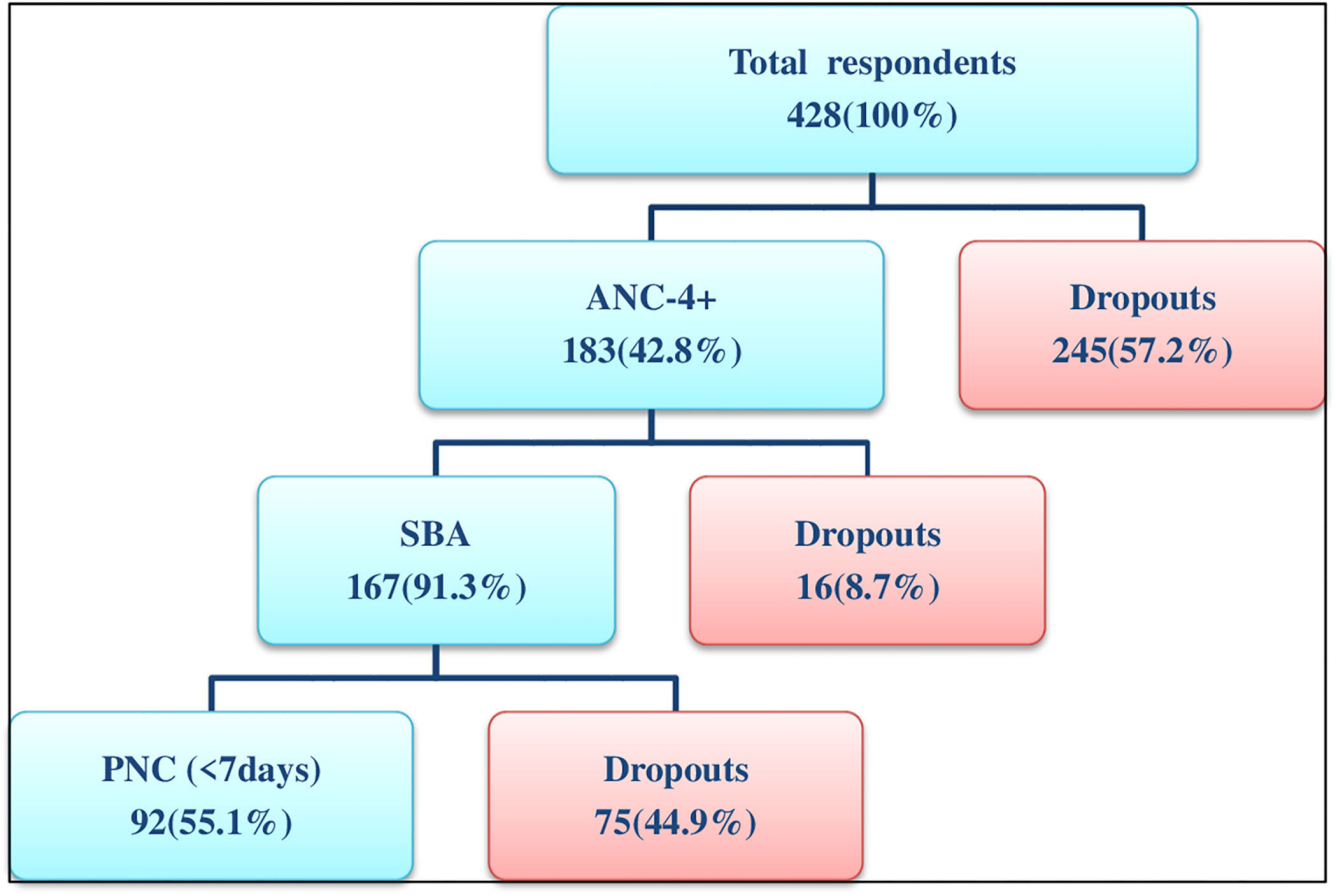
Flowchart of the continuum of maternity care services utilization among mothers who gave birth in the last 6 months in Chelia district, West Shoa zone, Oromia, Ethiopia, 2021.

Among the total 428 mothers who participated in the study, 336 (78.5%) of them were dropouts from the completion of the maternity continuum of care. Higher proportions of dropouts from the continuum of maternity care service utilization were observed at the completion of focused antenatal care visits and postnatal care service utilization (Figure 2).

### Factors associated with the completion of the maternity continuum of care

Place of residence, mother's and husband's level of education and employment status, time spent walking by foot to reach the nearest health facility, means of transportation used to reach a health facility, household's average monthly income, belief in a postpartum period home confinement, knowledge of appropriate time for first ANC booking, maternal age at first pregnancy, number of children, planned pregnancy, attendance of pregnant women's conference, place of ANC visits, early ANC booking within the first trimester, informed about pregnancy-related complications, and birth preparedness and complication readiness plan, receiving adequate ANC services, being accompanied by partners, being informed about postpartum danger signs, and when to return for a postpartum checkup before discharge were identified as candidate variables for multivariable logistic regression analysis.

In multivariable logistic regression analysis, mothers' secondary and above level of education, time spent ≤30 min by walking on foot to reach a health facility, use of an ambulance as a means of transportation, being para 5 and more, having planned pregnancy, attendance of pregnant women's conference (PWC), early ANC booking within the first trimester, being accompanied by partners, and informed when to return for postnatal checkups before discharge were variables associated with the completion of maternity continuum of care at a *p*-value of <0.05.

Mothers who attained a secondary and above level of education were 4.20 times more likely to complete the maternity continuum of care compared to those mothers who have no formal education (AOR = 4.20, 95% CI: 1.26–13.97). Mothers who spent ≤30 min of walking to reach the nearest health facility were four times more likely to complete the maternity continuum of care compared to mothers expected to spend more than 30 min of walking to reach the nearest health facility (AOR = 4.00, 95% CI: 1.67–9.58). In addition, mothers who used an ambulance as a means of transportation to reach the health facility were 3.68 times more likely to complete the maternity continuum of care than those who did not use any of the vehicles but by walking on their feet (AOR = 3.68, 95% CI: 1.23–11.06).

This study also identified that para five and more mothers were 79% less likely to complete the maternity continuum of care compared to those mothers who have 1–2 children (AOR = 0.21, 95% CI: 0.05–0.90). In this study, mothers to whom the last pregnancy was planned were 3.29 times more likely to complete the maternity continuum of care than their counterparts (AOR = 3.29, 95% CI: 1.02–10.57). Mothers who attended pregnant women's conferences were 13.96 times more likely to complete the maternity continuum of care than those who did not attend any session (AOR = 13.96, 95% CI: 6.22–31.30). The odds of completing the maternity continuum of care among the mothers who started ANC visits in the first trimester were 3.30 times higher than their counterparts (AOR = 3.30, 95% CI: 1.54–7.05).

Moreover, mothers who were accompanied by their partners to health facilities during their antenatal care visits were 3.64 times more likely to complete the maternity continuum of care compared to their counterparts (AOR = 3.64, 95% CI: 1.76–7.53). Similarly, mothers who were informed when to return for postnatal care during childbirth services to a health facility had 3.57 times higher odds of completing the maternity continuum of care than those never informed about the need to return to postnatal checkup while discharged after delivery (AOR = 3.57, 95% CI: 1.47–8.70) (Table 9).

**Table 9:** Factors associated with the completion of maternity continuum of care among mothers who gave birth in last 6 months in Chelia district, West Shoa zone, Oromia, Ethiopia, 2021.

**Variables**	**Category**	**Complete maternity**		**COR (95%CI)**	**AOR (95%CI)**	***P*-value**
		**continuum of care**	
		**Yes**	**No**			
Residence	Urban	31	88	1.43 (0.87–2.35)	0.54 (0.2–1.47)	0.230
	Rural	61	248	1	1	
Educational status of women	No formal education	8	96	1	1	
	Primary (1–8)	34	137	2.98 (1.32–6.72)	1.79 (0.0.54–5.93)	0.339
	Secondary & above	50	103	5.83 (2.63–12.92)	4.20 (1.26–13.97)*	**0.020**
Occupation of women	Employed	18	23	3.31 (1.70–6.45)	0.73 (0.17–3.09)	0.664
	Unemployed	74	313	1	1	
Husband's educational status	Never attend school	7	64	1	1	
	Primary (1–8)	31	150	1.89 (0.79–4.51)	2.12 (0.47–9.61)	0.332
	Secondary and above	52	109	4.36 (11.87–10.18)	3.41 (0.0.75–15.58)	0.120
Husband's occupation	Unemployed	57	280	1	1	
	Employed	33	43	3.77 (2.21–6.44)	1.27 (0.41–3.99)	0.676
Traveling time to reach health facility on foot	≤ 30 min	63	116	4.12 (2.51–6.75)	4.00 (1.67–9.58)*	**0.002**
	>30 min	29	220	1	1	
Mode of transportation used	Walking on foot	54	261	1	1	
	Public transport	8	37	1.05 (0.46–2.37)	0.93 (0.31–2.85)	0.905
	Ambulance	30	38	3.82 (2.18–6.69)	3.68 (1.23–11.06)*	**0.020**
Household's average monthly income in birr	≤ 1,000	11	82	1	1	
	1,001–2,000	20	101	1.48 (0.67–3.26)	1.26 (0.35–4.50)	0.720
	≥2,001	61	153	2.97 (1.48–5.96)	2.31 (0.76–7.06)	0.142
Think home confinement till 6 weeks after delivery	Yes	48	73	3.93 (2.42–6.38)	1.19 (0.49–2.91)	0.699
	No	44	263	1	1	
Know when to start the first antenatal care visit	In the first trimester	86	248	5.09 (2.15–12.05)	1.79 (0.48–6.64)	0.383
	After first trimester	6	88	1	1	
Knowledge about birth preparedness and complication readiness plan	Adequate	70	217	1.75 (1.03–2.96)	0.68 (0.27–1.74)	0.422
	Inadequate	22	119	1	1	
Age at first pregnancy	< 20 years	6	42	1	1	
	≥20 years	86	294	2.05 (0.84–4.98)	1.30 (0.48–3.54)	0.605
Number of childbirth	1–2	51	131	1	1	
	3–4	34	151	0.58 (0.35–0.95)	0.57 (0.26–1.25)	0.161
	≥5	7	54	0.33 (0.14–0.78)	0.21 (0.05–0.90)*	**0.036**
Plan of the last pregnancy	Planned	84	252	3.50 (1.63–7.53)	3.29 (1.02–10.57)*	**0.046**
	Unplanned	8	84	1	1	
Attended pregnant women's conference	Yes	66	50	14.52 (8.43–25.02)	13.96 (6.22–31.30)**	**0.001**
	No	26	286	1	1	
Place at antenatal care visits made	Hospital	20	48	8.13 (2.62–25.21)	4.03 (0.48–34.25)	0.202
	Health center	68	210	6.31 (2.23–17.89)	3.58 (0.52–24.78)	0.197
	Health post	4	78	1	1	
First time antenatal care booking	Early (≤ 12 weeks)	65	83	7.34 (4.40–12.25)	3.30 (1.54–7.05)*	**0.002**
	Late (>12 weeks)	27	253	1	1	
Told about pregnancy related complications	Yes	87	259	5.17 (2.03–13.20)	1.16 (0.25–5.46)	0.859
	No	5	77	1	1	
Told about birth preparedness and complication readiness plan	Yes	87	231	7.91 (3.12–20.05)	2.57 (0.77–8.52)	0.124
	No	5	105	1	1	
Received adequate antenatal care services	Yes	84	238	4.32 (2.02–9.27)	0.95 (0.29–3.17)	0.934
	No	8	98	1	1	
Partner accompany during antenatal care visits	Yes	64	77	7.69 (4.61–12.83)	3.64 (1.76–7.53)**	**0.001**
	No	28	259	1	1	
Informed about postpartum danger signs before discharge	Yes	84	189	3.94 (1.82–8.56)	1.33 (0.44–4.05)	0.621
	No	8	71	1	1	
Informed when to return for postnatal care before discharge	Yes	80	157	4.37 (2.27–8.43)	3.57 (1.47–8.70)*	**0.005**
	No	12	103	1	1	

* Statistically significant at *p* < 0.05.

** Statistically significant at *p* < 0.001. The bold values indicate the statistically significant variables multi variable logistic regression analysis at *p*-value less than 0.05.

## Discussion

In this study, the overall completion of the maternity continuum of care was found to be 21.5% (95% CI: 17.8–25.5). Mothers' educational status, time spent by walking on foot and means of transportation used to reach the nearest health facility, parity, having planned pregnancy, attending pregnant women's conference, time of antenatal care booking, partners accompany, and informing when to return for postnatal care before discharge were factors associated with the completion of maternity continuum of care.

The completion of the maternity continuum of care in the present study is 21.5% (95% CI: 17.8–25.5). This finding revealed that a significant number of women dropped out from the continuum of maternity care. A higher proportion of women dropped out from the completion of the continuum of the maternity care pathway. This finding is in line with a study conducted in Nigeria (18.5%) (33), India (19%) (34), and the Dabat and Gondar Zuria districts of the Northern Gondar zone (21.6%) (26). However, it is higher than studies conducted in Cambodia (5%) (18), Ghana (7.9%) (35), Tanzania (10%) (25), Ethiopia (6.56% and 9.1%) (19, 28), Arba Minch Zuria district (9.7%) (30), Legambo district of South Wollo zone (11.2%) (36), and West Gojjam zone (12.1%) (29). The possible reason might be attributed to the time difference between the studies, and there could be an improvement in the accessibility of health services, which in turn improves the utilization of maternity care services.

However, the current finding is lower than the studies conducted in Zambia (38%) (37), Egypt (50.4%) (24), Ghana (66%) (38), Cambodia (60%) (3), Nepal (41%, 75%) (17, 39), Debre Birhan town (37.2%) (21), Enemay district (45%) (31), Motta town and Hulet Eji Enese district (47%) (27), and Debre Markos town (67.8%) (20). The possible explanation for the discrepancy might be due to the difference in maternity care services provision attributed to the difference in socio-demographic status, socioeconomic status, geographical barriers, availability, and accessibility of services and infrastructures between the countries and regions. The other reason might be the difference in the study area in which mothers from the current study were mostly rural dwellers, less educated, and had inadequate knowledge of the importance of having maternity care services in a continuum of care pathway. Moreover, the previous studies reported as maternity continuum of care was completed when mothers attended at least one ANC visit, had childbirth attended by SBA, and had at least one postpartum checkup within 6 weeks of delivery, and/or mothers attended ANC-4+ visits, had childbirth attended by SBA, and had one health checkup within 48 h or 6 weeks postpartum period (19–21, 26–31, 36). As a result, the estimation of an outcome variable might be increased when compared with this study which considered maternity continuum of care was completed for mothers who attended ANC-4+ visits, had childbirth attended by SBA, and had at least one health checkup either for themselves or for their newborns within the first week of the postpartum period.

Evidence revealed that the educational attainment of mothers was found to have a significant association with the completion of the maternity continuum of care. Accordingly, this study revealed that those mothers who attained a secondary and above level of education were 4.20 times more likely to complete the maternity continuum of care compared to those mothers who did not attend school. This finding is consistent with the studies conducted in South Asia and sub-Saharan Africa (8), India (34), Pakistan (15, 40), Lao Peoples' Democratic Republic (41), Ghana (35, 42), Nigeria (33), Egypt (24), and Ethiopia (21, 27, 31), in which mothers who attained a secondary and above level of education had higher odds of completing maternity continuum of care than those mothers who never attended school. This could be explained by the fact that more educated women have better opportunities to be aware of and able to decide when and from where to receive the recommended maternal healthcare services to be utilized throughout their reproductive lives.

Another evidence showed that the time spent walking to reach the nearest health facility for maternal and neonatal healthcare services was found to have a significant association with the completion of the maternity continuum of care. Accordingly, the finding of this study revealed that mothers who traveled for ≤30 min on foot to arrive at the nearest health facility were 4.0 times more likely to complete the maternity continuum of care compared to those who expected to travel for more than 30 min. This finding is comparable with the studies conducted in Cambodia (18) and Motta town and Hulet Eji Enese district of Northeast Ethiopia (27). The possible explanation is due to the fact that access to health facilities will reduce the traveling time and costs for transportation to reach health facilities and then leads to better utilization of maternity continuum of care services.

In this study, mothers who used an ambulance as a means of transportation to reach the nearest health facility had 3.68 times more likely to complete the maternity continuum of care than those who got a health facility by walking on their feet. This finding is supported by studies conducted in Ghana and Dabat and Gondar Zuria districts of Northern Gondar zone, Ethiopia, in which mothers who used a car as a means of transportation to get health facilities for skilled delivery services were more likely to complete the maternity continuum of care than those who got on their foot (26, 42). This might be explained by the fact that using any type of vehicle to get health facility will reduce the time spent on traveling to reach the nearest health facility and enhances skilled delivery service which is a key component of the maternity continuum of care services. Thus, using the ambulance to get to a health facility has a positive association with the completion of the maternity continuum of care.

In this study, parity was found to be significantly associated with the completion of the maternity continuum of care. The finding of this study revealed that mothers with para five and more were 79% less likely to complete the maternity continuum of care compared to para 1–2 mothers. This finding is consistent with the studies conducted in Pakistan and Egypt in which mothers with one or two birth orders had higher odds of completing the maternity continuum of care compared to their counterparts (15, 24). The possible explanation might be due to the fact that pregnancy and having a child is a source of joy and a means to replace offspring across the global community and, thus, newly married couples and those mothers with low birth orders are motivated to seek and utilize the recommended maternity care services from skilled care providers.

Mothers having a planned pregnancy were 3.29 times more likely to complete the maternity continuum of care compared to their counterparts. This finding is consistent with the studies conducted in the Arba Minch Zuria district of Gamo zone and Enemay districts of Northwest Ethiopia in which mothers to whom the last pregnancy was planned had higher odds of completing the maternity continuum of care than their counterparts (30, 31). The reason might be due to the fact that women with planned and wanted pregnancies are psychologically well prepared and cautious about their pregnancy and thus interested in seeking and utilizing the recommended maternal and neonatal healthcare services compared to their counterparts.

The finding of this study showed that women who attended pregnant women's conferences had 13.96 times higher odds of completing the maternity continuum of care compared to their counterparts. This finding is consistent with the study conducted in the rural Libokemkem district of Northwest Ethiopia in which women who attended pregnant women's conferences had higher odds of utilizing maternal health services compared to their counterparts (43). The possible explanation might be due to the fact that women who attended a PWC during their pregnancy could be able to have adequate maternal and neonatal healthcare service information that was recommended for all pregnant women during pregnancy, childbirth, and the postpartum period from skilled providers and its merits and demerits of complete using and failing to do so compared with their counterparts.

This study also revealed women who were booked for ANC visits within the first 12 weeks of gestation for the last pregnancy were 3.30 times more likely to complete the maternity continuum of care than those mothers who were booked later on. This finding is supported by the studies conducted in Lao PDR (41), Tanzania (44), Motta town, and Hulet Eji Enese district of East Gojjam zone (27), Arba Minch Zuria district of Gamo zone (30), West Gojjam zone (29), and Debre Birhan town (21) in which women who were booked for ANC within the first trimester had a higher odds of completing maternity continuum of care than those booked late. The possible explanation might be due to the fact that women who booked the first ANC visit at the earlier gestational age would obtain greater opportunities of getting contact with skilled providers and adequate time for providers to communicate and enable the women to make adequate preparations and readiness to utilize the key maternity care components like skilled birth attendance and postpartum care.

The finding of this study also revealed that women who were accompanied by their partners while receiving maternity care services had 3.64 times more likely to complete the maternity continuum of care compared to those mothers who did not accompany skilled maternity care services by their partner. This finding is supported by the studies conducted in Bangladesh (45), Afghanistan (46), and Kenya (47) in which women who were accompanied by their partners to receive maternal and neonatal health services from skilled providers had higher odds of utilizing the next level of key maternity continuum of care components compared to their counterparts. The possible reason might be due to the synergetic effects of maternal healthcare service information and counseling provided to the couples could result in good awareness of the utilization of the key components of the maternity continuum of care services.

Mothers who were informed when to return to health facilities or maternity care services centers before discharge were 3.57 times more likely to complete the maternity continuum of care compared to their counterparts. This is consistent with the analysis done from EDHS 2016 data and a study conducted in the Dabat and Gondar Zuria district of Northern Gondar zone in which women who were informed about pregnancy-related complications and got appropriate health education regarding maternity care services to be utilized during their pregnancy, childbirth, and postpartum period had higher odds of completing maternity continuum of care than their counterparts (26, 28). The possible reason might be the health information and education regarding maternity care services and postpartum period danger signs provided to women during their pregnancy, childbirth, and postpartum period enhance their awareness and increase the need for seeking maternity care components compared to their counterparts. Generally, this study assessed the prevalence of the completion of the maternity continuum of care and identified factors associated with it among postpartum period mothers. This evidence might help program managers and healthcare providers to focus on identified factors to increase the uptake of the maternity continuum of care and, thus, contribute to the achievement of the global and national maternal mortality reduction plan.

This study has some limitations: First, it might be subjected to recall bias because the mothers may not recall all the services and advice they received. Second, since the study was cross-sectional, the temporal relationship between factors and outcome variables cannot be established. Finally, the incompleteness of the birth record at the health post by the health extension workers might lead the researchers to miss mothers during the sampling frame preparation.

## Conclusion

An overall completion of the continuum of maternity care was low in the study area. Educational status of mothers, time spent by walking on foot and means of transportation used to reach the nearest health facility, parity, having planned pregnancy, attending pregnant women's conference, time of antenatal care booking, partners accompany, and informing when to return for postnatal care before discharge were factors associated with the completion of maternity continuum of care. Therefore, appropriate strategic interventions that retain women in the continuum of maternity care by targeting those factors were recommended to be in place by different stakeholders. Moreover, health offices should monitor the completion of the continuum of care as a performance measuring indicator as it is a priority agenda for quality service assurance and empower all healthcare providers including health extension workers and women development armies to help women to initiate early antenatal care visits, and conduct pregnant women's conference to increase uptake of the continuum of maternity care.

## Data availability statement

The original contributions presented in the study are included in the article/Supplementary material, further inquiries can be directed to the corresponding author.

## Ethics statement

The studies involving human participants were reviewed and approved by an ethical clearance letter with a Ref. No. of PGC/167/2021 was obtained from Institutional Review Board of Ambo University, College of Medicine and Health Sciences. The patients/participants provided their written informed consent to participate in this study.

## Author contributions

TB, NW, and GG conceived study protocol, participated in study design, analysis, report writing, and drafted the manuscript. FW, DD, and GM involved in analysis, report writing, and drafted the manuscript. All authors have read and approved the final manuscript.
