# Supplementary material for: Completion of the continuum of maternity care and associated factors among women who gave birth in the last 6 months in Chelia district, West Shoa zone, Ethiopia: A community-based cross-sectional study
Source: Front Public Health. 2023 Jan 4;10:1026236. doi: 10.3389/fpubh.2022.1026236 (PMC9845613; doi:10.3389/fpubh.2022.1026236)
Supplement: Supplementary file 1 [file Data_Sheet_1.docx]

LOGISTIC REGRESSION VARIABLES CoC12

/METHOD=BSTEP(LR) residence time2reach when2begin culturevsplastpregnwtoldpregnctoldbpcrppartneraccplacedeliv be4dischar informed2r Monthly_incomeAgePregCatFirstANCBookingBPCRPKnowledgeModeTranspoCatEducWomenCat EducHusbCat2 AttendPWCLastOccupWomLastOccupHusbcattReceivedadequateANCServicesNumber_of_live_children

/CONTRAST (placedeliv)=Indicator

/CONTRAST (Monthly_income)=Indicator(1)

/CONTRAST (AgePregCat)=Indicator(1)

/CONTRAST (ModeTranspoCat)=Indicator(1)

/CONTRAST (EducWomenCat)=Indicator(1)

/CONTRAST (EducHusbCat2)=Indicator(1)

/CONTRAST (Number_of_live_children)=Indicator(1)

/PRINT=GOODFIT CORR CI(95)

/CRITERIA=PIN(0.05) POUT(0.10) ITERATE(20) CUT(0.5).

Logistic Regression

| Notes | | |
| --- | --- | --- |
| Output Created | | 26-OCT-2022 21:01:01 |
| Comments | |  |
| Input | Data | C:\Users\user\OneDrive\Maternity CoC\Thesis\Manuscript\PlosOne\Data set.sav |
|  | Active Dataset | DataSet1 |
|  | Filter | <none> |
|  | Weight | <none> |
|  | Split File | <none> |
|  | N of Rows in Working Data File | 428 |
| Missing Value Handling | Definition of Missing | User-defined missing values are treated as missing |
| Syntax | | LOGISTIC REGRESSION VARIABLES CoC12  /METHOD=BSTEP(LR) residence time2reach when2begin culturevsplastpregnwtoldpregnctoldbpcrppartneraccplacedeliv be4dischar informed2r Monthly_incomeAgePregCatFirstANCBookingBPCRPKnowledgeModeTranspoCatEducWomenCat EducHusbCat2 AttendPWCLastOccupWomLastOccupHusbcattReceivedadequateANCServicesNumber_of_live_children  /CONTRAST (placedeliv)=Indicator  /CONTRAST (Monthly_income)=Indicator(1)  /CONTRAST (AgePregCat)=Indicator(1)  /CONTRAST (ModeTranspoCat)=Indicator(1)  /CONTRAST (EducWomenCat)=Indicator(1)  /CONTRAST (EducHusbCat2)=Indicator(1)  /CONTRAST (Number_of_live_children)=Indicator(1)  /PRINT=GOODFIT CORR CI(95)  /CRITERIA=PIN(0.05) POUT(0.10) ITERATE(20) CUT(0.5). |
| Resources | Processor Time | 00:00:03.75 |
|  | Elapsed Time | 00:00:03.71 |

[DataSet1] C:\Users\user\OneDrive\Maternity CoC\Thesis\Manuscript\PlosOne\Data set.sav

| Case Processing Summary | | | |
| --- | --- | --- | --- |
| UnweightedCases^a^ | | N | Percent |
| Selected Cases | Included in Analysis | 342 | 79.9 |
|  | Missing Cases | 86 | 20.1 |
|  | Total | 428 | 100.0 |
| Unselected Cases | | 0 | .0 |
| Total | | 428 | 100.0 |
| a. If weight is in effect, see classification table for the total number of cases. | | | |

| Dependent Variable Encoding | |
| --- | --- |
| Original Value | Internal Value |
| No | 0 |
| Yes | 1 |

| Categorical Variables Codings | | | | |
| --- | --- | --- | --- | --- |
|  | | Frequency | Parameter coding | |
|  |  |  | (1) | (2) |
| Number_of_live_children | 1-2 children | 156 | .000 | .000 |
|  | 3-4 children | 144 | 1.000 | .000 |
|  | >/= 5 children | 42 | .000 | 1.000 |
| HH Average monthly income | </= 1000 | 66 | .000 | .000 |
|  | 1001-2000 | 92 | 1.000 | .000 |
|  | >2000 | 184 | .000 | 1.000 |
| ModeTranspoCat | Walking on foot | 243 | .000 | .000 |
|  | Public transport | 42 | 1.000 | .000 |
|  | Ambulance | 57 | .000 | 1.000 |
| EducWomenCat | Never attend school | 71 | .000 | .000 |
|  | Primary(1-8) | 132 | 1.000 | .000 |
|  | Secondary and above | 139 | .000 | 1.000 |
| EducHusbCat2 | Never attend school | 48 | .000 | .000 |
|  | Primary(1-8) | 137 | 1.000 | .000 |
|  | Secondary and above | 157 | .000 | 1.000 |
| Place of childbirth | Hospital | 127 | 1.000 | .000 |
|  | HealthCenter | 210 | .000 | 1.000 |
|  | PrivateClinic | 5 | .000 | .000 |
| AgePregCat | < 20 years | 69 | .000 |  |
|  | >/= 20 years | 273 | 1.000 |  |

Block 0: Beginning Block

| Classification Table^a,b^ | | | | | |
| --- | --- | --- | --- | --- | --- |
|  | Observed | | Predicted | | |
|  |  |  | Complete Maternity Continuum of Care | | Percentage Correct |
|  |  |  | No | Yes |  |
| Step 0 | Complete Maternity Continuum of Care | No | 252 | 0 | 100.0 |
|  |  | Yes | 90 | 0 | .0 |
|  | Overall Percentage | |  |  | 73.7 |
| a. Constant is included in the model. | | | | | |
| b. The cut value is .500 | | | | | |

| Variables in the Equation | | | | | | | |
| --- | --- | --- | --- | --- | --- | --- | --- |
|  | | B | S.E. | Wald | Df | Sig. | Exp(B) |
| Step 0 | Constant | -1.030 | .123 | 70.302 | 1 | .000 | .357 |

| Variables not in the Equation | | | | | |
| --- | --- | --- | --- | --- | --- |
|  | | | Score | df | Sig. |
| Step 0 | Variables | residence | .000 | 1 | .989 |
|  |  | time2reach | 17.549 | 1 | .000 |
|  |  | when2begin | 1.273 | 1 | .259 |
|  |  | culturevsp | 20.097 | 1 | .000 |
|  |  | lastpregnw | 8.498 | 1 | .004 |
|  |  | toldpregnc | 10.028 | 1 | .002 |
|  |  | toldbpcrp | 20.648 | 1 | .000 |
|  |  | partneracc | 54.486 | 1 | .000 |
|  |  | placedeliv | 4.224 | 2 | .121 |
|  |  | placedeliv(1) | 2.796 | 1 | .095 |
|  |  | placedeliv(2) | 1.763 | 1 | .184 |
|  |  | be4dischar | 12.133 | 1 | .000 |
|  |  | informed2r | 22.262 | 1 | .000 |
|  |  | Monthly_income | 7.300 | 2 | .026 |
|  |  | Monthly_income(1) | 1.360 | 1 | .244 |
|  |  | Monthly_income(2) | 6.790 | 1 | .009 |
|  |  | AgePregCat(1) | 2.491 | 1 | .115 |
|  |  | FirstANCBooking | 53.042 | 1 | .000 |
|  |  | BPCRPKnowledge | 2.450 | 1 | .118 |
|  |  | ModeTranspoCat | 24.531 | 2 | .000 |
|  |  | ModeTranspoCat(1) | 1.304 | 1 | .253 |
|  |  | ModeTranspoCat(2) | 24.429 | 1 | .000 |
|  |  | EducWomenCat | 19.582 | 2 | .000 |
|  |  | EducWomenCat(1) | 2.094 | 1 | .148 |
|  |  | EducWomenCat(2) | 16.855 | 1 | .000 |
|  |  | EducHusbCat2 | 11.999 | 2 | .002 |
|  |  | EducHusbCat2(1) | 4.072 | 1 | .044 |
|  |  | EducHusbCat2(2) | 11.371 | 1 | .001 |
|  |  | AttendPWCLast | 97.528 | 1 | .000 |
|  |  | OccupWomLast | 6.119 | 1 | .013 |
|  |  | OccupHusbcatt | 15.494 | 1 | .000 |
|  |  | ReceivedadequateANCServices | 8.880 | 1 | .003 |
|  |  | Number_of_live_children | 4.648 | 2 | .098 |
|  |  | Number_of_live_children(1) | .938 | 1 | .333 |
|  |  | Number_of_live_children(2) | 2.299 | 1 | .129 |
|  | Overall Statistics | | 167.434 | 29 | .000 |

Block 1: Method = Backward Stepwise (Likelihood Ratio)

| Omnibus Tests of Model Coefficients | | | | |
| --- | --- | --- | --- | --- |
|  | | Chi-square | df | Sig. |
| Step 1 | Step | 206.652 | 29 | .000 |
|  | Block | 206.652 | 29 | .000 |
|  | Model | 206.652 | 29 | .000 |
| Step 2^a^ | Step | -.008 | 1 | .931 |
|  | Block | 206.645 | 28 | .000 |
|  | Model | 206.645 | 27 | .000 |
| Step 3^a^ | Step | -.264 | 1 | .607 |
|  | Block | 206.380 | 27 | .000 |
|  | Model | 206.380 | 26 | .000 |
| Step 4^a^ | Step | -.188 | 1 | .665 |
|  | Block | 206.193 | 26 | .000 |
|  | Model | 206.193 | 25 | .000 |
| Step 5^a^ | Step | -.321 | 1 | .571 |
|  | Block | 205.872 | 25 | .000 |
|  | Model | 205.872 | 24 | .000 |
| Step 6^a^ | Step | -.257 | 1 | .612 |
|  | Block | 205.614 | 24 | .000 |
|  | Model | 205.614 | 23 | .000 |
| Step 7^a^ | Step | -.390 | 1 | .532 |
|  | Block | 205.224 | 23 | .000 |
|  | Model | 205.224 | 22 | .000 |
| Step 8^a^ | Step | -.456 | 1 | .500 |
|  | Block | 204.768 | 22 | .000 |
|  | Model | 204.768 | 21 | .000 |
| Step 9^a^ | Step | -.474 | 1 | .491 |
|  | Block | 204.294 | 21 | .000 |
|  | Model | 204.294 | 20 | .000 |
| Step 10^a^ | Step | -.468 | 1 | .494 |
|  | Block | 203.825 | 20 | .000 |
|  | Model | 203.825 | 19 | .000 |
| Step 11^a^ | Step | -1.915 | 2 | .384 |
|  | Block | 201.911 | 18 | .000 |
|  | Model | 201.911 | 18 | .000 |
| Step 12^a^ | Step | -2.809 | 2 | .245 |
|  | Block | 199.101 | 16 | .000 |
|  | Model | 199.101 | 16 | .000 |
| Step 13^a^ | Step | -1.460 | 1 | .227 |
|  | Block | 197.641 | 15 | .000 |
|  | Model | 197.641 | 14 | .000 |
| Step 14^a^ | Step | -2.798 | 2 | .247 |
|  | Block | 194.843 | 13 | .000 |
|  | Model | 194.843 | 13 | .000 |
| Step 15^a^ | Step | -2.667 | 1 | .102 |
|  | Block | 192.176 | 12 | .000 |
|  | Model | 192.176 | 11 | .000 |
| a. A negative Chi-squares value indicates that the Chi-squares value has decreased from the previous step. | | | | |

| Model Summary | | | |
| --- | --- | --- | --- |
| Step | -2 Log likelihood | Cox & Snell R Square | Nagelkerke R Square |
| 1 | 187.560^a^ | .454 | .663 |
| 2 | 187.568^a^ | .454 | .663 |
| 3 | 187.832^a^ | .453 | .662 |
| 4 | 188.020^a^ | .453 | .662 |
| 5 | 188.341^a^ | .452 | .661 |
| 6 | 188.598^a^ | .452 | .660 |
| 7 | 188.989^a^ | .451 | .659 |
| 8 | 189.444^a^ | .450 | .658 |
| 9 | 189.919^a^ | .450 | .657 |
| 10 | 190.387^a^ | .449 | .656 |
| 11 | 192.302^b^ | .446 | .652 |
| 12 | 195.111^b^ | .441 | .645 |
| 13 | 196.571^b^ | .439 | .642 |
| 14 | 199.370^b^ | .434 | .635 |
| 15 | 202.037^b^ | .430 | .628 |
| a. Estimation terminated at iteration number 20 because maximum iterations has been reached. Final solution cannot be found. | | | |
| b. Estimation terminated at iteration number 7 because parameter estimates changed by less than .001. | | | |

| Hosmer and Lemeshow Test | | | |
| --- | --- | --- | --- |
| Step | Chi-square | df | Sig. |
| 1 | 5.931 | 8 | .655 |
| 2 | 5.972 | 8 | .650 |
| 3 | 4.314 | 8 | .828 |
| 4 | 4.529 | 8 | .807 |
| 5 | 2.898 | 8 | .941 |
| 6 | 4.128 | 8 | .845 |
| 7 | 6.459 | 8 | .596 |
| 8 | 6.037 | 8 | .643 |
| 9 | 3.568 | 8 | .894 |
| 10 | 7.294 | 8 | .505 |
| 11 | 8.013 | 8 | .432 |
| 12 | 7.589 | 8 | .475 |
| 13 | 7.268 | 8 | .508 |
| 14 | 7.069 | 8 | .529 |
| 15 | 11.673 | 8 | .166 |

| Contingency Table for Hosmer and Lemeshow Test | | | | | | |
| --- | --- | --- | --- | --- | --- | --- |
|  | | Complete Maternity Continuum of Care = No | | Complete Maternity Continuum of Care = Yes | | Total |
|  |  | Observed | Expected | Observed | Expected |  |
| Step 1 | 1 | 34 | 33.976 | 0 | .024 | 34 |
|  | 2 | 34 | 33.864 | 0 | .136 | 34 |
|  | 3 | 33 | 33.623 | 1 | .377 | 34 |
|  | 4 | 33 | 33.036 | 1 | .964 | 34 |
|  | 5 | 32 | 31.780 | 2 | 2.220 | 34 |
|  | 6 | 31 | 30.004 | 3 | 3.996 | 34 |
|  | 7 | 23 | 26.402 | 11 | 7.598 | 34 |
|  | 8 | 23 | 18.848 | 11 | 15.152 | 34 |
|  | 9 | 8 | 8.734 | 26 | 25.266 | 34 |
|  | 10 | 1 | 1.734 | 35 | 34.266 | 36 |
| Step 2 | 1 | 34 | 33.976 | 0 | .024 | 34 |
|  | 2 | 34 | 33.865 | 0 | .135 | 34 |
|  | 3 | 33 | 33.625 | 1 | .375 | 34 |
|  | 4 | 33 | 33.038 | 1 | .962 | 34 |
|  | 5 | 32 | 31.783 | 2 | 2.217 | 34 |
|  | 6 | 31 | 29.996 | 3 | 4.004 | 34 |
|  | 7 | 23 | 26.409 | 11 | 7.591 | 34 |
|  | 8 | 23 | 18.836 | 11 | 15.164 | 34 |
|  | 9 | 8 | 8.732 | 26 | 25.268 | 34 |
|  | 10 | 1 | 1.741 | 35 | 34.259 | 36 |
| Step 3 | 1 | 34 | 33.976 | 0 | .024 | 34 |
|  | 2 | 34 | 33.867 | 0 | .133 | 34 |
|  | 3 | 34 | 33.627 | 0 | .373 | 34 |
|  | 4 | 32 | 32.991 | 2 | 1.009 | 34 |
|  | 5 | 32 | 31.824 | 2 | 2.176 | 34 |
|  | 6 | 31 | 29.969 | 3 | 4.031 | 34 |
|  | 7 | 24 | 26.349 | 10 | 7.651 | 34 |
|  | 8 | 22 | 18.931 | 12 | 15.069 | 34 |
|  | 9 | 8 | 8.733 | 26 | 25.267 | 34 |
|  | 10 | 1 | 1.733 | 35 | 34.267 | 36 |
| Step 4 | 1 | 34 | 33.976 | 0 | .024 | 34 |
|  | 2 | 34 | 33.865 | 0 | .135 | 34 |
|  | 3 | 34 | 33.617 | 0 | .383 | 34 |
|  | 4 | 32 | 32.964 | 2 | 1.036 | 34 |
|  | 5 | 31 | 31.773 | 3 | 2.227 | 34 |
|  | 6 | 32 | 29.963 | 2 | 4.037 | 34 |
|  | 7 | 24 | 26.411 | 10 | 7.589 | 34 |
|  | 8 | 21 | 18.995 | 13 | 15.005 | 34 |
|  | 9 | 8 | 8.734 | 26 | 25.266 | 34 |
|  | 10 | 2 | 1.702 | 34 | 34.298 | 36 |
| Step 5 | 1 | 34 | 33.973 | 0 | .027 | 34 |
|  | 2 | 34 | 33.862 | 0 | .138 | 34 |
|  | 3 | 34 | 33.610 | 0 | .390 | 34 |
|  | 4 | 32 | 32.929 | 2 | 1.071 | 34 |
|  | 5 | 32 | 31.773 | 2 | 2.227 | 34 |
|  | 6 | 31 | 29.966 | 3 | 4.034 | 34 |
|  | 7 | 24 | 26.453 | 10 | 7.547 | 34 |
|  | 8 | 20 | 19.142 | 14 | 14.858 | 34 |
|  | 9 | 9 | 8.533 | 25 | 25.467 | 34 |
|  | 10 | 2 | 1.761 | 34 | 34.239 | 36 |
| Step 6 | 1 | 34 | 33.972 | 0 | .028 | 34 |
|  | 2 | 34 | 33.864 | 0 | .136 | 34 |
|  | 3 | 34 | 33.603 | 0 | .397 | 34 |
|  | 4 | 32 | 32.950 | 2 | 1.050 | 34 |
|  | 5 | 31 | 31.774 | 3 | 2.226 | 34 |
|  | 6 | 32 | 29.954 | 2 | 4.046 | 34 |
|  | 7 | 24 | 26.475 | 10 | 7.525 | 34 |
|  | 8 | 20 | 19.043 | 14 | 14.957 | 34 |
|  | 9 | 9 | 8.603 | 25 | 25.397 | 34 |
|  | 10 | 2 | 1.762 | 34 | 34.238 | 36 |
| Step 7 | 1 | 34 | 33.972 | 0 | .028 | 34 |
|  | 2 | 34 | 33.860 | 0 | .140 | 34 |
|  | 3 | 34 | 33.612 | 0 | .388 | 34 |
|  | 4 | 32 | 32.983 | 2 | 1.017 | 34 |
|  | 5 | 30 | 31.783 | 4 | 2.217 | 34 |
|  | 6 | 33 | 29.984 | 1 | 4.016 | 34 |
|  | 7 | 25 | 26.261 | 9 | 7.739 | 34 |
|  | 8 | 20 | 19.077 | 14 | 14.923 | 34 |
|  | 9 | 9 | 8.602 | 25 | 25.398 | 34 |
|  | 10 | 1 | 1.865 | 35 | 34.135 | 36 |
| Step 8 | 1 | 34 | 33.972 | 0 | .028 | 34 |
|  | 2 | 34 | 33.862 | 0 | .138 | 34 |
|  | 3 | 34 | 33.594 | 0 | .406 | 34 |
|  | 4 | 32 | 32.969 | 2 | 1.031 | 34 |
|  | 5 | 30 | 31.787 | 4 | 2.213 | 34 |
|  | 6 | 33 | 30.031 | 1 | 3.969 | 34 |
|  | 7 | 25 | 26.240 | 9 | 7.760 | 34 |
|  | 8 | 20 | 18.976 | 14 | 15.024 | 34 |
|  | 9 | 8 | 8.684 | 26 | 25.316 | 34 |
|  | 10 | 2 | 1.885 | 34 | 34.115 | 36 |
| Step 9 | 1 | 34 | 33.974 | 0 | .026 | 34 |
|  | 2 | 34 | 33.865 | 0 | .135 | 34 |
|  | 3 | 34 | 33.602 | 0 | .398 | 34 |
|  | 4 | 32 | 32.965 | 2 | 1.035 | 34 |
|  | 5 | 31 | 31.877 | 3 | 2.123 | 34 |
|  | 6 | 32 | 29.854 | 2 | 4.146 | 34 |
|  | 7 | 25 | 26.225 | 9 | 7.775 | 34 |
|  | 8 | 20 | 19.059 | 14 | 14.941 | 34 |
|  | 9 | 8 | 8.651 | 26 | 25.349 | 34 |
|  | 10 | 2 | 1.928 | 34 | 34.072 | 36 |
| Step 10 | 1 | 34 | 33.971 | 0 | .029 | 34 |
|  | 2 | 34 | 33.857 | 0 | .143 | 34 |
|  | 3 | 34 | 33.558 | 0 | .442 | 34 |
|  | 4 | 31 | 32.923 | 3 | 1.077 | 34 |
|  | 5 | 32 | 31.903 | 2 | 2.097 | 34 |
|  | 6 | 32 | 29.834 | 2 | 4.166 | 34 |
|  | 7 | 24 | 26.322 | 10 | 7.678 | 34 |
|  | 8 | 21 | 19.221 | 13 | 14.779 | 34 |
|  | 9 | 9 | 8.444 | 25 | 25.556 | 34 |
|  | 10 | 1 | 1.967 | 35 | 34.033 | 36 |
| Step 11 | 1 | 34 | 33.957 | 0 | .043 | 34 |
|  | 2 | 35 | 34.824 | 0 | .176 | 35 |
|  | 3 | 33 | 33.495 | 1 | .505 | 34 |
|  | 4 | 33 | 33.767 | 2 | 1.233 | 35 |
|  | 5 | 31 | 31.725 | 3 | 2.275 | 34 |
|  | 6 | 33 | 29.686 | 1 | 4.314 | 34 |
|  | 7 | 23 | 26.758 | 12 | 8.242 | 35 |
|  | 8 | 22 | 18.777 | 13 | 16.223 | 35 |
|  | 9 | 7 | 7.537 | 27 | 26.463 | 34 |
|  | 10 | 1 | 1.473 | 31 | 30.527 | 32 |
| Step 12 | 1 | 34 | 33.940 | 0 | .060 | 34 |
|  | 2 | 34 | 33.800 | 0 | .200 | 34 |
|  | 3 | 32 | 33.425 | 2 | .575 | 34 |
|  | 4 | 33 | 32.822 | 1 | 1.178 | 34 |
|  | 5 | 32 | 31.743 | 2 | 2.257 | 34 |
|  | 6 | 30 | 29.406 | 4 | 4.594 | 34 |
|  | 7 | 25 | 26.685 | 9 | 7.315 | 34 |
|  | 8 | 23 | 19.866 | 12 | 15.134 | 35 |
|  | 9 | 9 | 8.499 | 25 | 25.501 | 34 |
|  | 10 | 0 | 1.813 | 35 | 33.187 | 35 |
| Step 13 | 1 | 34 | 33.936 | 0 | .064 | 34 |
|  | 2 | 34 | 33.793 | 0 | .207 | 34 |
|  | 3 | 33 | 33.375 | 1 | .625 | 34 |
|  | 4 | 32 | 32.764 | 2 | 1.236 | 34 |
|  | 5 | 33 | 31.852 | 1 | 2.148 | 34 |
|  | 6 | 30 | 29.535 | 4 | 4.465 | 34 |
|  | 7 | 23 | 26.508 | 11 | 7.492 | 34 |
|  | 8 | 24 | 19.211 | 10 | 14.789 | 34 |
|  | 9 | 8 | 8.979 | 26 | 25.021 | 34 |
|  | 10 | 1 | 2.048 | 35 | 33.952 | 36 |
| Step 14 | 1 | 34 | 33.930 | 0 | .070 | 34 |
|  | 2 | 35 | 34.734 | 0 | .266 | 35 |
|  | 3 | 32 | 33.342 | 2 | .658 | 34 |
|  | 4 | 33 | 32.709 | 1 | 1.291 | 34 |
|  | 5 | 33 | 32.422 | 2 | 2.578 | 35 |
|  | 6 | 28 | 29.344 | 6 | 4.656 | 34 |
|  | 7 | 26 | 26.408 | 8 | 7.592 | 34 |
|  | 8 | 22 | 18.752 | 12 | 15.248 | 34 |
|  | 9 | 9 | 8.506 | 25 | 25.494 | 34 |
|  | 10 | 0 | 1.852 | 34 | 32.148 | 34 |
| Step 15 | 1 | 35 | 34.929 | 0 | .071 | 35 |
|  | 2 | 34 | 33.711 | 0 | .289 | 34 |
|  | 3 | 32 | 33.276 | 2 | .724 | 34 |
|  | 4 | 33 | 32.476 | 1 | 1.524 | 34 |
|  | 5 | 33 | 31.429 | 1 | 2.571 | 34 |
|  | 6 | 25 | 29.094 | 9 | 4.906 | 34 |
|  | 7 | 27 | 26.743 | 7 | 7.257 | 34 |
|  | 8 | 24 | 20.018 | 11 | 14.982 | 35 |
|  | 9 | 9 | 8.531 | 25 | 25.469 | 34 |
|  | 10 | 0 | 1.793 | 34 | 32.207 | 34 |

| Classification Table^a^ | | | | | |
| --- | --- | --- | --- | --- | --- |
|  | Observed | | Predicted | | |
|  |  |  | Complete Maternity Continuum of Care | | Percentage Correct |
|  |  |  | No | Yes |  |
| Step 1 | Complete Maternity Continuum of Care | No | 235 | 17 | 93.3 |
|  |  | Yes | 25 | 65 | 72.2 |
|  | Overall Percentage | |  |  | 87.7 |
| Step 2 | Complete Maternity Continuum of Care | No | 236 | 16 | 93.7 |
|  |  | Yes | 25 | 65 | 72.2 |
|  | Overall Percentage | |  |  | 88.0 |
| Step 3 | Complete Maternity Continuum of Care | No | 236 | 16 | 93.7 |
|  |  | Yes | 25 | 65 | 72.2 |
|  | Overall Percentage | |  |  | 88.0 |
| Step 4 | Complete Maternity Continuum of Care | No | 236 | 16 | 93.7 |
|  |  | Yes | 25 | 65 | 72.2 |
|  | Overall Percentage | |  |  | 88.0 |
| Step 5 | Complete Maternity Continuum of Care | No | 236 | 16 | 93.7 |
|  |  | Yes | 25 | 65 | 72.2 |
|  | Overall Percentage | |  |  | 88.0 |
| Step 6 | Complete Maternity Continuum of Care | No | 235 | 17 | 93.3 |
|  |  | Yes | 25 | 65 | 72.2 |
|  | Overall Percentage | |  |  | 87.7 |
| Step 7 | Complete Maternity Continuum of Care | No | 237 | 15 | 94.0 |
|  |  | Yes | 25 | 65 | 72.2 |
|  | Overall Percentage | |  |  | 88.3 |
| Step 8 | Complete Maternity Continuum of Care | No | 237 | 15 | 94.0 |
|  |  | Yes | 25 | 65 | 72.2 |
|  | Overall Percentage | |  |  | 88.3 |
| Step 9 | Complete Maternity Continuum of Care | No | 237 | 15 | 94.0 |
|  |  | Yes | 27 | 63 | 70.0 |
|  | Overall Percentage | |  |  | 87.7 |
| Step 10 | Complete Maternity Continuum of Care | No | 235 | 17 | 93.3 |
|  |  | Yes | 26 | 64 | 71.1 |
|  | Overall Percentage | |  |  | 87.4 |
| Step 11 | Complete Maternity Continuum of Care | No | 236 | 16 | 93.7 |
|  |  | Yes | 27 | 63 | 70.0 |
|  | Overall Percentage | |  |  | 87.4 |
| Step 12 | Complete Maternity Continuum of Care | No | 239 | 13 | 94.8 |
|  |  | Yes | 28 | 62 | 68.9 |
|  | Overall Percentage | |  |  | 88.0 |
| Step 13 | Complete Maternity Continuum of Care | No | 235 | 17 | 93.3 |
|  |  | Yes | 29 | 61 | 67.8 |
|  | Overall Percentage | |  |  | 86.5 |
| Step 14 | Complete Maternity Continuum of Care | No | 237 | 15 | 94.0 |
|  |  | Yes | 29 | 61 | 67.8 |
|  | Overall Percentage | |  |  | 87.1 |
| Step 15 | Complete Maternity Continuum of Care | No | 237 | 15 | 94.0 |
|  |  | Yes | 29 | 61 | 67.8 |
|  | Overall Percentage | |  |  | 87.1 |
| a. The cut value is .500 | | | | | |

| Variables in the Equation | | | | | | | | | |
| --- | --- | --- | --- | --- | --- | --- | --- | --- | --- |
|  | | B | S.E. | Wald | df | Sig. | Exp(B) | 95% C.I.for EXP(B) | |
|  |  |  |  |  |  |  |  | Lower | Upper |
| Step 1^a^ | residence | .848 | .596 | 2.025 | 1 | .155 | 2.335 | .726 | 7.505 |
|  | time2reach | 1.459 | .530 | 7.577 | 1 | .006 | 4.304 | 1.522 | 12.166 |
|  | when2begin | .307 | .398 | .596 | 1 | .440 | 1.360 | .623 | 2.965 |
|  | culturevsp | .040 | .456 | .008 | 1 | .931 | 1.041 | .426 | 2.543 |
|  | lastpregnw | 1.260 | .645 | 3.813 | 1 | .051 | 3.527 | .995 | 12.495 |
|  | toldpregnc | .486 | .803 | .366 | 1 | .545 | 1.626 | .337 | 7.852 |
|  | toldbpcrp | .960 | .753 | 1.626 | 1 | .202 | 2.612 | .597 | 11.427 |
|  | partneracc | 1.515 | .456 | 11.032 | 1 | .001 | 4.548 | 1.861 | 11.116 |
|  | placedeliv |  |  | 1.864 | 2 | .394 |  |  |  |
|  | placedeliv(1) | 19.806 | 15361.215 | .000 | 1 | .999 | 399652437.026 | .000 | . |
|  | placedeliv(2) | 19.183 | 15361.215 | .000 | 1 | .999 | 214336251.749 | .000 | . |
|  | be4dischar | .472 | .603 | .613 | 1 | .434 | 1.604 | .492 | 5.231 |
|  | informed2r | .950 | .511 | 3.451 | 1 | .063 | 2.586 | .949 | 7.045 |
|  | Monthly_income |  |  | 2.905 | 2 | .234 |  |  |  |
|  | Monthly_income(1) | .167 | .683 | .060 | 1 | .807 | 1.181 | .310 | 4.508 |
|  | Monthly_income(2) | .940 | .648 | 2.104 | 1 | .147 | 2.559 | .719 | 9.110 |
|  | AgePregCat(1) | .348 | .518 | .450 | 1 | .503 | 1.416 | .512 | 3.911 |
|  | FirstANCBooking | 1.142 | .449 | 6.487 | 1 | .011 | 3.134 | 1.301 | 7.551 |
|  | BPCRPKnowledge | -.325 | .488 | .443 | 1 | .506 | .723 | .278 | 1.881 |
|  | ModeTranspoCat |  |  | 3.437 | 2 | .179 |  |  |  |
|  | ModeTranspoCat(1) | -.123 | .617 | .039 | 1 | .842 | .885 | .264 | 2.964 |
|  | ModeTranspoCat(2) | 1.096 | .619 | 3.133 | 1 | .077 | 2.993 | .889 | 10.079 |
|  | EducWomenCat |  |  | 3.218 | 2 | .200 |  |  |  |
|  | EducWomenCat(1) | .259 | .646 | .161 | 1 | .688 | 1.296 | .365 | 4.601 |
|  | EducWomenCat(2) | 1.057 | .701 | 2.276 | 1 | .131 | 2.877 | .729 | 11.355 |
|  | EducHusbCat2 |  |  | 2.462 | 2 | .292 |  |  |  |
|  | EducHusbCat2(1) | .888 | .791 | 1.259 | 1 | .262 | 2.429 | .515 | 11.453 |
|  | EducHusbCat2(2) | 1.292 | .825 | 2.454 | 1 | .117 | 3.639 | .723 | 18.322 |
|  | AttendPWCLast | 2.584 | .485 | 28.397 | 1 | .000 | 13.255 | 5.123 | 34.290 |
|  | OccupWomLast | -.448 | .764 | .343 | 1 | .558 | .639 | .143 | 2.857 |
|  | OccupHusbcatt | .316 | .612 | .267 | 1 | .606 | 1.371 | .413 | 4.549 |
|  | ReceivedadequateANCServices | -.435 | .660 | .435 | 1 | .509 | .647 | .177 | 2.359 |
|  | Number_of_live_children |  |  | 3.732 | 2 | .155 |  |  |  |
|  | Number_of_live_children(1) | -.660 | .454 | 2.120 | 1 | .145 | .517 | .212 | 1.257 |
|  | Number_of_live_children(2) | -1.277 | .781 | 2.677 | 1 | .102 | .279 | .060 | 1.288 |
|  | Constant | -30.826 | 15361.215 | .000 | 1 | .998 | .000 |  |  |
| Step 2^a^ | residence | .848 | .596 | 2.023 | 1 | .155 | 2.334 | .726 | 7.504 |
|  | time2reach | 1.459 | .530 | 7.584 | 1 | .006 | 4.303 | 1.523 | 12.155 |
|  | when2begin | .311 | .395 | .621 | 1 | .431 | 1.365 | .630 | 2.960 |
|  | lastpregnw | 1.268 | .640 | 3.919 | 1 | .048 | 3.552 | 1.013 | 12.462 |
|  | toldpregnc | .484 | .802 | .365 | 1 | .546 | 1.623 | .337 | 7.819 |
|  | toldbpcrp | .960 | .753 | 1.628 | 1 | .202 | 2.612 | .598 | 11.419 |
|  | partneracc | 1.524 | .444 | 11.793 | 1 | .001 | 4.590 | 1.923 | 10.953 |
|  | placedeliv |  |  | 1.909 | 2 | .385 |  |  |  |
|  | placedeliv(1) | 19.793 | 15371.079 | .000 | 1 | .999 | 394345220.333 | .000 | . |
|  | placedeliv(2) | 19.166 | 15371.079 | .000 | 1 | .999 | 210623857.589 | .000 | . |
|  | be4dischar | .481 | .594 | .656 | 1 | .418 | 1.618 | .505 | 5.189 |
|  | informed2r | .951 | .512 | 3.456 | 1 | .063 | 2.588 | .950 | 7.053 |
|  | Monthly_income |  |  | 2.916 | 2 | .233 |  |  |  |
|  | Monthly_income(1) | .167 | .683 | .060 | 1 | .807 | 1.182 | .310 | 4.509 |
|  | Monthly_income(2) | .934 | .645 | 2.097 | 1 | .148 | 2.545 | .719 | 9.013 |
|  | AgePregCat(1) | .351 | .517 | .460 | 1 | .498 | 1.420 | .515 | 3.916 |
|  | FirstANCBooking | 1.145 | .448 | 6.549 | 1 | .010 | 3.144 | 1.308 | 7.557 |
|  | BPCRPKnowledge | -.326 | .488 | .448 | 1 | .504 | .722 | .277 | 1.877 |
|  | ModeTranspoCat |  |  | 3.512 | 2 | .173 |  |  |  |
|  | ModeTranspoCat(1) | -.125 | .617 | .041 | 1 | .840 | .883 | .263 | 2.959 |
|  | ModeTranspoCat(2) | 1.101 | .616 | 3.193 | 1 | .074 | 3.008 | .899 | 10.064 |
|  | EducWomenCat |  |  | 3.211 | 2 | .201 |  |  |  |
|  | EducWomenCat(1) | .257 | .645 | .158 | 1 | .691 | 1.293 | .365 | 4.577 |
|  | EducWomenCat(2) | 1.053 | .699 | 2.269 | 1 | .132 | 2.867 | .728 | 11.287 |
|  | EducHusbCat2 |  |  | 2.484 | 2 | .289 |  |  |  |
|  | EducHusbCat2(1) | .885 | .789 | 1.257 | 1 | .262 | 2.422 | .516 | 11.369 |
|  | EducHusbCat2(2) | 1.294 | .823 | 2.473 | 1 | .116 | 3.647 | .727 | 18.297 |
|  | AttendPWCLast | 2.591 | .479 | 29.272 | 1 | .000 | 13.344 | 5.220 | 34.115 |
|  | OccupWomLast | -.445 | .763 | .340 | 1 | .560 | .641 | .143 | 2.860 |
|  | OccupHusbcatt | .315 | .611 | .265 | 1 | .607 | 1.370 | .413 | 4.541 |
|  | ReceivedadequateANCServices | -.434 | .659 | .433 | 1 | .511 | .648 | .178 | 2.359 |
|  | Number_of_live_children |  |  | 3.741 | 2 | .154 |  |  |  |
|  | Number_of_live_children(1) | -.651 | .441 | 2.180 | 1 | .140 | .521 | .220 | 1.238 |
|  | Number_of_live_children(2) | -1.280 | .780 | 2.690 | 1 | .101 | .278 | .060 | 1.283 |
|  | Constant | -30.827 | 15371.079 | .000 | 1 | .998 | .000 |  |  |
| Step 3^a^ | residence | .786 | .580 | 1.833 | 1 | .176 | 2.195 | .703 | 6.846 |
|  | time2reach | 1.498 | .526 | 8.121 | 1 | .004 | 4.471 | 1.596 | 12.523 |
|  | when2begin | .319 | .396 | .650 | 1 | .420 | 1.376 | .633 | 2.989 |
|  | lastpregnw | 1.267 | .638 | 3.949 | 1 | .047 | 3.552 | 1.018 | 12.398 |
|  | toldpregnc | .479 | .800 | .358 | 1 | .549 | 1.614 | .337 | 7.737 |
|  | toldbpcrp | .942 | .752 | 1.570 | 1 | .210 | 2.565 | .588 | 11.191 |
|  | partneracc | 1.541 | .442 | 12.158 | 1 | .000 | 4.668 | 1.963 | 11.100 |
|  | placedeliv |  |  | 1.940 | 2 | .379 |  |  |  |
|  | placedeliv(1) | 19.784 | 15345.395 | .000 | 1 | .999 | 390748946.910 | .000 | . |
|  | placedeliv(2) | 19.152 | 15345.395 | .000 | 1 | .999 | 207820214.494 | .000 | . |
|  | be4dischar | .446 | .588 | .577 | 1 | .448 | 1.563 | .494 | 4.945 |
|  | informed2r | .965 | .511 | 3.567 | 1 | .059 | 2.625 | .964 | 7.146 |
|  | Monthly_income |  |  | 3.115 | 2 | .211 |  |  |  |
|  | Monthly_income(1) | .168 | .684 | .060 | 1 | .806 | 1.183 | .310 | 4.516 |
|  | Monthly_income(2) | .958 | .645 | 2.207 | 1 | .137 | 2.606 | .737 | 9.218 |
|  | AgePregCat(1) | .354 | .518 | .466 | 1 | .495 | 1.425 | .516 | 3.936 |
|  | FirstANCBooking | 1.172 | .444 | 6.976 | 1 | .008 | 3.228 | 1.353 | 7.702 |
|  | BPCRPKnowledge | -.319 | .488 | .428 | 1 | .513 | .727 | .279 | 1.891 |
|  | ModeTranspoCat |  |  | 3.828 | 2 | .148 |  |  |  |
|  | ModeTranspoCat(1) | -.131 | .616 | .045 | 1 | .831 | .877 | .262 | 2.933 |
|  | ModeTranspoCat(2) | 1.137 | .611 | 3.465 | 1 | .063 | 3.118 | .942 | 10.326 |
|  | EducWomenCat |  |  | 3.009 | 2 | .222 |  |  |  |
|  | EducWomenCat(1) | .235 | .645 | .134 | 1 | .715 | 1.266 | .358 | 4.476 |
|  | EducWomenCat(2) | 1.000 | .693 | 2.080 | 1 | .149 | 2.718 | .698 | 10.577 |
|  | EducHusbCat2 |  |  | 3.321 | 2 | .190 |  |  |  |
|  | EducHusbCat2(1) | .897 | .792 | 1.283 | 1 | .257 | 2.452 | .519 | 11.578 |
|  | EducHusbCat2(2) | 1.411 | .795 | 3.150 | 1 | .076 | 4.099 | .863 | 19.460 |
|  | AttendPWCLast | 2.595 | .479 | 29.336 | 1 | .000 | 13.394 | 5.237 | 34.252 |
|  | OccupWomLast | -.309 | .716 | .186 | 1 | .666 | .734 | .180 | 2.989 |
|  | ReceivedadequateANCServices | -.421 | .661 | .405 | 1 | .524 | .657 | .180 | 2.397 |
|  | Number_of_live_children |  |  | 3.930 | 2 | .140 |  |  |  |
|  | Number_of_live_children(1) | -.663 | .440 | 2.267 | 1 | .132 | .515 | .217 | 1.221 |
|  | Number_of_live_children(2) | -1.318 | .783 | 2.835 | 1 | .092 | .268 | .058 | 1.241 |
|  | Constant | -30.731 | 15345.396 | .000 | 1 | .998 | .000 |  |  |
| Step 4^a^ | residence | .847 | .567 | 2.226 | 1 | .136 | 2.332 | .767 | 7.091 |
|  | time2reach | 1.503 | .524 | 8.238 | 1 | .004 | 4.495 | 1.611 | 12.544 |
|  | when2begin | .313 | .395 | .630 | 1 | .427 | 1.368 | .631 | 2.966 |
|  | lastpregnw | 1.261 | .638 | 3.906 | 1 | .048 | 3.530 | 1.010 | 12.331 |
|  | toldpregnc | .450 | .797 | .319 | 1 | .572 | 1.568 | .329 | 7.483 |
|  | toldbpcrp | .954 | .750 | 1.618 | 1 | .203 | 2.595 | .597 | 11.278 |
|  | partneracc | 1.504 | .432 | 12.150 | 1 | .000 | 4.501 | 1.932 | 10.489 |
|  | placedeliv |  |  | 1.829 | 2 | .401 |  |  |  |
|  | placedeliv(1) | 19.729 | 15379.564 | .000 | 1 | .999 | 369827366.343 | .000 | . |
|  | placedeliv(2) | 19.120 | 15379.564 | .000 | 1 | .999 | 201194227.820 | .000 | . |
|  | be4dischar | .471 | .586 | .647 | 1 | .421 | 1.602 | .508 | 5.049 |
|  | informed2r | .970 | .509 | 3.636 | 1 | .057 | 2.637 | .973 | 7.144 |
|  | Monthly_income |  |  | 2.981 | 2 | .225 |  |  |  |
|  | Monthly_income(1) | .185 | .681 | .074 | 1 | .786 | 1.203 | .316 | 4.574 |
|  | Monthly_income(2) | .943 | .645 | 2.138 | 1 | .144 | 2.567 | .725 | 9.086 |
|  | AgePregCat(1) | .289 | .494 | .342 | 1 | .559 | 1.335 | .507 | 3.519 |
|  | FirstANCBooking | 1.163 | .443 | 6.884 | 1 | .009 | 3.200 | 1.342 | 7.632 |
|  | BPCRPKnowledge | -.308 | .486 | .403 | 1 | .526 | .735 | .283 | 1.904 |
|  | ModeTranspoCat |  |  | 3.739 | 2 | .154 |  |  |  |
|  | ModeTranspoCat(1) | -.100 | .612 | .027 | 1 | .870 | .905 | .273 | 3.001 |
|  | ModeTranspoCat(2) | 1.129 | .610 | 3.422 | 1 | .064 | 3.093 | .935 | 10.232 |
|  | EducWomenCat |  |  | 2.813 | 2 | .245 |  |  |  |
|  | EducWomenCat(1) | .251 | .643 | .152 | 1 | .697 | 1.285 | .364 | 4.532 |
|  | EducWomenCat(2) | .956 | .687 | 1.938 | 1 | .164 | 2.601 | .677 | 9.991 |
|  | EducHusbCat2 |  |  | 3.215 | 2 | .200 |  |  |  |
|  | EducHusbCat2(1) | .897 | .791 | 1.285 | 1 | .257 | 2.452 | .520 | 11.562 |
|  | EducHusbCat2(2) | 1.389 | .793 | 3.066 | 1 | .080 | 4.010 | .847 | 18.978 |
|  | AttendPWCLast | 2.582 | .478 | 29.185 | 1 | .000 | 13.219 | 5.181 | 33.727 |
|  | ReceivedadequateANCServices | -.387 | .656 | .347 | 1 | .556 | .679 | .188 | 2.458 |
|  | Number_of_live_children |  |  | 4.266 | 2 | .118 |  |  |  |
|  | Number_of_live_children(1) | -.701 | .433 | 2.629 | 1 | .105 | .496 | .212 | 1.158 |
|  | Number_of_live_children(2) | -1.332 | .779 | 2.919 | 1 | .088 | .264 | .057 | 1.216 |
|  | Constant | -30.731 | 15379.565 | .000 | 1 | .998 | .000 |  |  |
| Step 5^a^ | residence | .825 | .563 | 2.150 | 1 | .143 | 2.283 | .757 | 6.879 |
|  | time2reach | 1.483 | .522 | 8.072 | 1 | .004 | 4.406 | 1.584 | 12.254 |
|  | when2begin | .289 | .393 | .541 | 1 | .462 | 1.335 | .618 | 2.885 |
|  | lastpregnw | 1.240 | .631 | 3.867 | 1 | .049 | 3.457 | 1.004 | 11.904 |
|  | toldbpcrp | 1.142 | .680 | 2.820 | 1 | .093 | 3.134 | .826 | 11.886 |
|  | partneracc | 1.469 | .425 | 11.945 | 1 | .001 | 4.344 | 1.889 | 9.990 |
|  | placedeliv |  |  | 1.677 | 2 | .432 |  |  |  |
|  | placedeliv(1) | 19.622 | 15326.210 | .000 | 1 | .999 | 332388107.476 | .000 | . |
|  | placedeliv(2) | 19.045 | 15326.210 | .000 | 1 | .999 | 186656040.911 | .000 | . |
|  | be4dischar | .475 | .587 | .655 | 1 | .418 | 1.608 | .509 | 5.076 |
|  | informed2r | .986 | .509 | 3.763 | 1 | .052 | 2.682 | .990 | 7.265 |
|  | Monthly_income |  |  | 2.889 | 2 | .236 |  |  |  |
|  | Monthly_income(1) | .187 | .679 | .076 | 1 | .783 | 1.206 | .319 | 4.562 |
|  | Monthly_income(2) | .930 | .643 | 2.094 | 1 | .148 | 2.534 | .719 | 8.930 |
|  | AgePregCat(1) | .291 | .494 | .346 | 1 | .556 | 1.337 | .508 | 3.522 |
|  | FirstANCBooking | 1.122 | .436 | 6.623 | 1 | .010 | 3.070 | 1.307 | 7.213 |
|  | BPCRPKnowledge | -.277 | .480 | .331 | 1 | .565 | .758 | .296 | 1.944 |
|  | ModeTranspoCat |  |  | 4.086 | 2 | .130 |  |  |  |
|  | ModeTranspoCat(1) | -.126 | .613 | .042 | 1 | .837 | .881 | .265 | 2.930 |
|  | ModeTranspoCat(2) | 1.166 | .608 | 3.678 | 1 | .055 | 3.209 | .975 | 10.564 |
|  | EducWomenCat |  |  | 2.799 | 2 | .247 |  |  |  |
|  | EducWomenCat(1) | .232 | .642 | .130 | 1 | .718 | 1.261 | .358 | 4.440 |
|  | EducWomenCat(2) | .940 | .684 | 1.887 | 1 | .170 | 2.560 | .669 | 9.789 |
|  | EducHusbCat2 |  |  | 3.115 | 2 | .211 |  |  |  |
|  | EducHusbCat2(1) | .826 | .780 | 1.121 | 1 | .290 | 2.284 | .495 | 10.529 |
|  | EducHusbCat2(2) | 1.347 | .789 | 2.917 | 1 | .088 | 3.845 | .820 | 18.032 |
|  | AttendPWCLast | 2.588 | .477 | 29.386 | 1 | .000 | 13.299 | 5.218 | 33.896 |
|  | ReceivedadequateANCServices | -.331 | .649 | .261 | 1 | .609 | .718 | .201 | 2.559 |
|  | Number_of_live_children |  |  | 4.220 | 2 | .121 |  |  |  |
|  | Number_of_live_children(1) | -.697 | .432 | 2.605 | 1 | .107 | .498 | .214 | 1.161 |
|  | Number_of_live_children(2) | -1.315 | .777 | 2.864 | 1 | .091 | .268 | .058 | 1.231 |
|  | Constant | -30.278 | 15326.210 | .000 | 1 | .998 | .000 |  |  |
| Step 6^a^ | residence | .818 | .564 | 2.101 | 1 | .147 | 2.265 | .750 | 6.844 |
|  | time2reach | 1.488 | .522 | 8.123 | 1 | .004 | 4.429 | 1.592 | 12.324 |
|  | when2begin | .253 | .387 | .427 | 1 | .513 | 1.288 | .603 | 2.751 |
|  | lastpregnw | 1.245 | .634 | 3.857 | 1 | .050 | 3.473 | 1.003 | 12.031 |
|  | toldbpcrp | 1.058 | .659 | 2.576 | 1 | .109 | 2.882 | .791 | 10.496 |
|  | partneracc | 1.448 | .422 | 11.741 | 1 | .001 | 4.253 | 1.858 | 9.734 |
|  | placedeliv |  |  | 1.545 | 2 | .462 |  |  |  |
|  | placedeliv(1) | 19.346 | 15463.078 | .000 | 1 | .999 | 252327763.754 | .000 | . |
|  | placedeliv(2) | 18.798 | 15463.078 | .000 | 1 | .999 | 145846919.277 | .000 | . |
|  | be4dischar | .419 | .577 | .528 | 1 | .467 | 1.521 | .491 | 4.714 |
|  | informed2r | .986 | .507 | 3.776 | 1 | .052 | 2.680 | .991 | 7.245 |
|  | Monthly_income |  |  | 2.914 | 2 | .233 |  |  |  |
|  | Monthly_income(1) | .226 | .674 | .112 | 1 | .738 | 1.253 | .334 | 4.696 |
|  | Monthly_income(2) | .959 | .645 | 2.213 | 1 | .137 | 2.609 | .737 | 9.231 |
|  | AgePregCat(1) | .306 | .492 | .386 | 1 | .534 | 1.358 | .518 | 3.560 |
|  | FirstANCBooking | 1.108 | .436 | 6.447 | 1 | .011 | 3.027 | 1.287 | 7.117 |
|  | BPCRPKnowledge | -.324 | .471 | .472 | 1 | .492 | .724 | .288 | 1.820 |
|  | ModeTranspoCat |  |  | 4.285 | 2 | .117 |  |  |  |
|  | ModeTranspoCat(1) | -.101 | .610 | .027 | 1 | .868 | .904 | .273 | 2.988 |
|  | ModeTranspoCat(2) | 1.198 | .604 | 3.929 | 1 | .047 | 3.314 | 1.013 | 10.835 |
|  | EducWomenCat |  |  | 2.704 | 2 | .259 |  |  |  |
|  | EducWomenCat(1) | .190 | .637 | .089 | 1 | .765 | 1.210 | .347 | 4.220 |
|  | EducWomenCat(2) | .897 | .678 | 1.748 | 1 | .186 | 2.451 | .649 | 9.262 |
|  | EducHusbCat2 |  |  | 2.951 | 2 | .229 |  |  |  |
|  | EducHusbCat2(1) | .788 | .773 | 1.041 | 1 | .308 | 2.200 | .484 | 10.006 |
|  | EducHusbCat2(2) | 1.294 | .778 | 2.766 | 1 | .096 | 3.647 | .794 | 16.752 |
|  | AttendPWCLast | 2.580 | .477 | 29.263 | 1 | .000 | 13.201 | 5.183 | 33.623 |
|  | Number_of_live_children |  |  | 4.124 | 2 | .127 |  |  |  |
|  | Number_of_live_children(1) | -.691 | .432 | 2.560 | 1 | .110 | .501 | .215 | 1.168 |
|  | Number_of_live_children(2) | -1.300 | .781 | 2.769 | 1 | .096 | .273 | .059 | 1.260 |
|  | Constant | -30.027 | 15463.078 | .000 | 1 | .998 | .000 |  |  |
| Step 7^a^ | residence | .842 | .561 | 2.252 | 1 | .133 | 2.320 | .773 | 6.967 |
|  | time2reach | 1.505 | .522 | 8.314 | 1 | .004 | 4.506 | 1.620 | 12.537 |
|  | when2begin | .261 | .387 | .453 | 1 | .501 | 1.298 | .607 | 2.773 |
|  | lastpregnw | 1.288 | .627 | 4.221 | 1 | .040 | 3.627 | 1.061 | 12.396 |
|  | toldbpcrp | 1.027 | .655 | 2.459 | 1 | .117 | 2.792 | .774 | 10.076 |
|  | partneracc | 1.422 | .419 | 11.512 | 1 | .001 | 4.147 | 1.823 | 9.430 |
|  | placedeliv |  |  | 1.348 | 2 | .510 |  |  |  |
|  | placedeliv(1) | 19.295 | 15521.381 | .000 | 1 | .999 | 239819997.330 | .000 | . |
|  | placedeliv(2) | 18.789 | 15521.381 | .000 | 1 | .999 | 144561988.664 | .000 | . |
|  | be4dischar | .410 | .575 | .508 | 1 | .476 | 1.507 | .488 | 4.651 |
|  | informed2r | .981 | .506 | 3.763 | 1 | .052 | 2.668 | .990 | 7.190 |
|  | Monthly_income |  |  | 3.175 | 2 | .204 |  |  |  |
|  | Monthly_income(1) | .271 | .671 | .163 | 1 | .687 | 1.311 | .352 | 4.884 |
|  | Monthly_income(2) | 1.013 | .640 | 2.507 | 1 | .113 | 2.755 | .786 | 9.655 |
|  | FirstANCBooking | 1.071 | .431 | 6.161 | 1 | .013 | 2.917 | 1.253 | 6.793 |
|  | BPCRPKnowledge | -.320 | .471 | .463 | 1 | .496 | .726 | .288 | 1.827 |
|  | ModeTranspoCat |  |  | 4.194 | 2 | .123 |  |  |  |
|  | ModeTranspoCat(1) | -.066 | .607 | .012 | 1 | .914 | .937 | .285 | 3.080 |
|  | ModeTranspoCat(2) | 1.194 | .605 | 3.892 | 1 | .049 | 3.301 | 1.008 | 10.814 |
|  | EducWomenCat |  |  | 3.053 | 2 | .217 |  |  |  |
|  | EducWomenCat(1) | .225 | .640 | .124 | 1 | .725 | 1.252 | .357 | 4.395 |
|  | EducWomenCat(2) | .962 | .677 | 2.018 | 1 | .155 | 2.616 | .694 | 9.860 |
|  | EducHusbCat2 |  |  | 2.730 | 2 | .255 |  |  |  |
|  | EducHusbCat2(1) | .779 | .779 | .999 | 1 | .318 | 2.178 | .473 | 10.030 |
|  | EducHusbCat2(2) | 1.252 | .782 | 2.565 | 1 | .109 | 3.497 | .756 | 16.177 |
|  | AttendPWCLast | 2.626 | .472 | 30.915 | 1 | .000 | 13.814 | 5.475 | 34.858 |
|  | Number_of_live_children |  |  | 3.974 | 2 | .137 |  |  |  |
|  | Number_of_live_children(1) | -.675 | .429 | 2.482 | 1 | .115 | .509 | .220 | 1.179 |
|  | Number_of_live_children(2) | -1.260 | .777 | 2.630 | 1 | .105 | .284 | .062 | 1.301 |
|  | Constant | -29.894 | 15521.381 | .000 | 1 | .998 | .000 |  |  |
| Step 8^a^ | residence | .799 | .556 | 2.068 | 1 | .150 | 2.224 | .748 | 6.608 |
|  | time2reach | 1.490 | .520 | 8.209 | 1 | .004 | 4.435 | 1.601 | 12.289 |
|  | lastpregnw | 1.270 | .626 | 4.114 | 1 | .043 | 3.560 | 1.044 | 12.143 |
|  | toldbpcrp | 1.014 | .653 | 2.411 | 1 | .120 | 2.755 | .767 | 9.903 |
|  | partneracc | 1.386 | .414 | 11.225 | 1 | .001 | 4.000 | 1.778 | 9.000 |
|  | placedeliv |  |  | 1.105 | 2 | .576 |  |  |  |
|  | placedeliv(1) | 19.243 | 15548.010 | .000 | 1 | .999 | 227531776.847 | .000 | . |
|  | placedeliv(2) | 18.796 | 15548.010 | .000 | 1 | .999 | 145545939.993 | .000 | . |
|  | be4dischar | .390 | .572 | .466 | 1 | .495 | 1.477 | .482 | 4.533 |
|  | informed2r | 1.013 | .506 | 4.010 | 1 | .045 | 2.755 | 1.022 | 7.427 |
|  | Monthly_income |  |  | 3.537 | 2 | .171 |  |  |  |
|  | Monthly_income(1) | .341 | .662 | .265 | 1 | .607 | 1.406 | .384 | 5.146 |
|  | Monthly_income(2) | 1.085 | .631 | 2.956 | 1 | .086 | 2.961 | .859 | 10.205 |
|  | FirstANCBooking | 1.051 | .429 | 5.985 | 1 | .014 | 2.860 | 1.232 | 6.636 |
|  | BPCRPKnowledge | -.357 | .467 | .585 | 1 | .444 | .700 | .280 | 1.747 |
|  | ModeTranspoCat |  |  | 4.082 | 2 | .130 |  |  |  |
|  | ModeTranspoCat(1) | -.021 | .601 | .001 | 1 | .972 | .979 | .301 | 3.183 |
|  | ModeTranspoCat(2) | 1.191 | .607 | 3.845 | 1 | .050 | 3.291 | 1.001 | 10.825 |
|  | EducWomenCat |  |  | 3.036 | 2 | .219 |  |  |  |
|  | EducWomenCat(1) | .251 | .637 | .155 | 1 | .694 | 1.285 | .369 | 4.478 |
|  | EducWomenCat(2) | .976 | .675 | 2.091 | 1 | .148 | 2.653 | .707 | 9.959 |
|  | EducHusbCat2 |  |  | 2.576 | 2 | .276 |  |  |  |
|  | EducHusbCat2(1) | .770 | .779 | .977 | 1 | .323 | 2.160 | .469 | 9.948 |
|  | EducHusbCat2(2) | 1.217 | .779 | 2.438 | 1 | .118 | 3.377 | .733 | 15.559 |
|  | AttendPWCLast | 2.622 | .472 | 30.906 | 1 | .000 | 13.763 | 5.461 | 34.686 |
|  | Number_of_live_children |  |  | 3.998 | 2 | .135 |  |  |  |
|  | Number_of_live_children(1) | -.687 | .428 | 2.570 | 1 | .109 | .503 | .217 | 1.165 |
|  | Number_of_live_children(2) | -1.235 | .768 | 2.588 | 1 | .108 | .291 | .065 | 1.310 |
|  | Constant | -29.259 | 15548.010 | .000 | 1 | .998 | .000 |  |  |
| Step 9^a^ | residence | .802 | .556 | 2.079 | 1 | .149 | 2.230 | .750 | 6.633 |
|  | time2reach | 1.567 | .512 | 9.376 | 1 | .002 | 4.792 | 1.758 | 13.065 |
|  | lastpregnw | 1.288 | .624 | 4.256 | 1 | .039 | 3.625 | 1.066 | 12.324 |
|  | toldbpcrp | 1.021 | .649 | 2.476 | 1 | .116 | 2.775 | .778 | 9.895 |
|  | partneracc | 1.359 | .410 | 10.969 | 1 | .001 | 3.893 | 1.742 | 8.703 |
|  | placedeliv |  |  | .890 | 2 | .641 |  |  |  |
|  | placedeliv(1) | 19.176 | 15601.683 | .000 | 1 | .999 | 212763646.363 | .000 | . |
|  | placedeliv(2) | 18.784 | 15601.683 | .000 | 1 | .999 | 143761278.525 | .000 | . |
|  | informed2r | 1.113 | .484 | 5.294 | 1 | .021 | 3.044 | 1.179 | 7.857 |
|  | Monthly_income |  |  | 3.284 | 2 | .194 |  |  |  |
|  | Monthly_income(1) | .298 | .662 | .203 | 1 | .652 | 1.347 | .368 | 4.933 |
|  | Monthly_income(2) | 1.025 | .626 | 2.686 | 1 | .101 | 2.788 | .818 | 9.505 |
|  | FirstANCBooking | 1.007 | .423 | 5.659 | 1 | .017 | 2.738 | 1.194 | 6.276 |
|  | BPCRPKnowledge | -.317 | .462 | .469 | 1 | .493 | .729 | .295 | 1.802 |
|  | ModeTranspoCat |  |  | 4.546 | 2 | .103 |  |  |  |
|  | ModeTranspoCat(1) | .003 | .597 | .000 | 1 | .996 | 1.003 | .311 | 3.234 |
|  | ModeTranspoCat(2) | 1.257 | .604 | 4.337 | 1 | .037 | 3.516 | 1.077 | 11.482 |
|  | EducWomenCat |  |  | 2.724 | 2 | .256 |  |  |  |
|  | EducWomenCat(1) | .236 | .639 | .136 | 1 | .712 | 1.266 | .362 | 4.429 |
|  | EducWomenCat(2) | .915 | .674 | 1.845 | 1 | .174 | 2.498 | .667 | 9.360 |
|  | EducHusbCat2 |  |  | 2.569 | 2 | .277 |  |  |  |
|  | EducHusbCat2(1) | .785 | .785 | 1.001 | 1 | .317 | 2.193 | .471 | 10.208 |
|  | EducHusbCat2(2) | 1.228 | .788 | 2.432 | 1 | .119 | 3.415 | .730 | 15.990 |
|  | AttendPWCLast | 2.709 | .456 | 35.350 | 1 | .000 | 15.018 | 6.148 | 36.684 |
|  | Number_of_live_children |  |  | 4.181 | 2 | .124 |  |  |  |
|  | Number_of_live_children(1) | -.690 | .428 | 2.597 | 1 | .107 | .501 | .217 | 1.161 |
|  | Number_of_live_children(2) | -1.277 | .762 | 2.805 | 1 | .094 | .279 | .063 | 1.243 |
|  | Constant | -29.031 | 15601.683 | .000 | 1 | .999 | .000 |  |  |
| Step 10^a^ | residence | .853 | .551 | 2.393 | 1 | .122 | 2.346 | .796 | 6.913 |
|  | time2reach | 1.575 | .513 | 9.414 | 1 | .002 | 4.829 | 1.766 | 13.206 |
|  | lastpregnw | 1.257 | .621 | 4.100 | 1 | .043 | 3.516 | 1.041 | 11.874 |
|  | toldbpcrp | .934 | .635 | 2.165 | 1 | .141 | 2.544 | .733 | 8.825 |
|  | partneracc | 1.301 | .399 | 10.641 | 1 | .001 | 3.673 | 1.681 | 8.026 |
|  | placedeliv |  |  | .806 | 2 | .668 |  |  |  |
|  | placedeliv(1) | 19.241 | 15514.230 | .000 | 1 | .999 | 227062798.015 | .000 | . |
|  | placedeliv(2) | 18.868 | 15514.230 | .000 | 1 | .999 | 156362280.459 | .000 | . |
|  | informed2r | 1.109 | .483 | 5.282 | 1 | .022 | 3.031 | 1.177 | 7.804 |
|  | Monthly_income |  |  | 3.116 | 2 | .211 |  |  |  |
|  | Monthly_income(1) | .266 | .663 | .160 | 1 | .689 | 1.304 | .356 | 4.784 |
|  | Monthly_income(2) | .990 | .627 | 2.495 | 1 | .114 | 2.691 | .788 | 9.190 |
|  | FirstANCBooking | 1.008 | .424 | 5.644 | 1 | .018 | 2.740 | 1.193 | 6.293 |
|  | ModeTranspoCat |  |  | 4.709 | 2 | .095 |  |  |  |
|  | ModeTranspoCat(1) | -.007 | .598 | .000 | 1 | .990 | .993 | .307 | 3.206 |
|  | ModeTranspoCat(2) | 1.278 | .605 | 4.467 | 1 | .035 | 3.588 | 1.097 | 11.735 |
|  | EducWomenCat |  |  | 2.661 | 2 | .264 |  |  |  |
|  | EducWomenCat(1) | .260 | .639 | .165 | 1 | .684 | 1.297 | .371 | 4.533 |
|  | EducWomenCat(2) | .920 | .674 | 1.862 | 1 | .172 | 2.509 | .669 | 9.407 |
|  | EducHusbCat2 |  |  | 2.415 | 2 | .299 |  |  |  |
|  | EducHusbCat2(1) | .787 | .784 | 1.008 | 1 | .315 | 2.196 | .473 | 10.203 |
|  | EducHusbCat2(2) | 1.192 | .784 | 2.315 | 1 | .128 | 3.295 | .709 | 15.305 |
|  | AttendPWCLast | 2.666 | .448 | 35.408 | 1 | .000 | 14.383 | 5.977 | 34.613 |
|  | Number_of_live_children |  |  | 4.201 | 2 | .122 |  |  |  |
|  | Number_of_live_children(1) | -.687 | .428 | 2.580 | 1 | .108 | .503 | .217 | 1.163 |
|  | Number_of_live_children(2) | -1.292 | .767 | 2.835 | 1 | .092 | .275 | .061 | 1.236 |
|  | Constant | -29.234 | 15514.230 | .000 | 1 | .998 | .000 |  |  |
| Step 11^a^ | residence | .712 | .528 | 1.816 | 1 | .178 | 2.038 | .724 | 5.738 |
|  | time2reach | 1.563 | .510 | 9.389 | 1 | .002 | 4.773 | 1.756 | 12.973 |
|  | lastpregnw | 1.185 | .614 | 3.726 | 1 | .054 | 3.270 | .982 | 10.892 |
|  | toldbpcrp | .858 | .629 | 1.861 | 1 | .172 | 2.358 | .688 | 8.089 |
|  | partneracc | 1.340 | .398 | 11.332 | 1 | .001 | 3.821 | 1.751 | 8.338 |
|  | informed2r | 1.123 | .478 | 5.512 | 1 | .019 | 3.073 | 1.204 | 7.847 |
|  | Monthly_income |  |  | 2.892 | 2 | .235 |  |  |  |
|  | Monthly_income(1) | .225 | .658 | .117 | 1 | .733 | 1.252 | .345 | 4.550 |
|  | Monthly_income(2) | .935 | .621 | 2.266 | 1 | .132 | 2.548 | .754 | 8.613 |
|  | FirstANCBooking | 1.006 | .423 | 5.651 | 1 | .017 | 2.735 | 1.193 | 6.270 |
|  | ModeTranspoCat |  |  | 5.300 | 2 | .071 |  |  |  |
|  | ModeTranspoCat(1) | .009 | .594 | .000 | 1 | .988 | 1.009 | .315 | 3.235 |
|  | ModeTranspoCat(2) | 1.356 | .603 | 5.057 | 1 | .025 | 3.879 | 1.190 | 12.641 |
|  | EducWomenCat |  |  | 3.161 | 2 | .206 |  |  |  |
|  | EducWomenCat(1) | .336 | .635 | .280 | 1 | .597 | 1.400 | .403 | 4.862 |
|  | EducWomenCat(2) | 1.033 | .675 | 2.343 | 1 | .126 | 2.809 | .749 | 10.539 |
|  | EducHusbCat2 |  |  | 2.566 | 2 | .277 |  |  |  |
|  | EducHusbCat2(1) | .749 | .772 | .941 | 1 | .332 | 2.115 | .465 | 9.611 |
|  | EducHusbCat2(2) | 1.205 | .776 | 2.412 | 1 | .120 | 3.337 | .729 | 15.266 |
|  | AttendPWCLast | 2.657 | .445 | 35.700 | 1 | .000 | 14.258 | 5.963 | 34.091 |
|  | Number_of_live_children |  |  | 4.017 | 2 | .134 |  |  |  |
|  | Number_of_live_children(1) | -.678 | .424 | 2.557 | 1 | .110 | .507 | .221 | 1.166 |
|  | Number_of_live_children(2) | -1.240 | .773 | 2.575 | 1 | .109 | .289 | .064 | 1.316 |
|  | Constant | -9.933 | 1.817 | 29.891 | 1 | .000 | .000 |  |  |
| Step 12^a^ | residence | .612 | .510 | 1.442 | 1 | .230 | 1.845 | .679 | 5.012 |
|  | time2reach | 1.539 | .488 | 9.930 | 1 | .002 | 4.661 | 1.789 | 12.141 |
|  | lastpregnw | 1.048 | .600 | 3.053 | 1 | .081 | 2.853 | .880 | 9.250 |
|  | toldbpcrp | .915 | .620 | 2.176 | 1 | .140 | 2.497 | .740 | 8.423 |
|  | partneracc | 1.250 | .388 | 10.362 | 1 | .001 | 3.491 | 1.631 | 7.474 |
|  | informed2r | 1.050 | .468 | 5.032 | 1 | .025 | 2.858 | 1.142 | 7.156 |
|  | Monthly_income |  |  | 3.763 | 2 | .152 |  |  |  |
|  | Monthly_income(1) | .272 | .652 | .175 | 1 | .676 | 1.313 | .366 | 4.714 |
|  | Monthly_income(2) | 1.053 | .605 | 3.032 | 1 | .082 | 2.866 | .876 | 9.375 |
|  | FirstANCBooking | 1.020 | .412 | 6.148 | 1 | .013 | 2.775 | 1.238 | 6.216 |
|  | ModeTranspoCat |  |  | 5.389 | 2 | .068 |  |  |  |
|  | ModeTranspoCat(1) | -.143 | .586 | .060 | 1 | .807 | .867 | .275 | 2.733 |
|  | ModeTranspoCat(2) | 1.312 | .597 | 4.836 | 1 | .028 | 3.714 | 1.153 | 11.961 |
|  | EducWomenCat |  |  | 6.087 | 2 | .048 |  |  |  |
|  | EducWomenCat(1) | .572 | .635 | .812 | 1 | .368 | 1.771 | .511 | 6.143 |
|  | EducWomenCat(2) | 1.403 | .642 | 4.775 | 1 | .029 | 4.069 | 1.156 | 14.328 |
|  | AttendPWCLast | 2.636 | .435 | 36.699 | 1 | .000 | 13.961 | 5.950 | 32.760 |
|  | Number_of_live_children |  |  | 4.497 | 2 | .106 |  |  |  |
|  | Number_of_live_children(1) | -.657 | .419 | 2.462 | 1 | .117 | .518 | .228 | 1.178 |
|  | Number_of_live_children(2) | -1.398 | .770 | 3.293 | 1 | .070 | .247 | .055 | 1.118 |
|  | Constant | -8.966 | 1.572 | 32.541 | 1 | .000 | .000 |  |  |
| Step 13^a^ | time2reach | 1.336 | .454 | 8.649 | 1 | .003 | 3.803 | 1.561 | 9.264 |
|  | lastpregnw | 1.139 | .600 | 3.600 | 1 | .058 | 3.125 | .963 | 10.137 |
|  | toldbpcrp | .990 | .618 | 2.564 | 1 | .109 | 2.692 | .801 | 9.043 |
|  | partneracc | 1.190 | .383 | 9.677 | 1 | .002 | 3.288 | 1.553 | 6.961 |
|  | informed2r | 1.112 | .468 | 5.640 | 1 | .018 | 3.042 | 1.214 | 7.617 |
|  | Monthly_income |  |  | 2.686 | 2 | .261 |  |  |  |
|  | Monthly_income(1) | .232 | .649 | .128 | 1 | .720 | 1.262 | .353 | 4.504 |
|  | Monthly_income(2) | .837 | .570 | 2.157 | 1 | .142 | 2.310 | .756 | 7.060 |
|  | FirstANCBooking | .967 | .408 | 5.611 | 1 | .018 | 2.629 | 1.182 | 5.851 |
|  | ModeTranspoCat |  |  | 5.637 | 2 | .060 |  |  |  |
|  | ModeTranspoCat(1) | -.218 | .577 | .143 | 1 | .705 | .804 | .260 | 2.489 |
|  | ModeTranspoCat(2) | 1.313 | .596 | 4.861 | 1 | .027 | 3.718 | 1.157 | 11.949 |
|  | EducWomenCat |  |  | 5.383 | 2 | .068 |  |  |  |
|  | EducWomenCat(1) | .572 | .629 | .827 | 1 | .363 | 1.772 | .516 | 6.084 |
|  | EducWomenCat(2) | 1.326 | .637 | 4.337 | 1 | .037 | 3.765 | 1.081 | 13.107 |
|  | AttendPWCLast | 2.703 | .435 | 38.620 | 1 | .000 | 14.927 | 6.364 | 35.011 |
|  | Number_of_live_children |  |  | 4.206 | 2 | .122 |  |  |  |
|  | Number_of_live_children(1) | -.578 | .411 | 1.976 | 1 | .160 | .561 | .251 | 1.256 |
|  | Number_of_live_children(2) | -1.381 | .753 | 3.368 | 1 | .066 | .251 | .057 | 1.099 |
|  | Constant | -7.849 | 1.227 | 40.901 | 1 | .000 | .000 |  |  |
| Step 14^a^ | time2reach | 1.346 | .451 | 8.901 | 1 | .003 | 3.843 | 1.587 | 9.305 |
|  | lastpregnw | 1.096 | .592 | 3.431 | 1 | .064 | 2.993 | .938 | 9.545 |
|  | toldbpcrp | .943 | .612 | 2.370 | 1 | .124 | 2.566 | .773 | 8.521 |
|  | partneracc | 1.165 | .376 | 9.599 | 1 | .002 | 3.206 | 1.534 | 6.699 |
|  | informed2r | 1.158 | .461 | 6.312 | 1 | .012 | 3.184 | 1.290 | 7.860 |
|  | FirstANCBooking | 1.172 | .391 | 8.971 | 1 | .003 | 3.227 | 1.499 | 6.948 |
|  | ModeTranspoCat |  |  | 5.234 | 2 | .073 |  |  |  |
|  | ModeTranspoCat(1) | -.152 | .574 | .070 | 1 | .791 | .859 | .279 | 2.647 |
|  | ModeTranspoCat(2) | 1.208 | .569 | 4.498 | 1 | .034 | 3.345 | 1.096 | 10.213 |
|  | EducWomenCat |  |  | 6.974 | 2 | .031 |  |  |  |
|  | EducWomenCat(1) | .704 | .614 | 1.317 | 1 | .251 | 2.022 | .607 | 6.733 |
|  | EducWomenCat(2) | 1.508 | .618 | 5.947 | 1 | .015 | 4.517 | 1.344 | 15.178 |
|  | AttendPWCLast | 2.564 | .417 | 37.859 | 1 | .000 | 12.993 | 5.740 | 29.407 |
|  | Number_of_live_children |  |  | 5.049 | 2 | .080 |  |  |  |
|  | Number_of_live_children(1) | -.517 | .405 | 1.634 | 1 | .201 | .596 | .270 | 1.317 |
|  | Number_of_live_children(2) | -1.613 | .750 | 4.627 | 1 | .031 | .199 | .046 | .867 |
|  | Constant | -7.405 | 1.108 | 44.625 | 1 | .000 | .001 |  |  |
| Step 15^a^ | time2reach | 1.385 | .446 | 9.632 | 1 | .002 | 3.995 | 1.666 | 9.580 |
|  | lastpregnw | 1.190 | .596 | 3.987 | 1 | .046 | 3.287 | 1.022 | 10.569 |
|  | partneracc | 1.293 | .370 | 12.187 | 1 | .000 | 3.642 | 1.763 | 7.526 |
|  | informed2r | 1.274 | .454 | 7.869 | 1 | .005 | 3.574 | 1.468 | 8.701 |
|  | FirstANCBooking | 1.193 | .388 | 9.458 | 1 | .002 | 3.295 | 1.541 | 7.047 |
|  | ModeTranspoCat |  |  | 5.920 | 2 | .052 |  |  |  |
|  | ModeTranspoCat(1) | -.068 | .570 | .014 | 1 | .905 | .934 | .306 | 2.854 |
|  | ModeTranspoCat(2) | 1.303 | .561 | 5.388 | 1 | .020 | 3.680 | 1.225 | 11.058 |
|  | EducWomenCat |  |  | 6.885 | 2 | .032 |  |  |  |
|  | EducWomenCat(1) | .584 | .610 | .916 | 1 | .339 | 1.793 | .542 | 5.929 |
|  | EducWomenCat(2) | 1.434 | .614 | 5.455 | 1 | .020 | 4.194 | 1.259 | 13.969 |
|  | AttendPWCLast | 2.636 | .412 | 40.909 | 1 | .000 | 13.956 | 6.223 | 31.302 |
|  | Number_of_live_children |  |  | 5.036 | 2 | .081 |  |  |  |
|  | Number_of_live_children(1) | -.561 | .400 | 1.965 | 1 | .161 | .571 | .261 | 1.250 |
|  | Number_of_live_children(2) | -1.573 | .749 | 4.409 | 1 | .036 | .207 | .048 | .901 |
|  | Constant | -6.827 | 1.015 | 45.209 | 1 | .000 | .001 |  |  |
| a. Variable(s) entered on step 1: residence, time2reach, when2begin, culturevsp, lastpregnw, toldpregnc, toldbpcrp, partneracc, placedeliv, be4dischar, informed2r, Monthly_income, AgePregCat, FirstANCBooking, BPCRPKnowledge, ModeTranspoCat, EducWomenCat, EducHusbCat2, AttendPWCLast, OccupWomLast, OccupHusbcatt, ReceivedadequateANCServices, Number_of_live_children. | | | | | | | | | |

| Correlation Matrix |  |  |  |  |  |  |  |  |  |  |  |  |  |  |  |  |  |  |  |  |  |  |  |  |  |  |  |  |  |  |  |  |  |  |  |  |  |  |  |  |  |  |  |  |  |  |  |  |  |  |  |  |  |  |  |  |
| --- | --- | --- | --- | --- | --- | --- | --- | --- | --- | --- | --- | --- | --- | --- | --- | --- | --- | --- | --- | --- | --- | --- | --- | --- | --- | --- | --- | --- | --- | --- | --- | --- | --- | --- | --- | --- | --- | --- | --- | --- | --- | --- | --- | --- | --- | --- | --- | --- | --- | --- | --- | --- | --- | --- | --- | --- |
|  | | | Constant | residence | time2reach | when2begin | culturevsp | lastpregnw | toldpregnc | toldbpcrp | partneracc | placedeliv(1) | placedeliv(2) | be4dischar | informed2r | Monthly_income(1) | Monthly_income(2) | AgePregCat(1) | FirstANCBooking | BPCRPKnowledge | ModeTranspoCat(1) | ModeTranspoCat(2) | EducWomenCat(1) | EducWomenCat(2) | EducHusbCat2(1) | EducHusbCat2(2) | AttendPWCLast | OccupWomLast | OccupHusbcatt | ReceivedadequateANCServices | Number_of_live_children(1) | Number_of_live_children(2) | residence | time2reach | when2begin | lastpregnw | toldbpcrp | partneracc | placedeliv(1) | placedeliv(2) | be4dischar | informed2r | Monthly_income(1) | Monthly_income(2) | AgePregCat(1) | FirstANCBooking | BPCRPKnowledge | ModeTranspoCat(1) | ModeTranspoCat(2) | EducWomenCat(1) | EducWomenCat(2) | EducHusbCat2(1) | EducHusbCat2(2) | AttendPWCLast | Number_of_live_children(1) | Number_of_live_children(2) |
| Step 1 | | Constant | 1.000 | .000 | .000 | .000 | .000 | .000 | .000 | .000 | .000 | -1.000 | -1.000 | .000 | .000 | .000 | .000 | .000 | .000 | .000 | .000 | .000 | .000 | .000 | .000 | .000 | .000 | .000 | .000 | .000 | .000 | .000 |  |  |  |  |  |  |  |  |  |  |  |  |  |  |  |  |  |  |  |  |  |  |  |  |
|  |  | residence | .000 | 1.000 | .319 | .115 | .006 | -.028 | .054 | -.067 | .066 | .000 | .000 | .049 | -.133 | .071 | .225 | -.125 | .088 | .131 | .131 | -.038 | .016 | .040 | .102 | .069 | -.073 | .145 | .205 | -.031 | -.209 | -.061 |  |  |  |  |  |  |  |  |  |  |  |  |  |  |  |  |  |  |  |  |  |  |  |  |
|  |  | time2reach | .000 | .319 | 1.000 | .083 | .006 | .130 | .068 | -.039 | .087 | .000 | .000 | -.209 | .072 | -.027 | .053 | -.028 | .092 | .029 | .156 | .339 | -.021 | -.104 | .272 | .176 | .121 | .061 | -.131 | -.006 | -.044 | -.145 |  |  |  |  |  |  |  |  |  |  |  |  |  |  |  |  |  |  |  |  |  |  |  |  |
|  |  | when2begin | .000 | .115 | .083 | 1.000 | -.119 | .063 | .111 | .016 | .202 | .000 | .000 | .076 | -.081 | -.164 | -.158 | -.025 | .113 | .119 | -.125 | -.001 | -.034 | -.008 | .049 | .116 | .088 | -.005 | -.045 | -.183 | .037 | -.074 |  |  |  |  |  |  |  |  |  |  |  |  |  |  |  |  |  |  |  |  |  |  |  |  |
|  |  | culturevsp | .000 | .006 | .006 | -.119 | 1.000 | -.129 | .027 | .001 | -.231 | .000 | .000 | -.172 | -.019 | -.004 | .097 | -.074 | -.072 | .039 | .039 | -.086 | .050 | .058 | .044 | -.030 | -.156 | -.035 | .018 | -.032 | -.234 | .032 |  |  |  |  |  |  |  |  |  |  |  |  |  |  |  |  |  |  |  |  |  |  |  |  |
|  |  | lastpregnw | .000 | -.028 | .130 | .063 | -.129 | 1.000 | .071 | -.059 | .145 | .000 | .000 | -.005 | .090 | .048 | .041 | -.081 | .016 | -.079 | .032 | .038 | -.120 | -.051 | .108 | .139 | .117 | -.012 | -.001 | .004 | .104 | .062 |  |  |  |  |  |  |  |  |  |  |  |  |  |  |  |  |  |  |  |  |  |  |  |  |
|  |  | toldpregnc | .000 | .054 | .068 | .111 | .027 | .071 | 1.000 | -.421 | .169 | .000 | .000 | -.017 | -.055 | -.006 | .050 | .020 | .184 | -.112 | .073 | -.112 | .045 | .061 | .169 | .096 | -.012 | -.081 | .014 | -.154 | -.001 | -.027 |  |  |  |  |  |  |  |  |  |  |  |  |  |  |  |  |  |  |  |  |  |  |  |  |
|  |  | toldbpcrp | .000 | -.067 | -.039 | .016 | .001 | -.059 | -.421 | 1.000 | -.115 | .000 | .000 | .047 | -.067 | .069 | .014 | .044 | -.085 | -.063 | -.117 | -.052 | .091 | .071 | -.045 | -.051 | .007 | .009 | .052 | -.142 | .017 | -.041 |  |  |  |  |  |  |  |  |  |  |  |  |  |  |  |  |  |  |  |  |  |  |  |  |
|  |  | partneracc | .000 | .066 | .087 | .202 | -.231 | .145 | .169 | -.115 | 1.000 | .000 | .000 | .140 | .042 | .108 | .121 | .160 | .061 | -.219 | .033 | -.052 | -.175 | -.028 | .117 | .229 | .122 | -.157 | -.065 | -.132 | .003 | .020 |  |  |  |  |  |  |  |  |  |  |  |  |  |  |  |  |  |  |  |  |  |  |  |  |
|  |  | placedeliv(1) | -1.000 | .000 | .000 | .000 | .000 | .000 | .000 | .000 | .000 | 1.000 | 1.000 | .000 | .000 | .000 | .000 | .000 | .000 | .000 | .000 | .000 | .000 | .000 | .000 | .000 | .000 | .000 | .000 | .000 | .000 | .000 |  |  |  |  |  |  |  |  |  |  |  |  |  |  |  |  |  |  |  |  |  |  |  |  |
|  |  | placedeliv(2) | -1.000 | .000 | .000 | .000 | .000 | .000 | .000 | .000 | .000 | 1.000 | 1.000 | .000 | .000 | .000 | .000 | .000 | .000 | .000 | .000 | .000 | .000 | .000 | .000 | .000 | .000 | .000 | .000 | .000 | .000 | .000 |  |  |  |  |  |  |  |  |  |  |  |  |  |  |  |  |  |  |  |  |  |  |  |  |
|  |  | be4dischar | .000 | .049 | -.209 | .076 | -.172 | -.005 | -.017 | .047 | .140 | .000 | .000 | 1.000 | -.272 | .076 | .089 | -.015 | .166 | -.071 | -.056 | -.164 | .048 | .134 | -.015 | -.016 | -.201 | .050 | .113 | -.162 | .033 | .061 |  |  |  |  |  |  |  |  |  |  |  |  |  |  |  |  |  |  |  |  |  |  |  |  |
|  |  | informed2r | .000 | -.133 | .072 | -.081 | -.019 | .090 | -.055 | -.067 | .042 | .000 | .000 | -.272 | 1.000 | -.048 | -.118 | .009 | -.148 | .000 | .142 | .059 | -.117 | -.132 | .014 | .088 | .179 | .025 | -.050 | .006 | .036 | -.063 |  |  |  |  |  |  |  |  |  |  |  |  |  |  |  |  |  |  |  |  |  |  |  |  |
|  |  | Monthly_income(1) | .000 | .071 | -.027 | -.164 | -.004 | .048 | -.006 | .069 | .108 | .000 | .000 | .076 | -.048 | 1.000 | .642 | -.106 | -.042 | -.093 | .104 | -.187 | -.031 | .038 | .051 | -.011 | -.024 | .051 | .002 | .102 | -.107 | .031 |  |  |  |  |  |  |  |  |  |  |  |  |  |  |  |  |  |  |  |  |  |  |  |  |
|  |  | Monthly_income(2) | .000 | .225 | .053 | -.158 | .097 | .041 | .050 | .014 | .121 | .000 | .000 | .089 | -.118 | .642 | 1.000 | -.098 | -.136 | -.135 | .026 | -.017 | -.072 | .012 | .011 | -.025 | .120 | -.034 | -.062 | .052 | -.159 | .111 |  |  |  |  |  |  |  |  |  |  |  |  |  |  |  |  |  |  |  |  |  |  |  |  |
|  |  | AgePregCat(1) | .000 | -.125 | -.028 | -.025 | -.074 | -.081 | .020 | .044 | .160 | .000 | .000 | -.015 | .009 | -.106 | -.098 | 1.000 | .143 | -.046 | -.119 | .045 | -.109 | -.115 | .009 | .093 | -.089 | -.273 | -.009 | .038 | .007 | -.082 |  |  |  |  |  |  |  |  |  |  |  |  |  |  |  |  |  |  |  |  |  |  |  |  |
|  |  | FirstANCBooking | .000 | .088 | .092 | .113 | -.072 | .016 | .184 | -.085 | .061 | .000 | .000 | .166 | -.148 | -.042 | -.136 | .143 | 1.000 | -.021 | -.188 | -.209 | .055 | .115 | .211 | .132 | -.161 | -.009 | -.109 | -.091 | -.051 | -.162 |  |  |  |  |  |  |  |  |  |  |  |  |  |  |  |  |  |  |  |  |  |  |  |  |
|  |  | BPCRPKnowledge | .000 | .131 | .029 | .119 | .039 | -.079 | -.112 | -.063 | -.219 | .000 | .000 | -.071 | .000 | -.093 | -.135 | -.046 | -.021 | 1.000 | -.059 | .043 | .059 | -.011 | .002 | -.044 | -.130 | .062 | -.031 | -.181 | -.004 | -.058 |  |  |  |  |  |  |  |  |  |  |  |  |  |  |  |  |  |  |  |  |  |  |  |  |
|  |  | ModeTranspoCat(1) | .000 | .131 | .156 | -.125 | .039 | .032 | .073 | -.117 | .033 | .000 | .000 | -.056 | .142 | .104 | .026 | -.119 | -.188 | -.059 | 1.000 | .193 | -.051 | -.070 | .060 | .098 | .014 | .101 | .019 | .084 | -.104 | .091 |  |  |  |  |  |  |  |  |  |  |  |  |  |  |  |  |  |  |  |  |  |  |  |  |
|  |  | ModeTranspoCat(2) | .000 | -.038 | .339 | -.001 | -.086 | .038 | -.112 | -.052 | -.052 | .000 | .000 | -.164 | .059 | -.187 | -.017 | .045 | -.209 | .043 | .193 | 1.000 | -.038 | -.065 | -.033 | .067 | .131 | .007 | -.110 | .111 | .065 | .018 |  |  |  |  |  |  |  |  |  |  |  |  |  |  |  |  |  |  |  |  |  |  |  |  |
|  |  | EducWomenCat(1) | .000 | .016 | -.021 | -.034 | .050 | -.120 | .045 | .091 | -.175 | .000 | .000 | .048 | -.117 | -.031 | -.072 | -.109 | .055 | .059 | -.051 | -.038 | 1.000 | .716 | -.135 | -.219 | .028 | .028 | .065 | -.132 | -.023 | -.100 |  |  |  |  |  |  |  |  |  |  |  |  |  |  |  |  |  |  |  |  |  |  |  |  |
|  |  | EducWomenCat(2) | .000 | .040 | -.104 | -.008 | .058 | -.051 | .061 | .071 | -.028 | .000 | .000 | .134 | -.132 | .038 | .012 | -.115 | .115 | -.011 | -.070 | -.065 | .716 | 1.000 | -.074 | -.280 | .082 | -.190 | .151 | -.160 | .088 | .002 |  |  |  |  |  |  |  |  |  |  |  |  |  |  |  |  |  |  |  |  |  |  |  |  |
|  |  | EducHusbCat2(1) | .000 | .102 | .272 | .049 | .044 | .108 | .169 | -.045 | .117 | .000 | .000 | -.015 | .014 | .051 | .011 | .009 | .211 | .002 | .060 | -.033 | -.135 | -.074 | 1.000 | .755 | -.027 | .005 | -.026 | -.114 | -.028 | .026 |  |  |  |  |  |  |  |  |  |  |  |  |  |  |  |  |  |  |  |  |  |  |  |  |
|  |  | EducHusbCat2(2) | .000 | .069 | .176 | .116 | -.030 | .139 | .096 | -.051 | .229 | .000 | .000 | -.016 | .088 | -.011 | -.025 | .093 | .132 | -.044 | .098 | .067 | -.219 | -.280 | .755 | 1.000 | .082 | .030 | -.271 | -.130 | -.038 | .039 |  |  |  |  |  |  |  |  |  |  |  |  |  |  |  |  |  |  |  |  |  |  |  |  |
|  |  | AttendPWCLast | .000 | -.073 | .121 | .088 | -.156 | .117 | -.012 | .007 | .122 | .000 | .000 | -.201 | .179 | -.024 | .120 | -.089 | -.161 | -.130 | .014 | .131 | .028 | .082 | -.027 | .082 | 1.000 | -.064 | -.003 | -.058 | -.149 | -.169 |  |  |  |  |  |  |  |  |  |  |  |  |  |  |  |  |  |  |  |  |  |  |  |  |
|  |  | OccupWomLast | .000 | .145 | .061 | -.005 | -.035 | -.012 | -.081 | .009 | -.157 | .000 | .000 | .050 | .025 | .051 | -.034 | -.273 | -.009 | .062 | .101 | .007 | .028 | -.190 | .005 | .030 | -.064 | 1.000 | -.346 | .125 | -.193 | -.067 |  |  |  |  |  |  |  |  |  |  |  |  |  |  |  |  |  |  |  |  |  |  |  |  |
|  |  | OccupHusbcatt | .000 | .205 | -.131 | -.045 | .018 | -.001 | .014 | .052 | -.065 | .000 | .000 | .113 | -.050 | .002 | -.062 | -.009 | -.109 | -.031 | .019 | -.110 | .065 | .151 | -.026 | -.271 | -.003 | -.346 | 1.000 | -.037 | .049 | .094 |  |  |  |  |  |  |  |  |  |  |  |  |  |  |  |  |  |  |  |  |  |  |  |  |
|  |  | ReceivedadequateANCServices | .000 | -.031 | -.006 | -.183 | -.032 | .004 | -.154 | -.142 | -.132 | .000 | .000 | -.162 | .006 | .102 | .052 | .038 | -.091 | -.181 | .084 | .111 | -.132 | -.160 | -.114 | -.130 | -.058 | .125 | -.037 | 1.000 | .023 | .046 |  |  |  |  |  |  |  |  |  |  |  |  |  |  |  |  |  |  |  |  |  |  |  |  |
|  |  | Number_of_live_children(1) | .000 | -.209 | -.044 | .037 | -.234 | .104 | -.001 | .017 | .003 | .000 | .000 | .033 | .036 | -.107 | -.159 | .007 | -.051 | -.004 | -.104 | .065 | -.023 | .088 | -.028 | -.038 | -.149 | -.193 | .049 | .023 | 1.000 | .289 |  |  |  |  |  |  |  |  |  |  |  |  |  |  |  |  |  |  |  |  |  |  |  |  |
|  |  | Number_of_live_children(2) | .000 | -.061 | -.145 | -.074 | .032 | .062 | -.027 | -.041 | .020 | .000 | .000 | .061 | -.063 | .031 | .111 | -.082 | -.162 | -.058 | .091 | .018 | -.100 | .002 | .026 | .039 | -.169 | -.067 | .094 | .046 | .289 | 1.000 |  |  |  |  |  |  |  |  |  |  |  |  |  |  |  |  |  |  |  |  |  |  |  |  |
| Step 2 | | Constant | 1.000 | .000 | .000 | .000 |  | .000 | .000 | .000 | .000 | -1.000 | -1.000 | .000 | .000 | .000 | .000 | .000 | .000 | .000 | .000 | .000 | .000 | .000 | .000 | .000 | .000 | .000 | .000 | .000 | .000 | .000 |  |  |  |  |  |  |  |  |  |  |  |  |  |  |  |  |  |  |  |  |  |  |  |  |
|  |  | residence | .000 | 1.000 | .318 | .116 |  | -.028 | .054 | -.068 | .070 | .000 | .000 | .052 | -.133 | .071 | .226 | -.126 | .089 | .131 | .131 | -.038 | .015 | .040 | .101 | .069 | -.073 | .146 | .205 | -.031 | -.213 | -.061 |  |  |  |  |  |  |  |  |  |  |  |  |  |  |  |  |  |  |  |  |  |  |  |  |
|  |  | time2reach | .000 | .318 | 1.000 | .084 |  | .132 | .068 | -.040 | .091 | .000 | .000 | -.211 | .072 | -.027 | .053 | -.028 | .092 | .030 | .155 | .339 | -.021 | -.104 | .273 | .176 | .124 | .062 | -.132 | -.006 | -.044 | -.146 |  |  |  |  |  |  |  |  |  |  |  |  |  |  |  |  |  |  |  |  |  |  |  |  |
|  |  | when2begin | .000 | .116 | .084 | 1.000 |  | .048 | .115 | .016 | .181 | .000 | .000 | .058 | -.084 | -.165 | -.148 | -.034 | .107 | .125 | -.122 | -.012 | -.028 | .000 | .055 | .112 | .070 | -.009 | -.044 | -.188 | .011 | -.071 |  |  |  |  |  |  |  |  |  |  |  |  |  |  |  |  |  |  |  |  |  |  |  |  |
|  |  | lastpregnw | .000 | -.028 | .132 | .048 |  | 1.000 | .075 | -.060 | .120 | .000 | .000 | -.028 | .089 | .047 | .053 | -.091 | .007 | -.074 | .037 | .027 | -.114 | -.044 | .115 | .137 | .100 | -.016 | .001 | .000 | .077 | .067 |  |  |  |  |  |  |  |  |  |  |  |  |  |  |  |  |  |  |  |  |  |  |  |  |
|  |  | toldpregnc | .000 | .054 | .068 | .115 |  | .075 | 1.000 | -.422 | .180 | .000 | .000 | -.012 | -.054 | -.005 | .048 | .021 | .186 | -.112 | .073 | -.111 | .044 | .059 | .169 | .097 | -.008 | -.079 | .014 | -.153 | .006 | -.028 |  |  |  |  |  |  |  |  |  |  |  |  |  |  |  |  |  |  |  |  |  |  |  |  |
|  |  | toldbpcrp | .000 | -.068 | -.040 | .016 |  | -.060 | -.422 | 1.000 | -.118 | .000 | .000 | .047 | -.066 | .069 | .014 | .045 | -.085 | -.064 | -.118 | -.052 | .091 | .070 | -.045 | -.050 | .008 | .008 | .051 | -.143 | .018 | -.042 |  |  |  |  |  |  |  |  |  |  |  |  |  |  |  |  |  |  |  |  |  |  |  |  |
|  |  | partneracc | .000 | .070 | .091 | .181 |  | .120 | .180 | -.118 | 1.000 | .000 | .000 | .105 | .039 | .111 | .149 | .147 | .046 | -.216 | .043 | -.074 | -.167 | -.013 | .130 | .228 | .089 | -.171 | -.062 | -.144 | -.054 | .028 |  |  |  |  |  |  |  |  |  |  |  |  |  |  |  |  |  |  |  |  |  |  |  |  |
|  |  | placedeliv(1) | -1.000 | .000 | .000 | .000 |  | .000 | .000 | .000 | .000 | 1.000 | 1.000 | .000 | .000 | .000 | .000 | .000 | .000 | .000 | .000 | .000 | .000 | .000 | .000 | .000 | .000 | .000 | .000 | .000 | .000 | .000 |  |  |  |  |  |  |  |  |  |  |  |  |  |  |  |  |  |  |  |  |  |  |  |  |
|  |  | placedeliv(2) | -1.000 | .000 | .000 | .000 |  | .000 | .000 | .000 | .000 | 1.000 | 1.000 | .000 | .000 | .000 | .000 | .000 | .000 | .000 | .000 | .000 | .000 | .000 | .000 | .000 | .000 | .000 | .000 | .000 | .000 | .000 |  |  |  |  |  |  |  |  |  |  |  |  |  |  |  |  |  |  |  |  |  |  |  |  |
|  |  | be4dischar | .000 | .052 | -.211 | .058 |  | -.028 | -.012 | .047 | .105 | .000 | .000 | 1.000 | -.280 | .077 | .108 | -.028 | .157 | -.065 | -.049 | -.182 | .056 | .146 | -.007 | -.021 | -.235 | .044 | .117 | -.171 | -.008 | .067 |  |  |  |  |  |  |  |  |  |  |  |  |  |  |  |  |  |  |  |  |  |  |  |  |
|  |  | informed2r | .000 | -.133 | .072 | -.084 |  | .089 | -.054 | -.066 | .039 | .000 | .000 | -.280 | 1.000 | -.048 | -.116 | .007 | -.150 | .001 | .143 | .058 | -.115 | -.130 | .014 | .087 | .178 | .024 | -.050 | .005 | .032 | -.063 |  |  |  |  |  |  |  |  |  |  |  |  |  |  |  |  |  |  |  |  |  |  |  |  |
|  |  | Monthly_income(1) | .000 | .071 | -.027 | -.165 |  | .047 | -.005 | .069 | .111 | .000 | .000 | .077 | -.048 | 1.000 | .646 | -.106 | -.042 | -.094 | .105 | -.188 | -.031 | .039 | .051 | -.011 | -.025 | .051 | .002 | .102 | -.111 | .030 |  |  |  |  |  |  |  |  |  |  |  |  |  |  |  |  |  |  |  |  |  |  |  |  |
|  |  | Monthly_income(2) | .000 | .226 | .053 | -.148 |  | .053 | .048 | .014 | .149 | .000 | .000 | .108 | -.116 | .646 | 1.000 | -.092 | -.130 | -.140 | .023 | -.009 | -.078 | .006 | .007 | -.023 | .138 | -.031 | -.064 | .055 | -.142 | .108 |  |  |  |  |  |  |  |  |  |  |  |  |  |  |  |  |  |  |  |  |  |  |  |  |
|  |  | AgePregCat(1) | .000 | -.126 | -.028 | -.034 |  | -.091 | .021 | .045 | .147 | .000 | .000 | -.028 | .007 | -.106 | -.092 | 1.000 | .139 | -.044 | -.117 | .039 | -.106 | -.111 | .012 | .091 | -.103 | -.278 | -.008 | .036 | -.011 | -.080 |  |  |  |  |  |  |  |  |  |  |  |  |  |  |  |  |  |  |  |  |  |  |  |  |
|  |  | FirstANCBooking | .000 | .089 | .092 | .107 |  | .007 | .186 | -.085 | .046 | .000 | .000 | .157 | -.150 | -.042 | -.130 | .139 | 1.000 | -.018 | -.185 | -.217 | .058 | .119 | .216 | .131 | -.175 | -.012 | -.107 | -.094 | -.070 | -.160 |  |  |  |  |  |  |  |  |  |  |  |  |  |  |  |  |  |  |  |  |  |  |  |  |
|  |  | BPCRPKnowledge | .000 | .131 | .030 | .125 |  | -.074 | -.112 | -.064 | -.216 | .000 | .000 | -.065 | .001 | -.094 | -.140 | -.044 | -.018 | 1.000 | -.061 | .046 | .057 | -.014 | .001 | -.042 | -.125 | .064 | -.031 | -.181 | .005 | -.060 |  |  |  |  |  |  |  |  |  |  |  |  |  |  |  |  |  |  |  |  |  |  |  |  |
|  |  | ModeTranspoCat(1) | .000 | .131 | .155 | -.122 |  | .037 | .073 | -.118 | .043 | .000 | .000 | -.049 | .143 | .105 | .023 | -.117 | -.185 | -.061 | 1.000 | .197 | -.053 | -.072 | .059 | .099 | .020 | .103 | .018 | .086 | -.098 | .090 |  |  |  |  |  |  |  |  |  |  |  |  |  |  |  |  |  |  |  |  |  |  |  |  |
|  |  | ModeTranspoCat(2) | .000 | -.038 | .339 | -.012 |  | .027 | -.111 | -.052 | -.074 | .000 | .000 | -.182 | .058 | -.188 | -.009 | .039 | -.217 | .046 | .197 | 1.000 | -.034 | -.060 | -.030 | .065 | .119 | .004 | -.109 | .109 | .046 | .021 |  |  |  |  |  |  |  |  |  |  |  |  |  |  |  |  |  |  |  |  |  |  |  |  |
|  |  | EducWomenCat(1) | .000 | .015 | -.021 | -.028 |  | -.114 | .044 | .091 | -.167 | .000 | .000 | .056 | -.115 | -.031 | -.078 | -.106 | .058 | .057 | -.053 | -.034 | 1.000 | .715 | -.137 | -.219 | .037 | .030 | .064 | -.130 | -.011 | -.102 |  |  |  |  |  |  |  |  |  |  |  |  |  |  |  |  |  |  |  |  |  |  |  |  |
|  |  | EducWomenCat(2) | .000 | .040 | -.104 | .000 |  | -.044 | .059 | .070 | -.013 | .000 | .000 | .146 | -.130 | .039 | .006 | -.111 | .119 | -.014 | -.072 | -.060 | .715 | 1.000 | -.076 | -.279 | .092 | -.188 | .149 | -.158 | .105 | -.001 |  |  |  |  |  |  |  |  |  |  |  |  |  |  |  |  |  |  |  |  |  |  |  |  |
|  |  | EducHusbCat2(1) | .000 | .101 | .273 | .055 |  | .115 | .169 | -.045 | .130 | .000 | .000 | -.007 | .014 | .051 | .007 | .012 | .216 | .001 | .059 | -.030 | -.137 | -.076 | 1.000 | .756 | -.021 | .006 | -.026 | -.114 | -.018 | .024 |  |  |  |  |  |  |  |  |  |  |  |  |  |  |  |  |  |  |  |  |  |  |  |  |
|  |  | EducHusbCat2(2) | .000 | .069 | .176 | .112 |  | .137 | .097 | -.050 | .228 | .000 | .000 | -.021 | .087 | -.011 | -.023 | .091 | .131 | -.042 | .099 | .065 | -.219 | -.279 | .756 | 1.000 | .078 | .028 | -.271 | -.132 | -.046 | .041 |  |  |  |  |  |  |  |  |  |  |  |  |  |  |  |  |  |  |  |  |  |  |  |  |
|  |  | AttendPWCLast | .000 | -.073 | .124 | .070 |  | .100 | -.008 | .008 | .089 | .000 | .000 | -.235 | .178 | -.025 | .138 | -.103 | -.175 | -.125 | .020 | .119 | .037 | .092 | -.021 | .078 | 1.000 | -.070 | .000 | -.062 | -.193 | -.167 |  |  |  |  |  |  |  |  |  |  |  |  |  |  |  |  |  |  |  |  |  |  |  |  |
|  |  | OccupWomLast | .000 | .146 | .062 | -.009 |  | -.016 | -.079 | .008 | -.171 | .000 | .000 | .044 | .024 | .051 | -.031 | -.278 | -.012 | .064 | .103 | .004 | .030 | -.188 | .006 | .028 | -.070 | 1.000 | -.345 | .124 | -.206 | -.066 |  |  |  |  |  |  |  |  |  |  |  |  |  |  |  |  |  |  |  |  |  |  |  |  |
|  |  | OccupHusbcatt | .000 | .205 | -.132 | -.044 |  | .001 | .014 | .051 | -.062 | .000 | .000 | .117 | -.050 | .002 | -.064 | -.008 | -.107 | -.031 | .018 | -.109 | .064 | .149 | -.026 | -.271 | .000 | -.345 | 1.000 | -.037 | .054 | .093 |  |  |  |  |  |  |  |  |  |  |  |  |  |  |  |  |  |  |  |  |  |  |  |  |
|  |  | ReceivedadequateANCServices | .000 | -.031 | -.006 | -.188 |  | .000 | -.153 | -.143 | -.144 | .000 | .000 | -.171 | .005 | .102 | .055 | .036 | -.094 | -.181 | .086 | .109 | -.130 | -.158 | -.114 | -.132 | -.062 | .124 | -.037 | 1.000 | .015 | .046 |  |  |  |  |  |  |  |  |  |  |  |  |  |  |  |  |  |  |  |  |  |  |  |  |
|  |  | Number_of_live_children(1) | .000 | -.213 | -.044 | .011 |  | .077 | .006 | .018 | -.054 | .000 | .000 | -.008 | .032 | -.111 | -.142 | -.011 | -.070 | .005 | -.098 | .046 | -.011 | .105 | -.018 | -.046 | -.193 | -.206 | .054 | .015 | 1.000 | .305 |  |  |  |  |  |  |  |  |  |  |  |  |  |  |  |  |  |  |  |  |  |  |  |  |
|  |  | Number_of_live_children(2) | .000 | -.061 | -.146 | -.071 |  | .067 | -.028 | -.042 | .028 | .000 | .000 | .067 | -.063 | .030 | .108 | -.080 | -.160 | -.060 | .090 | .021 | -.102 | -.001 | .024 | .041 | -.167 | -.066 | .093 | .046 | .305 | 1.000 |  |  |  |  |  |  |  |  |  |  |  |  |  |  |  |  |  |  |  |  |  |  |  |  |
| Step 3 | | Constant | 1.000 | .000 | .000 | .000 |  | .000 | .000 | .000 | .000 | -1.000 | -1.000 | .000 | .000 | .000 | .000 | .000 | .000 | .000 | .000 | .000 | .000 | .000 | .000 | .000 | .000 | .000 |  | .000 | .000 | .000 |  |  |  |  |  |  |  |  |  |  |  |  |  |  |  |  |  |  |  |  |  |  |  |  |
|  |  | residence | .000 | 1.000 | .360 | .128 |  | -.024 | .053 | -.082 | .083 | .000 | .000 | .027 | -.122 | .073 | .244 | -.125 | .114 | .142 | .138 | -.010 | .004 | .009 | .111 | .131 | -.074 | .234 |  | -.020 | -.231 | -.081 |  |  |  |  |  |  |  |  |  |  |  |  |  |  |  |  |  |  |  |  |  |  |  |  |
|  |  | time2reach | .000 | .360 | 1.000 | .073 |  | .139 | .074 | -.039 | .081 | .000 | .000 | -.198 | .064 | -.019 | .054 | -.036 | .084 | .025 | .161 | .333 | -.015 | -.088 | .273 | .150 | .129 | .017 |  | -.004 | -.042 | -.134 |  |  |  |  |  |  |  |  |  |  |  |  |  |  |  |  |  |  |  |  |  |  |  |  |
|  |  | when2begin | .000 | .128 | .073 | 1.000 |  | .054 | .116 | .014 | .178 | .000 | .000 | .069 | -.090 | -.167 | -.153 | -.034 | .104 | .120 | -.123 | -.015 | -.025 | .014 | .052 | .105 | .068 | -.035 |  | -.196 | .015 | -.067 |  |  |  |  |  |  |  |  |  |  |  |  |  |  |  |  |  |  |  |  |  |  |  |  |
|  |  | lastpregnw | .000 | -.024 | .139 | .054 |  | 1.000 | .071 | -.063 | .122 | .000 | .000 | -.026 | .081 | .047 | .055 | -.092 | .010 | -.072 | .034 | .033 | -.114 | -.047 | .115 | .146 | .098 | -.025 |  | -.002 | .076 | .066 |  |  |  |  |  |  |  |  |  |  |  |  |  |  |  |  |  |  |  |  |  |  |  |  |
|  |  | toldpregnc | .000 | .053 | .074 | .116 |  | .071 | 1.000 | -.426 | .181 | .000 | .000 | -.014 | -.057 | -.010 | .047 | .025 | .189 | -.116 | .071 | -.106 | .044 | .056 | .170 | .109 | -.007 | -.083 |  | -.154 | .003 | -.031 |  |  |  |  |  |  |  |  |  |  |  |  |  |  |  |  |  |  |  |  |  |  |  |  |
|  |  | toldbpcrp | .000 | -.082 | -.039 | .014 |  | -.063 | -.426 | 1.000 | -.118 | .000 | .000 | .041 | -.060 | .068 | .014 | .048 | -.076 | -.063 | -.123 | -.050 | .088 | .063 | -.046 | -.038 | .006 | .029 |  | -.146 | .014 | -.050 |  |  |  |  |  |  |  |  |  |  |  |  |  |  |  |  |  |  |  |  |  |  |  |  |
|  |  | partneracc | .000 | .083 | .081 | .178 |  | .122 | .181 | -.118 | 1.000 | .000 | .000 | .114 | .030 | .107 | .146 | .153 | .039 | -.220 | .043 | -.075 | -.164 | -.004 | .125 | .214 | .086 | -.204 |  | -.143 | -.051 | .036 |  |  |  |  |  |  |  |  |  |  |  |  |  |  |  |  |  |  |  |  |  |  |  |  |
|  |  | placedeliv(1) | -1.000 | .000 | .000 | .000 |  | .000 | .000 | .000 | .000 | 1.000 | 1.000 | .000 | .000 | .000 | .000 | .000 | .000 | .000 | .000 | .000 | .000 | .000 | .000 | .000 | .000 | .000 |  | .000 | .000 | .000 |  |  |  |  |  |  |  |  |  |  |  |  |  |  |  |  |  |  |  |  |  |  |  |  |
|  |  | placedeliv(2) | -1.000 | .000 | .000 | .000 |  | .000 | .000 | .000 | .000 | 1.000 | 1.000 | .000 | .000 | .000 | .000 | .000 | .000 | .000 | .000 | .000 | .000 | .000 | .000 | .000 | .000 | .000 |  | .000 | .000 | .000 |  |  |  |  |  |  |  |  |  |  |  |  |  |  |  |  |  |  |  |  |  |  |  |  |
|  |  | be4dischar | .000 | .027 | -.198 | .069 |  | -.026 | -.014 | .041 | .114 | .000 | .000 | 1.000 | -.280 | .076 | .112 | -.023 | .172 | -.061 | -.059 | -.173 | .052 | .131 | -.005 | .010 | -.240 | .092 |  | -.170 | -.011 | .051 |  |  |  |  |  |  |  |  |  |  |  |  |  |  |  |  |  |  |  |  |  |  |  |  |
|  |  | informed2r | .000 | -.122 | .064 | -.090 |  | .081 | -.057 | -.060 | .030 | .000 | .000 | -.280 | 1.000 | -.050 | -.123 | .005 | -.150 | .005 | .143 | .050 | -.109 | -.127 | .011 | .075 | .177 | .016 |  | .004 | .027 | -.062 |  |  |  |  |  |  |  |  |  |  |  |  |  |  |  |  |  |  |  |  |  |  |  |  |
|  |  | Monthly_income(1) | .000 | .073 | -.019 | -.167 |  | .047 | -.010 | .068 | .107 | .000 | .000 | .076 | -.050 | 1.000 | .652 | -.111 | -.046 | -.093 | .104 | -.182 | -.032 | .038 | .049 | -.017 | -.026 | .058 |  | .109 | -.111 | .029 |  |  |  |  |  |  |  |  |  |  |  |  |  |  |  |  |  |  |  |  |  |  |  |  |
|  |  | Monthly_income(2) | .000 | .244 | .054 | -.153 |  | .055 | .047 | .014 | .146 | .000 | .000 | .112 | -.123 | .652 | 1.000 | -.094 | -.137 | -.144 | .025 | -.009 | -.072 | .011 | .005 | -.041 | .138 | -.055 |  | .062 | -.140 | .113 |  |  |  |  |  |  |  |  |  |  |  |  |  |  |  |  |  |  |  |  |  |  |  |  |
|  |  | AgePregCat(1) | .000 | -.125 | -.036 | -.034 |  | -.092 | .025 | .048 | .153 | .000 | .000 | -.023 | .005 | -.111 | -.094 | 1.000 | .141 | -.047 | -.117 | .034 | -.108 | -.108 | .013 | .092 | -.100 | -.295 |  | .031 | -.009 | -.075 |  |  |  |  |  |  |  |  |  |  |  |  |  |  |  |  |  |  |  |  |  |  |  |  |
|  |  | FirstANCBooking | .000 | .114 | .084 | .104 |  | .010 | .189 | -.076 | .039 | .000 | .000 | .172 | -.150 | -.046 | -.137 | .141 | 1.000 | -.023 | -.186 | -.228 | .068 | .138 | .215 | .112 | -.168 | -.055 |  | -.101 | -.064 | -.154 |  |  |  |  |  |  |  |  |  |  |  |  |  |  |  |  |  |  |  |  |  |  |  |  |
|  |  | BPCRPKnowledge | .000 | .142 | .025 | .120 |  | -.072 | -.116 | -.063 | -.220 | .000 | .000 | -.061 | .005 | -.093 | -.144 | -.047 | -.023 | 1.000 | -.056 | .040 | .058 | -.007 | .000 | -.054 | -.126 | .058 |  | -.178 | .009 | -.056 |  |  |  |  |  |  |  |  |  |  |  |  |  |  |  |  |  |  |  |  |  |  |  |  |
|  |  | ModeTranspoCat(1) | .000 | .138 | .161 | -.123 |  | .034 | .071 | -.123 | .043 | .000 | .000 | -.059 | .143 | .104 | .025 | -.117 | -.186 | -.056 | 1.000 | .202 | -.053 | -.080 | .060 | .107 | .020 | .118 |  | .091 | -.102 | .088 |  |  |  |  |  |  |  |  |  |  |  |  |  |  |  |  |  |  |  |  |  |  |  |  |
|  |  | ModeTranspoCat(2) | .000 | -.010 | .333 | -.015 |  | .033 | -.106 | -.050 | -.075 | .000 | .000 | -.173 | .050 | -.182 | -.009 | .034 | -.228 | .040 | .202 | 1.000 | -.028 | -.046 | -.032 | .039 | .120 | -.035 |  | .108 | .047 | .031 |  |  |  |  |  |  |  |  |  |  |  |  |  |  |  |  |  |  |  |  |  |  |  |  |
|  |  | EducWomenCat(1) | .000 | .004 | -.015 | -.025 |  | -.114 | .044 | .088 | -.164 | .000 | .000 | .052 | -.109 | -.032 | -.072 | -.108 | .068 | .058 | -.053 | -.028 | 1.000 | .718 | -.134 | -.208 | .038 | .056 |  | -.128 | -.015 | -.108 |  |  |  |  |  |  |  |  |  |  |  |  |  |  |  |  |  |  |  |  |  |  |  |  |
|  |  | EducWomenCat(2) | .000 | .009 | -.088 | .014 |  | -.047 | .056 | .063 | -.004 | .000 | .000 | .131 | -.127 | .038 | .011 | -.108 | .138 | -.007 | -.080 | -.046 | .718 | 1.000 | -.075 | -.249 | .091 | -.148 |  | -.156 | .103 | -.017 |  |  |  |  |  |  |  |  |  |  |  |  |  |  |  |  |  |  |  |  |  |  |  |  |
|  |  | EducHusbCat2(1) | .000 | .111 | .273 | .052 |  | .115 | .170 | -.046 | .125 | .000 | .000 | -.005 | .011 | .049 | .005 | .013 | .215 | .000 | .060 | -.032 | -.134 | -.075 | 1.000 | .783 | -.019 | -.007 |  | -.115 | -.015 | .031 |  |  |  |  |  |  |  |  |  |  |  |  |  |  |  |  |  |  |  |  |  |  |  |  |
|  |  | EducHusbCat2(2) | .000 | .131 | .150 | .105 |  | .146 | .109 | -.038 | .214 | .000 | .000 | .010 | .075 | -.017 | -.041 | .092 | .112 | -.054 | .107 | .039 | -.208 | -.249 | .783 | 1.000 | .082 | -.075 |  | -.149 | -.034 | .073 |  |  |  |  |  |  |  |  |  |  |  |  |  |  |  |  |  |  |  |  |  |  |  |  |
|  |  | AttendPWCLast | .000 | -.074 | .129 | .068 |  | .098 | -.007 | .006 | .086 | .000 | .000 | -.240 | .177 | -.026 | .138 | -.100 | -.168 | -.126 | .020 | .120 | .038 | .091 | -.019 | .082 | 1.000 | -.076 |  | -.059 | -.198 | -.169 |  |  |  |  |  |  |  |  |  |  |  |  |  |  |  |  |  |  |  |  |  |  |  |  |
|  |  | OccupWomLast | .000 | .234 | .017 | -.035 |  | -.025 | -.083 | .029 | -.204 | .000 | .000 | .092 | .016 | .058 | -.055 | -.295 | -.055 | .058 | .118 | -.035 | .056 | -.148 | -.007 | -.075 | -.076 | 1.000 |  | .121 | -.199 | -.037 |  |  |  |  |  |  |  |  |  |  |  |  |  |  |  |  |  |  |  |  |  |  |  |  |
|  |  | ReceivedadequateANCServices | .000 | -.020 | -.004 | -.196 |  | -.002 | -.154 | -.146 | -.143 | .000 | .000 | -.170 | .004 | .109 | .062 | .031 | -.101 | -.178 | .091 | .108 | -.128 | -.156 | -.115 | -.149 | -.059 | .121 |  | 1.000 | .016 | .051 |  |  |  |  |  |  |  |  |  |  |  |  |  |  |  |  |  |  |  |  |  |  |  |  |
|  |  | Number_of_live_children(1) | .000 | -.231 | -.042 | .015 |  | .076 | .003 | .014 | -.051 | .000 | .000 | -.011 | .027 | -.111 | -.140 | -.009 | -.064 | .009 | -.102 | .047 | -.015 | .103 | -.015 | -.034 | -.198 | -.199 |  | .016 | 1.000 | .302 |  |  |  |  |  |  |  |  |  |  |  |  |  |  |  |  |  |  |  |  |  |  |  |  |
|  |  | Number_of_live_children(2) | .000 | -.081 | -.134 | -.067 |  | .066 | -.031 | -.050 | .036 | .000 | .000 | .051 | -.062 | .029 | .113 | -.075 | -.154 | -.056 | .088 | .031 | -.108 | -.017 | .031 | .073 | -.169 | -.037 |  | .051 | .302 | 1.000 |  |  |  |  |  |  |  |  |  |  |  |  |  |  |  |  |  |  |  |  |  |  |  |  |
| Step 4 | | Constant | 1.000 | .000 | .000 | .000 |  | .000 | .000 | .000 | .000 | -1.000 | -1.000 | .000 | .000 | .000 | .000 | .000 | .000 | .000 | .000 | .000 | .000 | .000 | .000 | .000 | .000 |  |  | .000 | .000 | .000 |  |  |  |  |  |  |  |  |  |  |  |  |  |  |  |  |  |  |  |  |  |  |  |  |
|  |  | residence | .000 | 1.000 | .364 | .139 |  | -.019 | .074 | -.096 | .141 | .000 | .000 | .002 | -.129 | .063 | .267 | -.058 | .133 | .134 | .113 | -.001 | -.011 | .046 | .116 | .154 | -.058 |  |  | -.047 | -.196 | -.076 |  |  |  |  |  |  |  |  |  |  |  |  |  |  |  |  |  |  |  |  |  |  |  |  |
|  |  | time2reach | .000 | .364 | 1.000 | .068 |  | .138 | .074 | -.045 | .086 | .000 | .000 | -.193 | .063 | -.019 | .058 | -.033 | .087 | .025 | .159 | .331 | -.013 | -.085 | .269 | .146 | .128 |  |  | -.003 | -.043 | -.136 |  |  |  |  |  |  |  |  |  |  |  |  |  |  |  |  |  |  |  |  |  |  |  |  |
|  |  | when2begin | .000 | .139 | .068 | 1.000 |  | .055 | .115 | .010 | .175 | .000 | .000 | .075 | -.087 | -.163 | -.156 | -.045 | .108 | .124 | -.121 | -.018 | -.027 | .008 | .053 | .105 | .061 |  |  | -.189 | .010 | -.068 |  |  |  |  |  |  |  |  |  |  |  |  |  |  |  |  |  |  |  |  |  |  |  |  |
|  |  | lastpregnw | .000 | -.019 | .138 | .055 |  | 1.000 | .068 | -.062 | .121 | .000 | .000 | -.018 | .084 | .050 | .054 | -.104 | .008 | -.070 | .041 | .034 | -.115 | -.052 | .114 | .145 | .094 |  |  | -.001 | .076 | .067 |  |  |  |  |  |  |  |  |  |  |  |  |  |  |  |  |  |  |  |  |  |  |  |  |
|  |  | toldpregnc | .000 | .074 | .074 | .115 |  | .068 | 1.000 | -.423 | .163 | .000 | .000 | -.006 | -.052 | -.003 | .043 | .000 | .181 | -.110 | .076 | -.110 | .051 | .043 | .167 | .101 | -.009 |  |  | -.150 | -.020 | -.040 |  |  |  |  |  |  |  |  |  |  |  |  |  |  |  |  |  |  |  |  |  |  |  |  |
|  |  | toldbpcrp | .000 | -.096 | -.045 | .010 |  | -.062 | -.423 | 1.000 | -.115 | .000 | .000 | .040 | -.063 | .066 | .014 | .059 | -.067 | -.068 | -.126 | -.053 | .084 | .068 | -.043 | -.033 | .008 |  |  | -.146 | .019 | -.050 |  |  |  |  |  |  |  |  |  |  |  |  |  |  |  |  |  |  |  |  |  |  |  |  |
|  |  | partneracc | .000 | .141 | .086 | .175 |  | .121 | .163 | -.115 | 1.000 | .000 | .000 | .137 | .037 | .125 | .139 | .095 | .023 | -.207 | .065 | -.084 | -.156 | -.036 | .126 | .203 | .072 |  |  | -.123 | -.099 | .027 |  |  |  |  |  |  |  |  |  |  |  |  |  |  |  |  |  |  |  |  |  |  |  |  |
|  |  | placedeliv(1) | -1.000 | .000 | .000 | .000 |  | .000 | .000 | .000 | .000 | 1.000 | 1.000 | .000 | .000 | .000 | .000 | .000 | .000 | .000 | .000 | .000 | .000 | .000 | .000 | .000 | .000 |  |  | .000 | .000 | .000 |  |  |  |  |  |  |  |  |  |  |  |  |  |  |  |  |  |  |  |  |  |  |  |  |
|  |  | placedeliv(2) | -1.000 | .000 | .000 | .000 |  | .000 | .000 | .000 | .000 | 1.000 | 1.000 | .000 | .000 | .000 | .000 | .000 | .000 | .000 | .000 | .000 | .000 | .000 | .000 | .000 | .000 |  |  | .000 | .000 | .000 |  |  |  |  |  |  |  |  |  |  |  |  |  |  |  |  |  |  |  |  |  |  |  |  |
|  |  | be4dischar | .000 | .002 | -.193 | .075 |  | -.018 | -.006 | .040 | .137 | .000 | .000 | 1.000 | -.278 | .069 | .115 | .008 | .175 | -.069 | -.069 | -.166 | .043 | .146 | .002 | .022 | -.233 |  |  | -.185 | .012 | .057 |  |  |  |  |  |  |  |  |  |  |  |  |  |  |  |  |  |  |  |  |  |  |  |  |
|  |  | informed2r | .000 | -.129 | .063 | -.087 |  | .084 | -.052 | -.063 | .037 | .000 | .000 | -.278 | 1.000 | -.054 | -.121 | .010 | -.149 | .008 | .142 | .049 | -.110 | -.127 | .010 | .076 | .176 |  |  | .000 | .026 | -.062 |  |  |  |  |  |  |  |  |  |  |  |  |  |  |  |  |  |  |  |  |  |  |  |  |
|  |  | Monthly_income(1) | .000 | .063 | -.019 | -.163 |  | .050 | -.003 | .066 | .125 | .000 | .000 | .069 | -.054 | 1.000 | .658 | -.099 | -.042 | -.099 | .099 | -.180 | -.037 | .046 | .050 | -.011 | -.016 |  |  | .103 | -.101 | .031 |  |  |  |  |  |  |  |  |  |  |  |  |  |  |  |  |  |  |  |  |  |  |  |  |
|  |  | Monthly_income(2) | .000 | .267 | .058 | -.156 |  | .054 | .043 | .014 | .139 | .000 | .000 | .115 | -.121 | .658 | 1.000 | -.118 | -.142 | -.141 | .036 | -.011 | -.072 | .000 | .007 | -.041 | .139 |  |  | .067 | -.154 | .110 |  |  |  |  |  |  |  |  |  |  |  |  |  |  |  |  |  |  |  |  |  |  |  |  |
|  |  | AgePregCat(1) | .000 | -.058 | -.033 | -.045 |  | -.104 | .000 | .059 | .095 | .000 | .000 | .008 | .010 | -.099 | -.118 | 1.000 | .131 | -.028 | -.088 | .024 | -.095 | -.161 | .013 | .075 | -.133 |  |  | .070 | -.071 | -.091 |  |  |  |  |  |  |  |  |  |  |  |  |  |  |  |  |  |  |  |  |  |  |  |  |
|  |  | FirstANCBooking | .000 | .133 | .087 | .108 |  | .008 | .181 | -.067 | .023 | .000 | .000 | .175 | -.149 | -.042 | -.142 | .131 | 1.000 | -.017 | -.187 | -.233 | .073 | .131 | .213 | .104 | -.176 |  |  | -.094 | -.076 | -.160 |  |  |  |  |  |  |  |  |  |  |  |  |  |  |  |  |  |  |  |  |  |  |  |  |
|  |  | BPCRPKnowledge | .000 | .134 | .025 | .124 |  | -.070 | -.110 | -.068 | -.207 | .000 | .000 | -.069 | .008 | -.099 | -.141 | -.028 | -.017 | 1.000 | -.059 | .045 | .053 | .002 | .002 | -.047 | -.117 |  |  | -.184 | .022 | -.055 |  |  |  |  |  |  |  |  |  |  |  |  |  |  |  |  |  |  |  |  |  |  |  |  |
|  |  | ModeTranspoCat(1) | .000 | .113 | .159 | -.121 |  | .041 | .076 | -.126 | .065 | .000 | .000 | -.069 | .142 | .099 | .036 | -.088 | -.187 | -.059 | 1.000 | .210 | -.058 | -.063 | .057 | .113 | .033 |  |  | .077 | -.086 | .092 |  |  |  |  |  |  |  |  |  |  |  |  |  |  |  |  |  |  |  |  |  |  |  |  |
|  |  | ModeTranspoCat(2) | .000 | -.001 | .331 | -.018 |  | .034 | -.110 | -.053 | -.084 | .000 | .000 | -.166 | .049 | -.180 | -.011 | .024 | -.233 | .045 | .210 | 1.000 | -.027 | -.054 | -.036 | .034 | .114 |  |  | .114 | .040 | .031 |  |  |  |  |  |  |  |  |  |  |  |  |  |  |  |  |  |  |  |  |  |  |  |  |
|  |  | EducWomenCat(1) | .000 | -.011 | -.013 | -.027 |  | -.115 | .051 | .084 | -.156 | .000 | .000 | .043 | -.110 | -.037 | -.072 | -.095 | .073 | .053 | -.058 | -.027 | 1.000 | .735 | -.129 | -.203 | .043 |  |  | -.132 | -.002 | -.107 |  |  |  |  |  |  |  |  |  |  |  |  |  |  |  |  |  |  |  |  |  |  |  |  |
|  |  | EducWomenCat(2) | .000 | .046 | -.085 | .008 |  | -.052 | .043 | .068 | -.036 | .000 | .000 | .146 | -.127 | .046 | .000 | -.161 | .131 | .002 | -.063 | -.054 | .735 | 1.000 | -.074 | -.263 | .080 |  |  | -.138 | .078 | -.024 |  |  |  |  |  |  |  |  |  |  |  |  |  |  |  |  |  |  |  |  |  |  |  |  |
|  |  | EducHusbCat2(1) | .000 | .116 | .269 | .053 |  | .114 | .167 | -.043 | .126 | .000 | .000 | .002 | .010 | .050 | .007 | .013 | .213 | .002 | .057 | -.036 | -.129 | -.074 | 1.000 | .784 | -.020 |  |  | -.119 | -.022 | .026 |  |  |  |  |  |  |  |  |  |  |  |  |  |  |  |  |  |  |  |  |  |  |  |  |
|  |  | EducHusbCat2(2) | .000 | .154 | .146 | .105 |  | .145 | .101 | -.033 | .203 | .000 | .000 | .022 | .076 | -.011 | -.041 | .075 | .104 | -.047 | .113 | .034 | -.203 | -.263 | .784 | 1.000 | .077 |  |  | -.145 | -.058 | .066 |  |  |  |  |  |  |  |  |  |  |  |  |  |  |  |  |  |  |  |  |  |  |  |  |
|  |  | AttendPWCLast | .000 | -.058 | .128 | .061 |  | .094 | -.009 | .008 | .072 | .000 | .000 | -.233 | .176 | -.016 | .139 | -.133 | -.176 | -.117 | .033 | .114 | .043 | .080 | -.020 | .077 | 1.000 |  |  | -.054 | -.223 | -.174 |  |  |  |  |  |  |  |  |  |  |  |  |  |  |  |  |  |  |  |  |  |  |  |  |
|  |  | ReceivedadequateANCServices | .000 | -.047 | -.003 | -.189 |  | -.001 | -.150 | -.146 | -.123 | .000 | .000 | -.185 | .000 | .103 | .067 | .070 | -.094 | -.184 | .077 | .114 | -.132 | -.138 | -.119 | -.145 | -.054 |  |  | 1.000 | .042 | .059 |  |  |  |  |  |  |  |  |  |  |  |  |  |  |  |  |  |  |  |  |  |  |  |  |
|  |  | Number_of_live_children(1) | .000 | -.196 | -.043 | .010 |  | .076 | -.020 | .019 | -.099 | .000 | .000 | .012 | .026 | -.101 | -.154 | -.071 | -.076 | .022 | -.086 | .040 | -.002 | .078 | -.022 | -.058 | -.223 |  |  | .042 | 1.000 | .301 |  |  |  |  |  |  |  |  |  |  |  |  |  |  |  |  |  |  |  |  |  |  |  |  |
|  |  | Number_of_live_children(2) | .000 | -.076 | -.136 | -.068 |  | .067 | -.040 | -.050 | .027 | .000 | .000 | .057 | -.062 | .031 | .110 | -.091 | -.160 | -.055 | .092 | .031 | -.107 | -.024 | .026 | .066 | -.174 |  |  | .059 | .301 | 1.000 |  |  |  |  |  |  |  |  |  |  |  |  |  |  |  |  |  |  |  |  |  |  |  |  |
| Step 5 | | Constant | 1.000 | .000 | .000 | .000 |  | .000 |  | .000 | .000 | -1.000 | -1.000 | .000 | .000 | .000 | .000 | .000 | .000 | .000 | .000 | .000 | .000 | .000 | .000 | .000 | .000 |  |  | .000 | .000 | .000 |  |  |  |  |  |  |  |  |  |  |  |  |  |  |  |  |  |  |  |  |  |  |  |  |
|  |  | residence | .000 | 1.000 | .356 | .132 |  | -.021 |  | -.063 | .126 | .000 | .000 | .006 | -.122 | .065 | .269 | -.065 | .112 | .144 | .113 | .006 | -.014 | .043 | .100 | .141 | -.056 |  |  | -.032 | -.191 | -.069 |  |  |  |  |  |  |  |  |  |  |  |  |  |  |  |  |  |  |  |  |  |  |  |  |
|  |  | time2reach | .000 | .356 | 1.000 | .064 |  | .134 |  | -.006 | .065 | .000 | .000 | -.192 | .064 | -.020 | .059 | -.040 | .061 | .038 | .150 | .349 | -.012 | -.090 | .253 | .130 | .130 |  |  | .008 | -.034 | -.130 |  |  |  |  |  |  |  |  |  |  |  |  |  |  |  |  |  |  |  |  |  |  |  |  |
|  |  | when2begin | .000 | .132 | .064 | 1.000 |  | .055 |  | .066 | .163 | .000 | .000 | .083 | -.080 | -.162 | -.158 | -.044 | .088 | .141 | -.122 | -.010 | -.032 | .004 | .041 | .095 | .060 |  |  | -.177 | .017 | -.063 |  |  |  |  |  |  |  |  |  |  |  |  |  |  |  |  |  |  |  |  |  |  |  |  |
|  |  | lastpregnw | .000 | -.021 | .134 | .055 |  | 1.000 |  | -.036 | .109 | .000 | .000 | -.021 | .078 | .043 | .043 | -.103 | -.008 | -.058 | .027 | .040 | -.117 | -.056 | .101 | .138 | .090 |  |  | .004 | .079 | .069 |  |  |  |  |  |  |  |  |  |  |  |  |  |  |  |  |  |  |  |  |  |  |  |  |
|  |  | toldbpcrp | .000 | -.063 | -.006 | .066 |  | -.036 |  | 1.000 | -.040 | .000 | .000 | .031 | -.081 | .074 | .037 | .065 | .015 | -.130 | -.108 | -.114 | .114 | .091 | .045 | .020 | .003 |  |  | -.253 | .013 | -.072 |  |  |  |  |  |  |  |  |  |  |  |  |  |  |  |  |  |  |  |  |  |  |  |  |
|  |  | partneracc | .000 | .126 | .065 | .163 |  | .109 |  | -.040 | 1.000 | .000 | .000 | .142 | .044 | .125 | .134 | .095 | -.012 | -.187 | .054 | -.072 | -.168 | -.047 | .102 | .192 | .073 |  |  | -.108 | -.092 | .035 |  |  |  |  |  |  |  |  |  |  |  |  |  |  |  |  |  |  |  |  |  |  |  |  |
|  |  | placedeliv(1) | -1.000 | .000 | .000 | .000 |  | .000 |  | .000 | .000 | 1.000 | 1.000 | .000 | .000 | .000 | .000 | .000 | .000 | .000 | .000 | .000 | .000 | .000 | .000 | .000 | .000 |  |  | .000 | .000 | .000 |  |  |  |  |  |  |  |  |  |  |  |  |  |  |  |  |  |  |  |  |  |  |  |  |
|  |  | placedeliv(2) | -1.000 | .000 | .000 | .000 |  | .000 |  | .000 | .000 | 1.000 | 1.000 | .000 | .000 | .000 | .000 | .000 | .000 | .000 | .000 | .000 | .000 | .000 | .000 | .000 | .000 |  |  | .000 | .000 | .000 |  |  |  |  |  |  |  |  |  |  |  |  |  |  |  |  |  |  |  |  |  |  |  |  |
|  |  | be4dischar | .000 | .006 | -.192 | .083 |  | -.021 |  | .031 | .142 | .000 | .000 | 1.000 | -.281 | .060 | .106 | .009 | .184 | -.072 | -.072 | -.167 | .044 | .145 | .004 | .027 | -.233 |  |  | -.190 | .010 | .055 |  |  |  |  |  |  |  |  |  |  |  |  |  |  |  |  |  |  |  |  |  |  |  |  |
|  |  | informed2r | .000 | -.122 | .064 | -.080 |  | .078 |  | -.081 | .044 | .000 | .000 | -.281 | 1.000 | -.058 | -.121 | .017 | -.141 | .005 | .141 | .045 | -.106 | -.123 | .015 | .080 | .175 |  |  | -.009 | .025 | -.065 |  |  |  |  |  |  |  |  |  |  |  |  |  |  |  |  |  |  |  |  |  |  |  |  |
|  |  | Monthly_income(1) | .000 | .065 | -.020 | -.162 |  | .043 |  | .074 | .125 | .000 | .000 | .060 | -.058 | 1.000 | .656 | -.096 | -.043 | -.100 | .092 | -.184 | -.040 | .043 | .051 | -.009 | -.017 |  |  | .105 | -.098 | .031 |  |  |  |  |  |  |  |  |  |  |  |  |  |  |  |  |  |  |  |  |  |  |  |  |
|  |  | Monthly_income(2) | .000 | .269 | .059 | -.158 |  | .043 |  | .037 | .134 | .000 | .000 | .106 | -.121 | .656 | 1.000 | -.119 | -.160 | -.138 | .029 | -.004 | -.076 | -.007 | -.006 | -.050 | .142 |  |  | .078 | -.155 | .112 |  |  |  |  |  |  |  |  |  |  |  |  |  |  |  |  |  |  |  |  |  |  |  |  |
|  |  | AgePregCat(1) | .000 | -.065 | -.040 | -.044 |  | -.103 |  | .065 | .095 | .000 | .000 | .009 | .017 | -.096 | -.119 | 1.000 | .135 | -.022 | -.090 | .021 | -.093 | -.158 | .012 | .076 | -.135 |  |  | .064 | -.063 | -.089 |  |  |  |  |  |  |  |  |  |  |  |  |  |  |  |  |  |  |  |  |  |  |  |  |
|  |  | FirstANCBooking | .000 | .112 | .061 | .088 |  | -.008 |  | .015 | -.012 | .000 | .000 | .184 | -.141 | -.043 | -.160 | .135 | 1.000 | .005 | -.203 | -.222 | .066 | .126 | .186 | .087 | -.186 |  |  | -.065 | -.067 | -.153 |  |  |  |  |  |  |  |  |  |  |  |  |  |  |  |  |  |  |  |  |  |  |  |  |
|  |  | BPCRPKnowledge | .000 | .144 | .038 | .141 |  | -.058 |  | -.130 | -.187 | .000 | .000 | -.072 | .005 | -.100 | -.138 | -.022 | .005 | 1.000 | -.040 | .036 | .060 | .009 | .025 | -.034 | -.115 |  |  | -.196 | .011 | -.061 |  |  |  |  |  |  |  |  |  |  |  |  |  |  |  |  |  |  |  |  |  |  |  |  |
|  |  | ModeTranspoCat(1) | .000 | .113 | .150 | -.122 |  | .027 |  | -.108 | .054 | .000 | .000 | -.072 | .141 | .092 | .029 | -.090 | -.203 | -.040 | 1.000 | .218 | -.062 | -.069 | .041 | .106 | .035 |  |  | .085 | -.078 | .096 |  |  |  |  |  |  |  |  |  |  |  |  |  |  |  |  |  |  |  |  |  |  |  |  |
|  |  | ModeTranspoCat(2) | .000 | .006 | .349 | -.010 |  | .040 |  | -.114 | -.072 | .000 | .000 | -.167 | .045 | -.184 | -.004 | .021 | -.222 | .036 | .218 | 1.000 | -.020 | -.049 | -.023 | .037 | .116 |  |  | .096 | .037 | .025 |  |  |  |  |  |  |  |  |  |  |  |  |  |  |  |  |  |  |  |  |  |  |  |  |
|  |  | EducWomenCat(1) | .000 | -.014 | -.012 | -.032 |  | -.117 |  | .114 | -.168 | .000 | .000 | .044 | -.106 | -.040 | -.076 | -.093 | .066 | .060 | -.062 | -.020 | 1.000 | .735 | -.138 | -.210 | .039 |  |  | -.127 | .002 | -.101 |  |  |  |  |  |  |  |  |  |  |  |  |  |  |  |  |  |  |  |  |  |  |  |  |
|  |  | EducWomenCat(2) | .000 | .043 | -.090 | .004 |  | -.056 |  | .091 | -.047 | .000 | .000 | .145 | -.123 | .043 | -.007 | -.158 | .126 | .009 | -.069 | -.049 | .735 | 1.000 | -.080 | -.267 | .073 |  |  | -.131 | .085 | -.017 |  |  |  |  |  |  |  |  |  |  |  |  |  |  |  |  |  |  |  |  |  |  |  |  |
|  |  | EducHusbCat2(1) | .000 | .100 | .253 | .041 |  | .101 |  | .045 | .102 | .000 | .000 | .004 | .015 | .051 | -.006 | .012 | .186 | .025 | .041 | -.023 | -.138 | -.080 | 1.000 | .782 | -.025 |  |  | -.093 | -.013 | .037 |  |  |  |  |  |  |  |  |  |  |  |  |  |  |  |  |  |  |  |  |  |  |  |  |
|  |  | EducHusbCat2(2) | .000 | .141 | .130 | .095 |  | .138 |  | .020 | .192 | .000 | .000 | .027 | .080 | -.009 | -.050 | .076 | .087 | -.034 | .106 | .037 | -.210 | -.267 | .782 | 1.000 | .075 |  |  | -.132 | -.056 | .072 |  |  |  |  |  |  |  |  |  |  |  |  |  |  |  |  |  |  |  |  |  |  |  |  |
|  |  | AttendPWCLast | .000 | -.056 | .130 | .060 |  | .090 |  | .003 | .073 | .000 | .000 | -.233 | .175 | -.017 | .142 | -.135 | -.186 | -.115 | .035 | .116 | .039 | .073 | -.025 | .075 | 1.000 |  |  | -.056 | -.222 | -.174 |  |  |  |  |  |  |  |  |  |  |  |  |  |  |  |  |  |  |  |  |  |  |  |  |
|  |  | ReceivedadequateANCServices | .000 | -.032 | .008 | -.177 |  | .004 |  | -.253 | -.108 | .000 | .000 | -.190 | -.009 | .105 | .078 | .064 | -.065 | -.196 | .085 | .096 | -.127 | -.131 | -.093 | -.132 | -.056 |  |  | 1.000 | .040 | .051 |  |  |  |  |  |  |  |  |  |  |  |  |  |  |  |  |  |  |  |  |  |  |  |  |
|  |  | Number_of_live_children(1) | .000 | -.191 | -.034 | .017 |  | .079 |  | .013 | -.092 | .000 | .000 | .010 | .025 | -.098 | -.155 | -.063 | -.067 | .011 | -.078 | .037 | .002 | .085 | -.013 | -.056 | -.222 |  |  | .040 | 1.000 | .296 |  |  |  |  |  |  |  |  |  |  |  |  |  |  |  |  |  |  |  |  |  |  |  |  |
|  |  | Number_of_live_children(2) | .000 | -.069 | -.130 | -.063 |  | .069 |  | -.072 | .035 | .000 | .000 | .055 | -.065 | .031 | .112 | -.089 | -.153 | -.061 | .096 | .025 | -.101 | -.017 | .037 | .072 | -.174 |  |  | .051 | .296 | 1.000 |  |  |  |  |  |  |  |  |  |  |  |  |  |  |  |  |  |  |  |  |  |  |  |  |
| Step 6 | | Constant | 1.000 |  |  |  |  |  |  |  |  |  |  |  |  |  |  |  |  |  |  |  |  |  |  |  |  |  |  |  |  |  | .000 | .000 | .000 | .000 | .000 | .000 | -1.000 | -1.000 | .000 | .000 | .000 | .000 | .000 | .000 | .000 | .000 | .000 | .000 | .000 | .000 | .000 | .000 | .000 | .000 |
|  |  | residence | .000 |  |  |  |  |  |  |  |  |  |  |  |  |  |  |  |  |  |  |  |  |  |  |  |  |  |  |  |  |  | 1.000 | .360 | .121 | -.021 | -.088 | .127 | .000 | .000 | -.003 | -.124 | .065 | .275 | -.064 | .108 | .142 | .120 | .014 | -.017 | .040 | .091 | .133 | -.051 | -.194 | -.067 |
|  |  | time2reach | .000 |  |  |  |  |  |  |  |  |  |  |  |  |  |  |  |  |  |  |  |  |  |  |  |  |  |  |  |  |  | .360 | 1.000 | .060 | .134 | -.015 | .072 | .000 | .000 | -.192 | .059 | -.019 | .064 | -.042 | .063 | .041 | .150 | .348 | -.004 | -.084 | .257 | .135 | .131 | -.039 | -.130 |
|  |  | when2begin | .000 |  |  |  |  |  |  |  |  |  |  |  |  |  |  |  |  |  |  |  |  |  |  |  |  |  |  |  |  |  | .121 | .060 | 1.000 | .053 | .031 | .143 | .000 | .000 | .053 | -.086 | -.154 | -.158 | -.030 | .076 | .116 | -.104 | .010 | -.055 | -.019 | .019 | .072 | .045 | .028 | -.062 |
|  |  | lastpregnw | .000 |  |  |  |  |  |  |  |  |  |  |  |  |  |  |  |  |  |  |  |  |  |  |  |  |  |  |  |  |  | -.021 | .134 | .053 | 1.000 | -.032 | .113 | .000 | .000 | -.026 | .077 | .044 | .050 | -.101 | -.014 | -.065 | .028 | .040 | -.125 | -.061 | .102 | .143 | .098 | .076 | .075 |
|  |  | toldbpcrp | .000 |  |  |  |  |  |  |  |  |  |  |  |  |  |  |  |  |  |  |  |  |  |  |  |  |  |  |  |  |  | -.088 | -.015 | .031 | -.032 | 1.000 | -.071 | .000 | .000 | -.007 | -.095 | .103 | .052 | .080 | -.004 | -.185 | -.092 | -.093 | .084 | .059 | .020 | -.011 | -.019 | .028 | -.053 |
|  |  | partneracc | .000 |  |  |  |  |  |  |  |  |  |  |  |  |  |  |  |  |  |  |  |  |  |  |  |  |  |  |  |  |  | .127 | .072 | .143 | .113 | -.071 | 1.000 | .000 | .000 | .120 | .049 | .141 | .144 | .104 | -.021 | -.212 | .071 | -.059 | -.184 | -.063 | .093 | .182 | .071 | -.092 | .035 |
|  |  | placedeliv(1) | -1.000 |  |  |  |  |  |  |  |  |  |  |  |  |  |  |  |  |  |  |  |  |  |  |  |  |  |  |  |  |  | .000 | .000 | .000 | .000 | .000 | .000 | 1.000 | 1.000 | .000 | .000 | .000 | .000 | .000 | .000 | .000 | .000 | .000 | .000 | .000 | .000 | .000 | .000 | .000 | .000 |
|  |  | placedeliv(2) | -1.000 |  |  |  |  |  |  |  |  |  |  |  |  |  |  |  |  |  |  |  |  |  |  |  |  |  |  |  |  |  | .000 | .000 | .000 | .000 | .000 | .000 | 1.000 | 1.000 | .000 | .000 | .000 | .000 | .000 | .000 | .000 | .000 | .000 | .000 | .000 | .000 | .000 | .000 | .000 | .000 |
|  |  | be4dischar | .000 |  |  |  |  |  |  |  |  |  |  |  |  |  |  |  |  |  |  |  |  |  |  |  |  |  |  |  |  |  | -.003 | -.192 | .053 | -.026 | -.007 | .120 | .000 | .000 | 1.000 | -.285 | .079 | .116 | .025 | .176 | -.114 | -.066 | -.152 | .024 | .121 | -.013 | .002 | -.255 | .017 | .057 |
|  |  | informed2r | .000 |  |  |  |  |  |  |  |  |  |  |  |  |  |  |  |  |  |  |  |  |  |  |  |  |  |  |  |  |  | -.124 | .059 | -.086 | .077 | -.095 | .049 | .000 | .000 | -.285 | 1.000 | -.054 | -.119 | .021 | -.141 | -.004 | .135 | .043 | -.104 | -.121 | .018 | .085 | .180 | .023 | -.066 |
|  |  | Monthly_income(1) | .000 |  |  |  |  |  |  |  |  |  |  |  |  |  |  |  |  |  |  |  |  |  |  |  |  |  |  |  |  |  | .065 | -.019 | -.154 | .044 | .103 | .141 | .000 | .000 | .079 | -.054 | 1.000 | .652 | -.105 | -.034 | -.085 | .084 | -.199 | -.028 | .054 | .064 | .007 | -.007 | -.101 | .030 |
|  |  | Monthly_income(2) | .000 |  |  |  |  |  |  |  |  |  |  |  |  |  |  |  |  |  |  |  |  |  |  |  |  |  |  |  |  |  | .275 | .064 | -.158 | .050 | .052 | .144 | .000 | .000 | .116 | -.119 | .652 | 1.000 | -.126 | -.158 | -.124 | .022 | -.008 | -.074 | -.004 | -.003 | -.046 | .152 | -.160 | .116 |
|  |  | AgePregCat(1) | .000 |  |  |  |  |  |  |  |  |  |  |  |  |  |  |  |  |  |  |  |  |  |  |  |  |  |  |  |  |  | -.064 | -.042 | -.030 | -.101 | .080 | .104 | .000 | .000 | .025 | .021 | -.105 | -.126 | 1.000 | .141 | -.010 | -.093 | .021 | -.086 | -.150 | .018 | .085 | -.133 | -.061 | -.088 |
|  |  | FirstANCBooking | .000 |  |  |  |  |  |  |  |  |  |  |  |  |  |  |  |  |  |  |  |  |  |  |  |  |  |  |  |  |  | .108 | .063 | .076 | -.014 | -.004 | -.021 | .000 | .000 | .176 | -.141 | -.034 | -.158 | .141 | 1.000 | -.010 | -.199 | -.218 | .064 | .126 | .187 | .080 | -.189 | -.065 | -.156 |
|  |  | BPCRPKnowledge | .000 |  |  |  |  |  |  |  |  |  |  |  |  |  |  |  |  |  |  |  |  |  |  |  |  |  |  |  |  |  | .142 | .041 | .116 | -.065 | -.185 | -.212 | .000 | .000 | -.114 | -.004 | -.085 | -.124 | -.010 | -.010 | 1.000 | -.016 | .059 | .038 | -.016 | .000 | -.068 | -.125 | .018 | -.048 |
|  |  | ModeTranspoCat(1) | .000 |  |  |  |  |  |  |  |  |  |  |  |  |  |  |  |  |  |  |  |  |  |  |  |  |  |  |  |  |  | .120 | .150 | -.104 | .028 | -.092 | .071 | .000 | .000 | -.066 | .135 | .084 | .022 | -.093 | -.199 | -.016 | 1.000 | .211 | -.053 | -.060 | .050 | .120 | .044 | -.083 | .091 |
|  |  | ModeTranspoCat(2) | .000 |  |  |  |  |  |  |  |  |  |  |  |  |  |  |  |  |  |  |  |  |  |  |  |  |  |  |  |  |  | .014 | .348 | .010 | .040 | -.093 | -.059 | .000 | .000 | -.152 | .043 | -.199 | -.008 | .021 | -.218 | .059 | .211 | 1.000 | -.010 | -.039 | -.007 | .060 | .117 | .038 | .031 |
|  |  | EducWomenCat(1) | .000 |  |  |  |  |  |  |  |  |  |  |  |  |  |  |  |  |  |  |  |  |  |  |  |  |  |  |  |  |  | -.017 | -.004 | -.055 | -.125 | .084 | -.184 | .000 | .000 | .024 | -.104 | -.028 | -.074 | -.086 | .064 | .038 | -.053 | -.010 | 1.000 | .731 | -.143 | -.230 | .027 | .008 | -.100 |
|  |  | EducWomenCat(2) | .000 |  |  |  |  |  |  |  |  |  |  |  |  |  |  |  |  |  |  |  |  |  |  |  |  |  |  |  |  |  | .040 | -.084 | -.019 | -.061 | .059 | -.063 | .000 | .000 | .121 | -.121 | .054 | -.004 | -.150 | .126 | -.016 | -.060 | -.039 | .731 | 1.000 | -.082 | -.285 | .061 | .094 | -.013 |
|  |  | EducHusbCat2(1) | .000 |  |  |  |  |  |  |  |  |  |  |  |  |  |  |  |  |  |  |  |  |  |  |  |  |  |  |  |  |  | .091 | .257 | .019 | .102 | .020 | .093 | .000 | .000 | -.013 | .018 | .064 | -.003 | .018 | .187 | .000 | .050 | -.007 | -.143 | -.082 | 1.000 | .777 | -.032 | -.007 | .039 |
|  |  | EducHusbCat2(2) | .000 |  |  |  |  |  |  |  |  |  |  |  |  |  |  |  |  |  |  |  |  |  |  |  |  |  |  |  |  |  | .133 | .135 | .072 | .143 | -.011 | .182 | .000 | .000 | .002 | .085 | .007 | -.046 | .085 | .080 | -.068 | .120 | .060 | -.230 | -.285 | .777 | 1.000 | .069 | -.050 | .077 |
|  |  | AttendPWCLast | .000 |  |  |  |  |  |  |  |  |  |  |  |  |  |  |  |  |  |  |  |  |  |  |  |  |  |  |  |  |  | -.051 | .131 | .045 | .098 | -.019 | .071 | .000 | .000 | -.255 | .180 | -.007 | .152 | -.133 | -.189 | -.125 | .044 | .117 | .027 | .061 | -.032 | .069 | 1.000 | -.224 | -.170 |
|  |  | Number_of_live_children(1) | .000 |  |  |  |  |  |  |  |  |  |  |  |  |  |  |  |  |  |  |  |  |  |  |  |  |  |  |  |  |  | -.194 | -.039 | .028 | .076 | .028 | -.092 | .000 | .000 | .017 | .023 | -.101 | -.160 | -.061 | -.065 | .018 | -.083 | .038 | .008 | .094 | -.007 | -.050 | -.224 | 1.000 | .293 |
|  |  | Number_of_live_children(2) | .000 |  |  |  |  |  |  |  |  |  |  |  |  |  |  |  |  |  |  |  |  |  |  |  |  |  |  |  |  |  | -.067 | -.130 | -.062 | .075 | -.053 | .035 | .000 | .000 | .057 | -.066 | .030 | .116 | -.088 | -.156 | -.048 | .091 | .031 | -.100 | -.013 | .039 | .077 | -.170 | .293 | 1.000 |
| Step 7 | | Constant | 1.000 |  |  |  |  |  |  |  |  |  |  |  |  |  |  |  |  |  |  |  |  |  |  |  |  |  |  |  |  |  | .000 | .000 | .000 | .000 | .000 | .000 | -1.000 | -1.000 | .000 | .000 | .000 | .000 |  | .000 | .000 | .000 | .000 | .000 | .000 | .000 | .000 | .000 | .000 | .000 |
|  |  | residence | .000 |  |  |  |  |  |  |  |  |  |  |  |  |  |  |  |  |  |  |  |  |  |  |  |  |  |  |  |  |  | 1.000 | .356 | .118 | -.037 | -.084 | .132 | .000 | .000 | .005 | -.126 | .058 | .270 |  | .118 | .135 | .110 | .018 | -.023 | .030 | .089 | .135 | -.061 | -.196 | -.065 |
|  |  | time2reach | .000 |  |  |  |  |  |  |  |  |  |  |  |  |  |  |  |  |  |  |  |  |  |  |  |  |  |  |  |  |  | .356 | 1.000 | .053 | .128 | -.009 | .074 | .000 | .000 | -.196 | .060 | -.021 | .064 |  | .066 | .042 | .149 | .351 | -.013 | -.096 | .254 | .135 | .129 | -.037 | -.129 |
|  |  | when2begin | .000 |  |  |  |  |  |  |  |  |  |  |  |  |  |  |  |  |  |  |  |  |  |  |  |  |  |  |  |  |  | .118 | .053 | 1.000 | .045 | .035 | .143 | .000 | .000 | .053 | -.085 | -.157 | -.165 |  | .084 | .117 | -.107 | .011 | -.057 | -.021 | .018 | .071 | .040 | .030 | -.066 |
|  |  | lastpregnw | .000 |  |  |  |  |  |  |  |  |  |  |  |  |  |  |  |  |  |  |  |  |  |  |  |  |  |  |  |  |  | -.037 | .128 | .045 | 1.000 | -.022 | .127 | .000 | .000 | -.026 | .082 | .044 | .047 |  | .002 | -.066 | .018 | .045 | -.139 | -.082 | .106 | .153 | .089 | .073 | .072 |
|  |  | toldbpcrp | .000 |  |  |  |  |  |  |  |  |  |  |  |  |  |  |  |  |  |  |  |  |  |  |  |  |  |  |  |  |  | -.084 | -.009 | .035 | -.022 | 1.000 | -.075 | .000 | .000 | -.008 | -.098 | .116 | .064 |  | -.021 | -.185 | -.082 | -.095 | .092 | .073 | .020 | -.016 | -.006 | .037 | -.047 |
|  |  | partneracc | .000 |  |  |  |  |  |  |  |  |  |  |  |  |  |  |  |  |  |  |  |  |  |  |  |  |  |  |  |  |  | .132 | .074 | .143 | .127 | -.075 | 1.000 | .000 | .000 | .113 | .049 | .157 | .160 |  | -.039 | -.217 | .079 | -.061 | -.176 | -.046 | .089 | .168 | .086 | -.083 | .049 |
|  |  | placedeliv(1) | -1.000 |  |  |  |  |  |  |  |  |  |  |  |  |  |  |  |  |  |  |  |  |  |  |  |  |  |  |  |  |  | .000 | .000 | .000 | .000 | .000 | .000 | 1.000 | 1.000 | .000 | .000 | .000 | .000 |  | .000 | .000 | .000 | .000 | .000 | .000 | .000 | .000 | .000 | .000 | .000 |
|  |  | placedeliv(2) | -1.000 |  |  |  |  |  |  |  |  |  |  |  |  |  |  |  |  |  |  |  |  |  |  |  |  |  |  |  |  |  | .000 | .000 | .000 | .000 | .000 | .000 | 1.000 | 1.000 | .000 | .000 | .000 | .000 |  | .000 | .000 | .000 | .000 | .000 | .000 | .000 | .000 | .000 | .000 | .000 |
|  |  | be4dischar | .000 |  |  |  |  |  |  |  |  |  |  |  |  |  |  |  |  |  |  |  |  |  |  |  |  |  |  |  |  |  | .005 | -.196 | .053 | -.026 | -.008 | .113 | .000 | .000 | 1.000 | -.273 | .083 | .124 |  | .167 | -.114 | -.065 | -.150 | .024 | .125 | -.015 | -.001 | -.251 | .014 | .057 |
|  |  | informed2r | .000 |  |  |  |  |  |  |  |  |  |  |  |  |  |  |  |  |  |  |  |  |  |  |  |  |  |  |  |  |  | -.126 | .060 | -.085 | .082 | -.098 | .049 | .000 | .000 | -.273 | 1.000 | -.051 | -.116 |  | -.141 | -.005 | .143 | .041 | -.110 | -.127 | .018 | .082 | .185 | .022 | -.067 |
|  |  | Monthly_income(1) | .000 |  |  |  |  |  |  |  |  |  |  |  |  |  |  |  |  |  |  |  |  |  |  |  |  |  |  |  |  |  | .058 | -.021 | -.157 | .044 | .116 | .157 | .000 | .000 | .083 | -.051 | 1.000 | .648 |  | -.018 | -.097 | .073 | -.204 | -.038 | .037 | .066 | .021 | -.022 | -.095 | .025 |
|  |  | Monthly_income(2) | .000 |  |  |  |  |  |  |  |  |  |  |  |  |  |  |  |  |  |  |  |  |  |  |  |  |  |  |  |  |  | .270 | .064 | -.165 | .047 | .064 | .160 | .000 | .000 | .124 | -.116 | .648 | 1.000 |  | -.142 | -.132 | .008 | -.010 | -.085 | -.026 | -.002 | -.032 | .136 | -.159 | .114 |
|  |  | FirstANCBooking | .000 |  |  |  |  |  |  |  |  |  |  |  |  |  |  |  |  |  |  |  |  |  |  |  |  |  |  |  |  |  | .118 | .066 | .084 | .002 | -.021 | -.039 | .000 | .000 | .167 | -.141 | -.018 | -.142 |  | 1.000 | -.012 | -.192 | -.227 | .072 | .145 | .185 | .072 | -.175 | -.055 | -.147 |
|  |  | BPCRPKnowledge | .000 |  |  |  |  |  |  |  |  |  |  |  |  |  |  |  |  |  |  |  |  |  |  |  |  |  |  |  |  |  | .135 | .042 | .117 | -.066 | -.185 | -.217 | .000 | .000 | -.114 | -.005 | -.097 | -.132 |  | -.012 | 1.000 | -.023 | .058 | .037 | -.021 | -.006 | -.075 | -.127 | .020 | -.049 |
|  |  | ModeTranspoCat(1) | .000 |  |  |  |  |  |  |  |  |  |  |  |  |  |  |  |  |  |  |  |  |  |  |  |  |  |  |  |  |  | .110 | .149 | -.107 | .018 | -.082 | .079 | .000 | .000 | -.065 | .143 | .073 | .008 |  | -.192 | -.023 | 1.000 | .217 | -.052 | -.068 | .048 | .127 | .033 | -.088 | .084 |
|  |  | ModeTranspoCat(2) | .000 |  |  |  |  |  |  |  |  |  |  |  |  |  |  |  |  |  |  |  |  |  |  |  |  |  |  |  |  |  | .018 | .351 | .011 | .045 | -.095 | -.061 | .000 | .000 | -.150 | .041 | -.204 | -.010 |  | -.227 | .058 | .217 | 1.000 | -.007 | -.037 | -.010 | .055 | .122 | .037 | .032 |
|  |  | EducWomenCat(1) | .000 |  |  |  |  |  |  |  |  |  |  |  |  |  |  |  |  |  |  |  |  |  |  |  |  |  |  |  |  |  | -.023 | -.013 | -.057 | -.139 | .092 | -.176 | .000 | .000 | .024 | -.110 | -.038 | -.085 |  | .072 | .037 | -.052 | -.007 | 1.000 | .734 | -.138 | -.220 | .019 | .002 | -.114 |
|  |  | EducWomenCat(2) | .000 |  |  |  |  |  |  |  |  |  |  |  |  |  |  |  |  |  |  |  |  |  |  |  |  |  |  |  |  |  | .030 | -.096 | -.021 | -.082 | .073 | -.046 | .000 | .000 | .125 | -.127 | .037 | -.026 |  | .145 | -.021 | -.068 | -.037 | .734 | 1.000 | -.078 | -.273 | .045 | .083 | -.034 |
|  |  | EducHusbCat2(1) | .000 |  |  |  |  |  |  |  |  |  |  |  |  |  |  |  |  |  |  |  |  |  |  |  |  |  |  |  |  |  | .089 | .254 | .018 | .106 | .020 | .089 | .000 | .000 | -.015 | .018 | .066 | -.002 |  | .185 | -.006 | .048 | -.010 | -.138 | -.078 | 1.000 | .782 | -.032 | -.008 | .050 |
|  |  | EducHusbCat2(2) | .000 |  |  |  |  |  |  |  |  |  |  |  |  |  |  |  |  |  |  |  |  |  |  |  |  |  |  |  |  |  | .135 | .135 | .071 | .153 | -.016 | .168 | .000 | .000 | -.001 | .082 | .021 | -.032 |  | .072 | -.075 | .127 | .055 | -.220 | -.273 | .782 | 1.000 | .079 | -.049 | .097 |
|  |  | AttendPWCLast | .000 |  |  |  |  |  |  |  |  |  |  |  |  |  |  |  |  |  |  |  |  |  |  |  |  |  |  |  |  |  | -.061 | .129 | .040 | .089 | -.006 | .086 | .000 | .000 | -.251 | .185 | -.022 | .136 |  | -.175 | -.127 | .033 | .122 | .019 | .045 | -.032 | .079 | 1.000 | -.231 | -.182 |
|  |  | Number_of_live_children(1) | .000 |  |  |  |  |  |  |  |  |  |  |  |  |  |  |  |  |  |  |  |  |  |  |  |  |  |  |  |  |  | -.196 | -.037 | .030 | .073 | .037 | -.083 | .000 | .000 | .014 | .022 | -.095 | -.159 |  | -.055 | .020 | -.088 | .037 | .002 | .083 | -.008 | -.049 | -.231 | 1.000 | .287 |
|  |  | Number_of_live_children(2) | .000 |  |  |  |  |  |  |  |  |  |  |  |  |  |  |  |  |  |  |  |  |  |  |  |  |  |  |  |  |  | -.065 | -.129 | -.066 | .072 | -.047 | .049 | .000 | .000 | .057 | -.067 | .025 | .114 |  | -.147 | -.049 | .084 | .032 | -.114 | -.034 | .050 | .097 | -.182 | .287 | 1.000 |
| Step 8 | | Constant | 1.000 |  |  |  |  |  |  |  |  |  |  |  |  |  |  |  |  |  |  |  |  |  |  |  |  |  |  |  |  |  | .000 | .000 |  | .000 | .000 | .000 | -1.000 | -1.000 | .000 | .000 | .000 | .000 |  | .000 | .000 | .000 | .000 | .000 | .000 | .000 | .000 | .000 | .000 | .000 |
|  |  | residence | .000 |  |  |  |  |  |  |  |  |  |  |  |  |  |  |  |  |  |  |  |  |  |  |  |  |  |  |  |  |  | 1.000 | .349 |  | -.047 | -.085 | .111 | .000 | .000 | -.003 | -.122 | .074 | .295 |  | .110 | .118 | .120 | .014 | -.016 | .032 | .087 | .127 | -.065 | -.202 | -.048 |
|  |  | time2reach | .000 |  |  |  |  |  |  |  |  |  |  |  |  |  |  |  |  |  |  |  |  |  |  |  |  |  |  |  |  |  | .349 | 1.000 |  | .122 | -.007 | .058 | .000 | .000 | -.205 | .065 | -.012 | .075 |  | .060 | .040 | .157 | .347 | -.011 | -.101 | .258 | .135 | .128 | -.034 | -.109 |
|  |  | lastpregnw | .000 |  |  |  |  |  |  |  |  |  |  |  |  |  |  |  |  |  |  |  |  |  |  |  |  |  |  |  |  |  | -.047 | .122 |  | 1.000 | -.024 | .117 | .000 | .000 | -.031 | .089 | .050 | .053 |  | -.005 | -.067 | .026 | .045 | -.137 | -.083 | .104 | .149 | .088 | .071 | .077 |
|  |  | toldbpcrp | .000 |  |  |  |  |  |  |  |  |  |  |  |  |  |  |  |  |  |  |  |  |  |  |  |  |  |  |  |  |  | -.085 | -.007 |  | -.024 | 1.000 | -.078 | .000 | .000 | -.009 | -.099 | .119 | .073 |  | -.024 | -.193 | -.081 | -.096 | .094 | .069 | .017 | -.022 | -.015 | .038 | -.043 |
|  |  | partneracc | .000 |  |  |  |  |  |  |  |  |  |  |  |  |  |  |  |  |  |  |  |  |  |  |  |  |  |  |  |  |  | .111 | .058 |  | .117 | -.078 | 1.000 | .000 | .000 | .106 | .059 | .186 | .187 |  | -.048 | -.235 | .098 | -.067 | -.171 | -.046 | .084 | .163 | .083 | -.095 | .054 |
|  |  | placedeliv(1) | -1.000 |  |  |  |  |  |  |  |  |  |  |  |  |  |  |  |  |  |  |  |  |  |  |  |  |  |  |  |  |  | .000 | .000 |  | .000 | .000 | .000 | 1.000 | 1.000 | .000 | .000 | .000 | .000 |  | .000 | .000 | .000 | .000 | .000 | .000 | .000 | .000 | .000 | .000 | .000 |
|  |  | placedeliv(2) | -1.000 |  |  |  |  |  |  |  |  |  |  |  |  |  |  |  |  |  |  |  |  |  |  |  |  |  |  |  |  |  | .000 | .000 |  | .000 | .000 | .000 | 1.000 | 1.000 | .000 | .000 | .000 | .000 |  | .000 | .000 | .000 | .000 | .000 | .000 | .000 | .000 | .000 | .000 | .000 |
|  |  | be4dischar | .000 |  |  |  |  |  |  |  |  |  |  |  |  |  |  |  |  |  |  |  |  |  |  |  |  |  |  |  |  |  | -.003 | -.205 |  | -.031 | -.009 | .106 | .000 | .000 | 1.000 | -.273 | .091 | .137 |  | .156 | -.127 | -.063 | -.149 | .032 | .129 | -.021 | -.008 | -.251 | .012 | .073 |
|  |  | informed2r | .000 |  |  |  |  |  |  |  |  |  |  |  |  |  |  |  |  |  |  |  |  |  |  |  |  |  |  |  |  |  | -.122 | .065 |  | .089 | -.099 | .059 | .000 | .000 | -.273 | 1.000 | -.062 | -.126 |  | -.125 | .016 | .134 | .042 | -.118 | -.136 | .028 | .096 | .190 | .028 | -.070 |
|  |  | Monthly_income(1) | .000 |  |  |  |  |  |  |  |  |  |  |  |  |  |  |  |  |  |  |  |  |  |  |  |  |  |  |  |  |  | .074 | -.012 |  | .050 | .119 | .186 | .000 | .000 | .091 | -.062 | 1.000 | .640 |  | -.010 | -.085 | .058 | -.206 | -.058 | .031 | .065 | .023 | -.013 | -.081 | .026 |
|  |  | Monthly_income(2) | .000 |  |  |  |  |  |  |  |  |  |  |  |  |  |  |  |  |  |  |  |  |  |  |  |  |  |  |  |  |  | .295 | .075 |  | .053 | .073 | .187 | .000 | .000 | .137 | -.126 | .640 | 1.000 |  | -.136 | -.111 | -.010 | -.012 | -.104 | -.033 | -.004 | -.031 | .145 | -.150 | .112 |
|  |  | FirstANCBooking | .000 |  |  |  |  |  |  |  |  |  |  |  |  |  |  |  |  |  |  |  |  |  |  |  |  |  |  |  |  |  | .110 | .060 |  | -.005 | -.024 | -.048 | .000 | .000 | .156 | -.125 | -.010 | -.136 |  | 1.000 | -.027 | -.191 | -.234 | .081 | .151 | .186 | .067 | -.171 | -.052 | -.142 |
|  |  | BPCRPKnowledge | .000 |  |  |  |  |  |  |  |  |  |  |  |  |  |  |  |  |  |  |  |  |  |  |  |  |  |  |  |  |  | .118 | .040 |  | -.067 | -.193 | -.235 | .000 | .000 | -.127 | .016 | -.085 | -.111 |  | -.027 | 1.000 | -.011 | .056 | .047 | -.017 | -.001 | -.079 | -.123 | .019 | -.037 |
|  |  | ModeTranspoCat(1) | .000 |  |  |  |  |  |  |  |  |  |  |  |  |  |  |  |  |  |  |  |  |  |  |  |  |  |  |  |  |  | .120 | .157 |  | .026 | -.081 | .098 | .000 | .000 | -.063 | .134 | .058 | -.010 |  | -.191 | -.011 | 1.000 | .224 | -.065 | -.075 | .050 | .140 | .039 | -.086 | .085 |
|  |  | ModeTranspoCat(2) | .000 |  |  |  |  |  |  |  |  |  |  |  |  |  |  |  |  |  |  |  |  |  |  |  |  |  |  |  |  |  | .014 | .347 |  | .045 | -.096 | -.067 | .000 | .000 | -.149 | .042 | -.206 | -.012 |  | -.234 | .056 | .224 | 1.000 | -.007 | -.037 | -.012 | .052 | .120 | .033 | .035 |
|  |  | EducWomenCat(1) | .000 |  |  |  |  |  |  |  |  |  |  |  |  |  |  |  |  |  |  |  |  |  |  |  |  |  |  |  |  |  | -.016 | -.011 |  | -.137 | .094 | -.171 | .000 | .000 | .032 | -.118 | -.058 | -.104 |  | .081 | .047 | -.065 | -.007 | 1.000 | .731 | -.137 | -.217 | .014 | .005 | -.115 |
|  |  | EducWomenCat(2) | .000 |  |  |  |  |  |  |  |  |  |  |  |  |  |  |  |  |  |  |  |  |  |  |  |  |  |  |  |  |  | .032 | -.101 |  | -.083 | .069 | -.046 | .000 | .000 | .129 | -.136 | .031 | -.033 |  | .151 | -.017 | -.075 | -.037 | .731 | 1.000 | -.079 | -.275 | .043 | .084 | -.035 |
|  |  | EducHusbCat2(1) | .000 |  |  |  |  |  |  |  |  |  |  |  |  |  |  |  |  |  |  |  |  |  |  |  |  |  |  |  |  |  | .087 | .258 |  | .104 | .017 | .084 | .000 | .000 | -.021 | .028 | .065 | -.004 |  | .186 | -.001 | .050 | -.012 | -.137 | -.079 | 1.000 | .782 | -.032 | -.005 | .055 |
|  |  | EducHusbCat2(2) | .000 |  |  |  |  |  |  |  |  |  |  |  |  |  |  |  |  |  |  |  |  |  |  |  |  |  |  |  |  |  | .127 | .135 |  | .149 | -.022 | .163 | .000 | .000 | -.008 | .096 | .023 | -.031 |  | .067 | -.079 | .140 | .052 | -.217 | -.275 | .782 | 1.000 | .077 | -.052 | .102 |
|  |  | AttendPWCLast | .000 |  |  |  |  |  |  |  |  |  |  |  |  |  |  |  |  |  |  |  |  |  |  |  |  |  |  |  |  |  | -.065 | .128 |  | .088 | -.015 | .083 | .000 | .000 | -.251 | .190 | -.013 | .145 |  | -.171 | -.123 | .039 | .120 | .014 | .043 | -.032 | .077 | 1.000 | -.237 | -.188 |
|  |  | Number_of_live_children(1) | .000 |  |  |  |  |  |  |  |  |  |  |  |  |  |  |  |  |  |  |  |  |  |  |  |  |  |  |  |  |  | -.202 | -.034 |  | .071 | .038 | -.095 | .000 | .000 | .012 | .028 | -.081 | -.150 |  | -.052 | .019 | -.086 | .033 | .005 | .084 | -.005 | -.052 | -.237 | 1.000 | .290 |
|  |  | Number_of_live_children(2) | .000 |  |  |  |  |  |  |  |  |  |  |  |  |  |  |  |  |  |  |  |  |  |  |  |  |  |  |  |  |  | -.048 | -.109 |  | .077 | -.043 | .054 | .000 | .000 | .073 | -.070 | .026 | .112 |  | -.142 | -.037 | .085 | .035 | -.115 | -.035 | .055 | .102 | -.188 | .290 | 1.000 |
| Step 9 | | Constant | 1.000 |  |  |  |  |  |  |  |  |  |  |  |  |  |  |  |  |  |  |  |  |  |  |  |  |  |  |  |  |  | .000 | .000 |  | .000 | .000 | .000 | -1.000 | -1.000 |  | .000 | .000 | .000 |  | .000 | .000 | .000 | .000 | .000 | .000 | .000 | .000 | .000 | .000 | .000 |
|  |  | residence | .000 |  |  |  |  |  |  |  |  |  |  |  |  |  |  |  |  |  |  |  |  |  |  |  |  |  |  |  |  |  | 1.000 | .365 |  | -.052 | -.076 | .107 | .000 | .000 |  | -.132 | .075 | .299 |  | .108 | .127 | .121 | .019 | -.016 | .031 | .094 | .128 | -.067 | -.200 | -.049 |
|  |  | time2reach | .000 |  |  |  |  |  |  |  |  |  |  |  |  |  |  |  |  |  |  |  |  |  |  |  |  |  |  |  |  |  | .365 | 1.000 |  | .121 | -.009 | .085 | .000 | .000 |  | .016 | .007 | .111 |  | .094 | .011 | .151 | .327 | -.006 | -.081 | .259 | .142 | .092 | -.033 | -.097 |
|  |  | lastpregnw | .000 |  |  |  |  |  |  |  |  |  |  |  |  |  |  |  |  |  |  |  |  |  |  |  |  |  |  |  |  |  | -.052 | .121 |  | 1.000 | -.029 | .124 | .000 | .000 |  | .092 | .047 | .047 |  | .000 | -.082 | .025 | .041 | -.138 | -.084 | .101 | .148 | .079 | .073 | .075 |
|  |  | toldbpcrp | .000 |  |  |  |  |  |  |  |  |  |  |  |  |  |  |  |  |  |  |  |  |  |  |  |  |  |  |  |  |  | -.076 | -.009 |  | -.029 | 1.000 | -.077 | .000 | .000 |  | -.105 | .121 | .075 |  | -.009 | -.202 | -.079 | -.104 | .092 | .072 | .017 | -.020 | -.018 | .032 | -.046 |
|  |  | partneracc | .000 |  |  |  |  |  |  |  |  |  |  |  |  |  |  |  |  |  |  |  |  |  |  |  |  |  |  |  |  |  | .107 | .085 |  | .124 | -.077 | 1.000 | .000 | .000 |  | .091 | .175 | .167 |  | -.067 | -.225 | .109 | -.050 | -.171 | -.062 | .089 | .164 | .117 | -.101 | .041 |
|  |  | placedeliv(1) | -1.000 |  |  |  |  |  |  |  |  |  |  |  |  |  |  |  |  |  |  |  |  |  |  |  |  |  |  |  |  |  | .000 | .000 |  | .000 | .000 | .000 | 1.000 | 1.000 |  | .000 | .000 | .000 |  | .000 | .000 | .000 | .000 | .000 | .000 | .000 | .000 | .000 | .000 | .000 |
|  |  | placedeliv(2) | -1.000 |  |  |  |  |  |  |  |  |  |  |  |  |  |  |  |  |  |  |  |  |  |  |  |  |  |  |  |  |  | .000 | .000 |  | .000 | .000 | .000 | 1.000 | 1.000 |  | .000 | .000 | .000 |  | .000 | .000 | .000 | .000 | .000 | .000 | .000 | .000 | .000 | .000 | .000 |
|  |  | informed2r | .000 |  |  |  |  |  |  |  |  |  |  |  |  |  |  |  |  |  |  |  |  |  |  |  |  |  |  |  |  |  | -.132 | .016 |  | .092 | -.105 | .091 | .000 | .000 |  | 1.000 | -.042 | -.093 |  | -.090 | -.019 | .123 | .004 | -.109 | -.106 | .025 | .100 | .137 | .035 | -.050 |
|  |  | Monthly_income(1) | .000 |  |  |  |  |  |  |  |  |  |  |  |  |  |  |  |  |  |  |  |  |  |  |  |  |  |  |  |  |  | .075 | .007 |  | .047 | .121 | .175 | .000 | .000 |  | -.042 | 1.000 | .638 |  | -.028 | -.075 | .065 | -.190 | -.064 | .018 | .062 | .020 | .012 | -.085 | .013 |
|  |  | Monthly_income(2) | .000 |  |  |  |  |  |  |  |  |  |  |  |  |  |  |  |  |  |  |  |  |  |  |  |  |  |  |  |  |  | .299 | .111 |  | .047 | .075 | .167 | .000 | .000 |  | -.093 | .638 | 1.000 |  | -.163 | -.091 | -.001 | .012 | -.111 | -.057 | -.005 | -.032 | .184 | -.153 | .094 |
|  |  | FirstANCBooking | .000 |  |  |  |  |  |  |  |  |  |  |  |  |  |  |  |  |  |  |  |  |  |  |  |  |  |  |  |  |  | .108 | .094 |  | .000 | -.009 | -.067 | .000 | .000 |  | -.090 | -.028 | -.163 |  | 1.000 | -.006 | -.186 | -.214 | .074 | .131 | .193 | .068 | -.138 | -.050 | -.152 |
|  |  | BPCRPKnowledge | .000 |  |  |  |  |  |  |  |  |  |  |  |  |  |  |  |  |  |  |  |  |  |  |  |  |  |  |  |  |  | .127 | .011 |  | -.082 | -.202 | -.225 | .000 | .000 |  | -.019 | -.075 | -.091 |  | -.006 | 1.000 | -.024 | .038 | .054 | .002 | .000 | -.072 | -.162 | .021 | -.021 |
|  |  | ModeTranspoCat(1) | .000 |  |  |  |  |  |  |  |  |  |  |  |  |  |  |  |  |  |  |  |  |  |  |  |  |  |  |  |  |  | .121 | .151 |  | .025 | -.079 | .109 | .000 | .000 |  | .123 | .065 | -.001 |  | -.186 | -.024 | 1.000 | .217 | -.064 | -.066 | .062 | .150 | .031 | -.092 | .090 |
|  |  | ModeTranspoCat(2) | .000 |  |  |  |  |  |  |  |  |  |  |  |  |  |  |  |  |  |  |  |  |  |  |  |  |  |  |  |  |  | .019 | .327 |  | .041 | -.104 | -.050 | .000 | .000 |  | .004 | -.190 | .012 |  | -.214 | .038 | .217 | 1.000 | -.002 | -.018 | -.017 | .048 | .088 | .037 | .048 |
|  |  | EducWomenCat(1) | .000 |  |  |  |  |  |  |  |  |  |  |  |  |  |  |  |  |  |  |  |  |  |  |  |  |  |  |  |  |  | -.016 | -.006 |  | -.138 | .092 | -.171 | .000 | .000 |  | -.109 | -.064 | -.111 |  | .074 | .054 | -.064 | -.002 | 1.000 | .738 | -.141 | -.220 | .022 | .008 | -.118 |
|  |  | EducWomenCat(2) | .000 |  |  |  |  |  |  |  |  |  |  |  |  |  |  |  |  |  |  |  |  |  |  |  |  |  |  |  |  |  | .031 | -.081 |  | -.084 | .072 | -.062 | .000 | .000 |  | -.106 | .018 | -.057 |  | .131 | .002 | -.066 | -.018 | .738 | 1.000 | -.089 | -.285 | .073 | .087 | -.044 |
|  |  | EducHusbCat2(1) | .000 |  |  |  |  |  |  |  |  |  |  |  |  |  |  |  |  |  |  |  |  |  |  |  |  |  |  |  |  |  | .094 | .259 |  | .101 | .017 | .089 | .000 | .000 |  | .025 | .062 | -.005 |  | .193 | .000 | .062 | -.017 | -.141 | -.089 | 1.000 | .788 | -.032 | -.006 | .058 |
|  |  | EducHusbCat2(2) | .000 |  |  |  |  |  |  |  |  |  |  |  |  |  |  |  |  |  |  |  |  |  |  |  |  |  |  |  |  |  | .128 | .142 |  | .148 | -.020 | .164 | .000 | .000 |  | .100 | .020 | -.032 |  | .068 | -.072 | .150 | .048 | -.220 | -.285 | .788 | 1.000 | .082 | -.050 | .103 |
|  |  | AttendPWCLast | .000 |  |  |  |  |  |  |  |  |  |  |  |  |  |  |  |  |  |  |  |  |  |  |  |  |  |  |  |  |  | -.067 | .092 |  | .079 | -.018 | .117 | .000 | .000 |  | .137 | .012 | .184 |  | -.138 | -.162 | .031 | .088 | .022 | .073 | -.032 | .082 | 1.000 | -.242 | -.183 |
|  |  | Number_of_live_children(1) | .000 |  |  |  |  |  |  |  |  |  |  |  |  |  |  |  |  |  |  |  |  |  |  |  |  |  |  |  |  |  | -.200 | -.033 |  | .073 | .032 | -.101 | .000 | .000 |  | .035 | -.085 | -.153 |  | -.050 | .021 | -.092 | .037 | .008 | .087 | -.006 | -.050 | -.242 | 1.000 | .293 |
|  |  | Number_of_live_children(2) | .000 |  |  |  |  |  |  |  |  |  |  |  |  |  |  |  |  |  |  |  |  |  |  |  |  |  |  |  |  |  | -.049 | -.097 |  | .075 | -.046 | .041 | .000 | .000 |  | -.050 | .013 | .094 |  | -.152 | -.021 | .090 | .048 | -.118 | -.044 | .058 | .103 | -.183 | .293 | 1.000 |
| Step 10 | | Constant | 1.000 |  |  |  |  |  |  |  |  |  |  |  |  |  |  |  |  |  |  |  |  |  |  |  |  |  |  |  |  |  | .000 | .000 |  | .000 | .000 | .000 | -1.000 | -1.000 |  | .000 | .000 | .000 |  | .000 |  | .000 | .000 | .000 | .000 | .000 | .000 | .000 | .000 | .000 |
|  |  | residence | .000 |  |  |  |  |  |  |  |  |  |  |  |  |  |  |  |  |  |  |  |  |  |  |  |  |  |  |  |  |  | 1.000 | .375 |  | -.041 | -.056 | .145 | .000 | .000 |  | -.128 | .088 | .315 |  | .105 |  | .128 | .016 | -.019 | .031 | .095 | .139 | -.051 | -.202 | -.051 |
|  |  | time2reach | .000 |  |  |  |  |  |  |  |  |  |  |  |  |  |  |  |  |  |  |  |  |  |  |  |  |  |  |  |  |  | .375 | 1.000 |  | .118 | -.009 | .100 | .000 | .000 |  | .013 | .012 | .123 |  | .095 |  | .157 | .327 | -.001 | -.075 | .263 | .145 | .084 | -.033 | -.090 |
|  |  | lastpregnw | .000 |  |  |  |  |  |  |  |  |  |  |  |  |  |  |  |  |  |  |  |  |  |  |  |  |  |  |  |  |  | -.041 | .118 |  | 1.000 | -.049 | .106 | .000 | .000 |  | .087 | .042 | .045 |  | -.004 |  | .021 | .044 | -.132 | -.081 | .098 | .138 | .069 | .074 | .074 |
|  |  | toldbpcrp | .000 |  |  |  |  |  |  |  |  |  |  |  |  |  |  |  |  |  |  |  |  |  |  |  |  |  |  |  |  |  | -.056 | -.009 |  | -.049 | 1.000 | -.132 | .000 | .000 |  | -.113 | .107 | .059 |  | -.012 |  | -.078 | -.097 | .096 | .073 | .020 | -.036 | -.049 | .040 | -.051 |
|  |  | partneracc | .000 |  |  |  |  |  |  |  |  |  |  |  |  |  |  |  |  |  |  |  |  |  |  |  |  |  |  |  |  |  | .145 | .100 |  | .106 | -.132 | 1.000 | .000 | .000 |  | .080 | .160 | .150 |  | -.066 |  | .113 | -.035 | -.155 | -.062 | .085 | .144 | .071 | -.100 | .033 |
|  |  | placedeliv(1) | -1.000 |  |  |  |  |  |  |  |  |  |  |  |  |  |  |  |  |  |  |  |  |  |  |  |  |  |  |  |  |  | .000 | .000 |  | .000 | .000 | .000 | 1.000 | 1.000 |  | .000 | .000 | .000 |  | .000 |  | .000 | .000 | .000 | .000 | .000 | .000 | .000 | .000 | .000 |
|  |  | placedeliv(2) | -1.000 |  |  |  |  |  |  |  |  |  |  |  |  |  |  |  |  |  |  |  |  |  |  |  |  |  |  |  |  |  | .000 | .000 |  | .000 | .000 | .000 | 1.000 | 1.000 |  | .000 | .000 | .000 |  | .000 |  | .000 | .000 | .000 | .000 | .000 | .000 | .000 | .000 | .000 |
|  |  | informed2r | .000 |  |  |  |  |  |  |  |  |  |  |  |  |  |  |  |  |  |  |  |  |  |  |  |  |  |  |  |  |  | -.128 | .013 |  | .087 | -.113 | .080 | .000 | .000 |  | 1.000 | -.046 | -.096 |  | -.080 |  | .128 | .000 | -.110 | -.104 | .027 | .099 | .141 | .039 | -.054 |
|  |  | Monthly_income(1) | .000 |  |  |  |  |  |  |  |  |  |  |  |  |  |  |  |  |  |  |  |  |  |  |  |  |  |  |  |  |  | .088 | .012 |  | .042 | .107 | .160 | .000 | .000 |  | -.046 | 1.000 | .638 |  | -.035 |  | .064 | -.188 | -.057 | .018 | .063 | .014 | .004 | -.082 | .011 |
|  |  | Monthly_income(2) | .000 |  |  |  |  |  |  |  |  |  |  |  |  |  |  |  |  |  |  |  |  |  |  |  |  |  |  |  |  |  | .315 | .123 |  | .045 | .059 | .150 | .000 | .000 |  | -.096 | .638 | 1.000 |  | -.171 |  | -.001 | .015 | -.106 | -.060 | -.001 | -.040 | .173 | -.152 | .089 |
|  |  | FirstANCBooking | .000 |  |  |  |  |  |  |  |  |  |  |  |  |  |  |  |  |  |  |  |  |  |  |  |  |  |  |  |  |  | .105 | .095 |  | -.004 | -.012 | -.066 | .000 | .000 |  | -.080 | -.035 | -.171 |  | 1.000 |  | -.190 | -.219 | .071 | .129 | .190 | .069 | -.145 | -.048 | -.148 |
|  |  | ModeTranspoCat(1) | .000 |  |  |  |  |  |  |  |  |  |  |  |  |  |  |  |  |  |  |  |  |  |  |  |  |  |  |  |  |  | .128 | .157 |  | .021 | -.078 | .113 | .000 | .000 |  | .128 | .064 | -.001 |  | -.190 |  | 1.000 | .221 | -.057 | -.059 | .062 | .145 | .025 | -.089 | .087 |
|  |  | ModeTranspoCat(2) | .000 |  |  |  |  |  |  |  |  |  |  |  |  |  |  |  |  |  |  |  |  |  |  |  |  |  |  |  |  |  | .016 | .327 |  | .044 | -.097 | -.035 | .000 | .000 |  | .000 | -.188 | .015 |  | -.219 |  | .221 | 1.000 | -.005 | -.015 | -.013 | .051 | .085 | .035 | .050 |
|  |  | EducWomenCat(1) | .000 |  |  |  |  |  |  |  |  |  |  |  |  |  |  |  |  |  |  |  |  |  |  |  |  |  |  |  |  |  | -.019 | -.001 |  | -.132 | .096 | -.155 | .000 | .000 |  | -.110 | -.057 | -.106 |  | .071 |  | -.057 | -.005 | 1.000 | .739 | -.141 | -.217 | .030 | .005 | -.111 |
|  |  | EducWomenCat(2) | .000 |  |  |  |  |  |  |  |  |  |  |  |  |  |  |  |  |  |  |  |  |  |  |  |  |  |  |  |  |  | .031 | -.075 |  | -.081 | .073 | -.062 | .000 | .000 |  | -.104 | .018 | -.060 |  | .129 |  | -.059 | -.015 | .739 | 1.000 | -.087 | -.287 | .064 | .087 | -.038 |
|  |  | EducHusbCat2(1) | .000 |  |  |  |  |  |  |  |  |  |  |  |  |  |  |  |  |  |  |  |  |  |  |  |  |  |  |  |  |  | .095 | .263 |  | .098 | .020 | .085 | .000 | .000 |  | .027 | .063 | -.001 |  | .190 |  | .062 | -.013 | -.141 | -.087 | 1.000 | .788 | -.033 | -.010 | .053 |
|  |  | EducHusbCat2(2) | .000 |  |  |  |  |  |  |  |  |  |  |  |  |  |  |  |  |  |  |  |  |  |  |  |  |  |  |  |  |  | .139 | .145 |  | .138 | -.036 | .144 | .000 | .000 |  | .099 | .014 | -.040 |  | .069 |  | .145 | .051 | -.217 | -.287 | .788 | 1.000 | .073 | -.049 | .093 |
|  |  | AttendPWCLast | .000 |  |  |  |  |  |  |  |  |  |  |  |  |  |  |  |  |  |  |  |  |  |  |  |  |  |  |  |  |  | -.051 | .084 |  | .069 | -.049 | .071 | .000 | .000 |  | .141 | .004 | .173 |  | -.145 |  | .025 | .085 | .030 | .064 | -.033 | .073 | 1.000 | -.241 | -.188 |
|  |  | Number_of_live_children(1) | .000 |  |  |  |  |  |  |  |  |  |  |  |  |  |  |  |  |  |  |  |  |  |  |  |  |  |  |  |  |  | -.202 | -.033 |  | .074 | .040 | -.100 | .000 | .000 |  | .039 | -.082 | -.152 |  | -.048 |  | -.089 | .035 | .005 | .087 | -.010 | -.049 | -.241 | 1.000 | .290 |
|  |  | Number_of_live_children(2) | .000 |  |  |  |  |  |  |  |  |  |  |  |  |  |  |  |  |  |  |  |  |  |  |  |  |  |  |  |  |  | -.051 | -.090 |  | .074 | -.051 | .033 | .000 | .000 |  | -.054 | .011 | .089 |  | -.148 |  | .087 | .050 | -.111 | -.038 | .053 | .093 | -.188 | .290 | 1.000 |
| Step 11 | | Constant | 1.000 |  |  |  |  |  |  |  |  |  |  |  |  |  |  |  |  |  |  |  |  |  |  |  |  |  |  |  |  |  | -.606 | -.500 |  | -.332 | -.212 | -.279 |  |  |  | -.141 | -.283 | -.407 |  | -.148 |  | -.168 | -.099 | -.111 | -.202 | -.460 | -.448 | -.164 | .029 | -.043 |
|  |  | residence | -.606 |  |  |  |  |  |  |  |  |  |  |  |  |  |  |  |  |  |  |  |  |  |  |  |  |  |  |  |  |  | 1.000 | .360 |  | -.079 | -.109 | .166 |  |  |  | -.112 | .062 | .309 |  | .114 |  | .129 | .032 | -.022 | .056 | .100 | .150 | -.062 | -.182 | -.014 |
|  |  | time2reach | -.500 |  |  |  |  |  |  |  |  |  |  |  |  |  |  |  |  |  |  |  |  |  |  |  |  |  |  |  |  |  | .360 | 1.000 |  | .098 | -.035 | .093 |  |  |  | .021 | -.004 | .112 |  | .106 |  | .154 | .340 | -.004 | -.071 | .261 | .137 | .078 | -.021 | -.083 |
|  |  | lastpregnw | -.332 |  |  |  |  |  |  |  |  |  |  |  |  |  |  |  |  |  |  |  |  |  |  |  |  |  |  |  |  |  | -.079 | .098 |  | 1.000 | -.066 | .101 |  |  |  | .087 | .027 | .029 |  | -.008 |  | .022 | .042 | -.138 | -.083 | .093 | .138 | .057 | .083 | .090 |
|  |  | toldbpcrp | -.212 |  |  |  |  |  |  |  |  |  |  |  |  |  |  |  |  |  |  |  |  |  |  |  |  |  |  |  |  |  | -.109 | -.035 |  | -.066 | 1.000 | -.133 |  |  |  | -.121 | .097 | .054 |  | -.019 |  | -.090 | -.098 | .096 | .074 | .006 | -.042 | -.050 | .041 | -.047 |
|  |  | partneracc | -.279 |  |  |  |  |  |  |  |  |  |  |  |  |  |  |  |  |  |  |  |  |  |  |  |  |  |  |  |  |  | .166 | .093 |  | .101 | -.133 | 1.000 |  |  |  | .088 | .160 | .146 |  | -.065 |  | .108 | -.042 | -.173 | -.075 | .099 | .158 | .082 | -.093 | .024 |
|  |  | informed2r | -.141 |  |  |  |  |  |  |  |  |  |  |  |  |  |  |  |  |  |  |  |  |  |  |  |  |  |  |  |  |  | -.112 | .021 |  | .087 | -.121 | .088 |  |  |  | 1.000 | -.036 | -.097 |  | -.076 |  | .123 | .000 | -.102 | -.098 | .018 | .097 | .138 | .022 | -.054 |
|  |  | Monthly_income(1) | -.283 |  |  |  |  |  |  |  |  |  |  |  |  |  |  |  |  |  |  |  |  |  |  |  |  |  |  |  |  |  | .062 | -.004 |  | .027 | .097 | .160 |  |  |  | -.036 | 1.000 | .634 |  | -.038 |  | .057 | -.181 | -.052 | .026 | .054 | .005 | .003 | -.076 | -.002 |
|  |  | Monthly_income(2) | -.407 |  |  |  |  |  |  |  |  |  |  |  |  |  |  |  |  |  |  |  |  |  |  |  |  |  |  |  |  |  | .309 | .112 |  | .029 | .054 | .146 |  |  |  | -.097 | .634 | 1.000 |  | -.186 |  | .001 | .030 | -.085 | -.042 | -.020 | -.060 | .161 | -.156 | .095 |
|  |  | FirstANCBooking | -.148 |  |  |  |  |  |  |  |  |  |  |  |  |  |  |  |  |  |  |  |  |  |  |  |  |  |  |  |  |  | .114 | .106 |  | -.008 | -.019 | -.065 |  |  |  | -.076 | -.038 | -.186 |  | 1.000 |  | -.176 | -.211 | .098 | .149 | .184 | .061 | -.143 | -.057 | -.166 |
|  |  | ModeTranspoCat(1) | -.168 |  |  |  |  |  |  |  |  |  |  |  |  |  |  |  |  |  |  |  |  |  |  |  |  |  |  |  |  |  | .129 | .154 |  | .022 | -.090 | .108 |  |  |  | .123 | .057 | .001 |  | -.176 |  | 1.000 | .221 | -.081 | -.067 | .070 | .146 | .039 | -.086 | .092 |
|  |  | ModeTranspoCat(2) | -.099 |  |  |  |  |  |  |  |  |  |  |  |  |  |  |  |  |  |  |  |  |  |  |  |  |  |  |  |  |  | .032 | .340 |  | .042 | -.098 | -.042 |  |  |  | .000 | -.181 | .030 |  | -.211 |  | .221 | 1.000 | -.022 | -.032 | -.020 | .046 | .097 | .034 | .059 |
|  |  | EducWomenCat(1) | -.111 |  |  |  |  |  |  |  |  |  |  |  |  |  |  |  |  |  |  |  |  |  |  |  |  |  |  |  |  |  | -.022 | -.004 |  | -.138 | .096 | -.173 |  |  |  | -.102 | -.052 | -.085 |  | .098 |  | -.081 | -.022 | 1.000 | .742 | -.140 | -.224 | .045 | .016 | -.127 |
|  |  | EducWomenCat(2) | -.202 |  |  |  |  |  |  |  |  |  |  |  |  |  |  |  |  |  |  |  |  |  |  |  |  |  |  |  |  |  | .056 | -.071 |  | -.083 | .074 | -.075 |  |  |  | -.098 | .026 | -.042 |  | .149 |  | -.067 | -.032 | .742 | 1.000 | -.083 | -.292 | .083 | .086 | -.061 |
|  |  | EducHusbCat2(1) | -.460 |  |  |  |  |  |  |  |  |  |  |  |  |  |  |  |  |  |  |  |  |  |  |  |  |  |  |  |  |  | .100 | .261 |  | .093 | .006 | .099 |  |  |  | .018 | .054 | -.020 |  | .184 |  | .070 | -.020 | -.140 | -.083 | 1.000 | .782 | -.038 | -.024 | .069 |
|  |  | EducHusbCat2(2) | -.448 |  |  |  |  |  |  |  |  |  |  |  |  |  |  |  |  |  |  |  |  |  |  |  |  |  |  |  |  |  | .150 | .137 |  | .138 | -.042 | .158 |  |  |  | .097 | .005 | -.060 |  | .061 |  | .146 | .046 | -.224 | -.292 | .782 | 1.000 | .072 | -.059 | .113 |
|  |  | AttendPWCLast | -.164 |  |  |  |  |  |  |  |  |  |  |  |  |  |  |  |  |  |  |  |  |  |  |  |  |  |  |  |  |  | -.062 | .078 |  | .057 | -.050 | .082 |  |  |  | .138 | .003 | .161 |  | -.143 |  | .039 | .097 | .045 | .083 | -.038 | .072 | 1.000 | -.242 | -.180 |
|  |  | Number_of_live_children(1) | .029 |  |  |  |  |  |  |  |  |  |  |  |  |  |  |  |  |  |  |  |  |  |  |  |  |  |  |  |  |  | -.182 | -.021 |  | .083 | .041 | -.093 |  |  |  | .022 | -.076 | -.156 |  | -.057 |  | -.086 | .034 | .016 | .086 | -.024 | -.059 | -.242 | 1.000 | .278 |
|  |  | Number_of_live_children(2) | -.043 |  |  |  |  |  |  |  |  |  |  |  |  |  |  |  |  |  |  |  |  |  |  |  |  |  |  |  |  |  | -.014 | -.083 |  | .090 | -.047 | .024 |  |  |  | -.054 | -.002 | .095 |  | -.166 |  | .092 | .059 | -.127 | -.061 | .069 | .113 | -.180 | .278 | 1.000 |
| Step 12 | | Constant | 1.000 |  |  |  |  |  |  |  |  |  |  |  |  |  |  |  |  |  |  |  |  |  |  |  |  |  |  |  |  |  | -.626 | -.464 |  | -.308 | -.260 | -.249 |  |  |  | -.152 | -.299 | -.481 |  | -.099 |  | -.133 | -.105 | -.231 | -.358 |  |  | -.180 | .011 | .002 |
|  |  | residence | -.626 |  |  |  |  |  |  |  |  |  |  |  |  |  |  |  |  |  |  |  |  |  |  |  |  |  |  |  |  |  | 1.000 | .360 |  | -.109 | -.090 | .146 |  |  |  | -.096 | .058 | .310 |  | .128 |  | .103 | .006 | .005 | .113 |  |  | -.087 | -.170 | -.029 |
|  |  | time2reach | -.464 |  |  |  |  |  |  |  |  |  |  |  |  |  |  |  |  |  |  |  |  |  |  |  |  |  |  |  |  |  | .360 | 1.000 |  | .092 | -.039 | .095 |  |  |  | .042 | -.016 | .116 |  | .059 |  | .163 | .377 | -.029 | -.122 |  |  | .116 | -.043 | -.096 |
|  |  | lastpregnw | -.308 |  |  |  |  |  |  |  |  |  |  |  |  |  |  |  |  |  |  |  |  |  |  |  |  |  |  |  |  |  | -.109 | .092 |  | 1.000 | -.053 | .076 |  |  |  | .076 | .026 | .033 |  | -.016 |  | -.004 | .042 | -.118 | -.052 |  |  | .047 | .087 | .065 |
|  |  | toldbpcrp | -.260 |  |  |  |  |  |  |  |  |  |  |  |  |  |  |  |  |  |  |  |  |  |  |  |  |  |  |  |  |  | -.090 | -.039 |  | -.053 | 1.000 | -.138 |  |  |  | -.125 | .083 | .052 |  | -.035 |  | -.073 | -.076 | .080 | .044 |  |  | -.026 | .038 | -.040 |
|  |  | partneracc | -.249 |  |  |  |  |  |  |  |  |  |  |  |  |  |  |  |  |  |  |  |  |  |  |  |  |  |  |  |  |  | .146 | .095 |  | .076 | -.138 | 1.000 |  |  |  | .074 | .147 | .159 |  | -.059 |  | .074 | -.053 | -.127 | -.021 |  |  | .046 | -.044 | .035 |
|  |  | informed2r | -.152 |  |  |  |  |  |  |  |  |  |  |  |  |  |  |  |  |  |  |  |  |  |  |  |  |  |  |  |  |  | -.096 | .042 |  | .076 | -.125 | .074 |  |  |  | 1.000 | .002 | -.056 |  | -.077 |  | .107 | .001 | -.099 | -.062 |  |  | .130 | .014 | -.075 |
|  |  | Monthly_income(1) | -.299 |  |  |  |  |  |  |  |  |  |  |  |  |  |  |  |  |  |  |  |  |  |  |  |  |  |  |  |  |  | .058 | -.016 |  | .026 | .083 | .147 |  |  |  | .002 | 1.000 | .624 |  | -.026 |  | .066 | -.186 | -.052 | .005 |  |  | -.004 | -.080 | .026 |
|  |  | Monthly_income(2) | -.481 |  |  |  |  |  |  |  |  |  |  |  |  |  |  |  |  |  |  |  |  |  |  |  |  |  |  |  |  |  | .310 | .116 |  | .033 | .052 | .159 |  |  |  | -.056 | .624 | 1.000 |  | -.181 |  | .012 | .024 | -.107 | -.074 |  |  | .164 | -.161 | .128 |
|  |  | FirstANCBooking | -.099 |  |  |  |  |  |  |  |  |  |  |  |  |  |  |  |  |  |  |  |  |  |  |  |  |  |  |  |  |  | .128 | .059 |  | -.016 | -.035 | -.059 |  |  |  | -.077 | -.026 | -.181 |  | 1.000 |  | -.186 | -.199 | .091 | .102 |  |  | -.116 | -.085 | -.197 |
|  |  | ModeTranspoCat(1) | -.133 |  |  |  |  |  |  |  |  |  |  |  |  |  |  |  |  |  |  |  |  |  |  |  |  |  |  |  |  |  | .103 | .163 |  | -.004 | -.073 | .074 |  |  |  | .107 | .066 | .012 |  | -.186 |  | 1.000 | .219 | -.041 | .004 |  |  | .008 | -.073 | .082 |
|  |  | ModeTranspoCat(2) | -.105 |  |  |  |  |  |  |  |  |  |  |  |  |  |  |  |  |  |  |  |  |  |  |  |  |  |  |  |  |  | .006 | .377 |  | .042 | -.076 | -.053 |  |  |  | .001 | -.186 | .024 |  | -.199 |  | .219 | 1.000 | -.035 | -.004 |  |  | .104 | .032 | .035 |
|  |  | EducWomenCat(1) | -.231 |  |  |  |  |  |  |  |  |  |  |  |  |  |  |  |  |  |  |  |  |  |  |  |  |  |  |  |  |  | .005 | -.029 |  | -.118 | .080 | -.127 |  |  |  | -.099 | -.052 | -.107 |  | .091 |  | -.041 | -.035 | 1.000 | .756 |  |  | .068 | -.011 | -.105 |
|  |  | EducWomenCat(2) | -.358 |  |  |  |  |  |  |  |  |  |  |  |  |  |  |  |  |  |  |  |  |  |  |  |  |  |  |  |  |  | .113 | -.122 |  | -.052 | .044 | -.021 |  |  |  | -.062 | .005 | -.074 |  | .102 |  | .004 | -.004 | .756 | 1.000 |  |  | .146 | .063 | -.038 |
|  |  | AttendPWCLast | -.180 |  |  |  |  |  |  |  |  |  |  |  |  |  |  |  |  |  |  |  |  |  |  |  |  |  |  |  |  |  | -.087 | .116 |  | .047 | -.026 | .046 |  |  |  | .130 | -.004 | .164 |  | -.116 |  | .008 | .104 | .068 | .146 |  |  | 1.000 | -.229 | -.201 |
|  |  | Number_of_live_children(1) | .011 |  |  |  |  |  |  |  |  |  |  |  |  |  |  |  |  |  |  |  |  |  |  |  |  |  |  |  |  |  | -.170 | -.043 |  | .087 | .038 | -.044 |  |  |  | .014 | -.080 | -.161 |  | -.085 |  | -.073 | .032 | -.011 | .063 |  |  | -.229 | 1.000 | .285 |
|  |  | Number_of_live_children(2) | .002 |  |  |  |  |  |  |  |  |  |  |  |  |  |  |  |  |  |  |  |  |  |  |  |  |  |  |  |  |  | -.029 | -.096 |  | .065 | -.040 | .035 |  |  |  | -.075 | .026 | .128 |  | -.197 |  | .082 | .035 | -.105 | -.038 |  |  | -.201 | .285 | 1.000 |
| Step 13 | | Constant | 1.000 |  |  |  |  |  |  |  |  |  |  |  |  |  |  |  |  |  |  |  |  |  |  |  |  |  |  |  |  |  |  | -.319 |  | -.496 | -.409 | -.202 |  |  |  | -.291 | -.324 | -.376 |  | -.012 |  | -.075 | -.122 | -.291 | -.371 |  |  | -.295 | -.131 | -.019 |
|  |  | time2reach | -.319 |  |  |  |  |  |  |  |  |  |  |  |  |  |  |  |  |  |  |  |  |  |  |  |  |  |  |  |  |  |  | 1.000 |  | .144 | -.014 | .045 |  |  |  | .085 | -.056 | -.004 |  | -.006 |  | .127 | .403 | -.032 | -.180 |  |  | .156 | .034 | -.089 |
|  |  | lastpregnw | -.496 |  |  |  |  |  |  |  |  |  |  |  |  |  |  |  |  |  |  |  |  |  |  |  |  |  |  |  |  |  |  | .144 |  | 1.000 | -.065 | .101 |  |  |  | .078 | .034 | .070 |  | -.011 |  | .007 | .053 | -.108 | -.036 |  |  | .035 | .068 | .063 |
|  |  | toldbpcrp | -.409 |  |  |  |  |  |  |  |  |  |  |  |  |  |  |  |  |  |  |  |  |  |  |  |  |  |  |  |  |  |  | -.014 |  | -.065 | 1.000 | -.122 |  |  |  | -.124 | .094 | .083 |  | -.027 |  | -.068 | -.087 | .084 | .065 |  |  | -.033 | .021 | -.043 |
|  |  | partneracc | -.202 |  |  |  |  |  |  |  |  |  |  |  |  |  |  |  |  |  |  |  |  |  |  |  |  |  |  |  |  |  |  | .045 |  | .101 | -.122 | 1.000 |  |  |  | .089 | .135 | .112 |  | -.082 |  | .064 | -.059 | -.134 | -.043 |  |  | .048 | -.016 | .029 |
|  |  | informed2r | -.291 |  |  |  |  |  |  |  |  |  |  |  |  |  |  |  |  |  |  |  |  |  |  |  |  |  |  |  |  |  |  | .085 |  | .078 | -.124 | .089 |  |  |  | 1.000 | .011 | -.020 |  | -.073 |  | .114 | .009 | -.095 | -.050 |  |  | .108 | .011 | -.070 |
|  |  | Monthly_income(1) | -.324 |  |  |  |  |  |  |  |  |  |  |  |  |  |  |  |  |  |  |  |  |  |  |  |  |  |  |  |  |  |  | -.056 |  | .034 | .094 | .135 |  |  |  | .011 | 1.000 | .629 |  | -.038 |  | .055 | -.205 | -.064 | -.003 |  |  | -.016 | -.072 | .032 |
|  |  | Monthly_income(2) | -.376 |  |  |  |  |  |  |  |  |  |  |  |  |  |  |  |  |  |  |  |  |  |  |  |  |  |  |  |  |  |  | -.004 |  | .070 | .083 | .112 |  |  |  | -.020 | .629 | 1.000 |  | -.240 |  | -.028 | .016 | -.120 | -.120 |  |  | .203 | -.111 | .151 |
|  |  | FirstANCBooking | -.012 |  |  |  |  |  |  |  |  |  |  |  |  |  |  |  |  |  |  |  |  |  |  |  |  |  |  |  |  |  |  | -.006 |  | -.011 | -.027 | -.082 |  |  |  | -.073 | -.038 | -.240 |  | 1.000 |  | -.202 | -.216 | .094 | .098 |  |  | -.109 | -.064 | -.206 |
|  |  | ModeTranspoCat(1) | -.075 |  |  |  |  |  |  |  |  |  |  |  |  |  |  |  |  |  |  |  |  |  |  |  |  |  |  |  |  |  |  | .127 |  | .007 | -.068 | .064 |  |  |  | .114 | .055 | -.028 |  | -.202 |  | 1.000 | .218 | -.059 | -.019 |  |  | .011 | -.046 | .093 |
|  |  | ModeTranspoCat(2) | -.122 |  |  |  |  |  |  |  |  |  |  |  |  |  |  |  |  |  |  |  |  |  |  |  |  |  |  |  |  |  |  | .403 |  | .053 | -.087 | -.059 |  |  |  | .009 | -.205 | .016 |  | -.216 |  | .218 | 1.000 | -.036 | -.013 |  |  | .105 | .039 | .034 |
|  |  | EducWomenCat(1) | -.291 |  |  |  |  |  |  |  |  |  |  |  |  |  |  |  |  |  |  |  |  |  |  |  |  |  |  |  |  |  |  | -.032 |  | -.108 | .084 | -.134 |  |  |  | -.095 | -.064 | -.120 |  | .094 |  | -.059 | -.036 | 1.000 | .757 |  |  | .069 | -.010 | -.109 |
|  |  | EducWomenCat(2) | -.371 |  |  |  |  |  |  |  |  |  |  |  |  |  |  |  |  |  |  |  |  |  |  |  |  |  |  |  |  |  |  | -.180 |  | -.036 | .065 | -.043 |  |  |  | -.050 | -.003 | -.120 |  | .098 |  | -.019 | -.013 | .757 | 1.000 |  |  | .164 | .080 | -.050 |
|  |  | AttendPWCLast | -.295 |  |  |  |  |  |  |  |  |  |  |  |  |  |  |  |  |  |  |  |  |  |  |  |  |  |  |  |  |  |  | .156 |  | .035 | -.033 | .048 |  |  |  | .108 | -.016 | .203 |  | -.109 |  | .011 | .105 | .069 | .164 |  |  | 1.000 | -.242 | -.207 |
|  |  | Number_of_live_children(1) | -.131 |  |  |  |  |  |  |  |  |  |  |  |  |  |  |  |  |  |  |  |  |  |  |  |  |  |  |  |  |  |  | .034 |  | .068 | .021 | -.016 |  |  |  | .011 | -.072 | -.111 |  | -.064 |  | -.046 | .039 | -.010 | .080 |  |  | -.242 | 1.000 | .288 |
|  |  | Number_of_live_children(2) | -.019 |  |  |  |  |  |  |  |  |  |  |  |  |  |  |  |  |  |  |  |  |  |  |  |  |  |  |  |  |  |  | -.089 |  | .063 | -.043 | .029 |  |  |  | -.070 | .032 | .151 |  | -.206 |  | .093 | .034 | -.109 | -.050 |  |  | -.207 | .288 | 1.000 |
| Step 14 | | Constant | 1.000 |  |  |  |  |  |  |  |  |  |  |  |  |  |  |  |  |  |  |  |  |  |  |  |  |  |  |  |  |  |  | -.345 |  | -.505 | -.403 | -.158 |  |  |  | -.315 |  |  |  | -.114 |  | -.079 | -.158 | -.392 | -.459 |  |  | -.264 | -.173 | .059 |
|  |  | time2reach | -.345 |  |  |  |  |  |  |  |  |  |  |  |  |  |  |  |  |  |  |  |  |  |  |  |  |  |  |  |  |  |  | 1.000 |  | .129 | -.024 | .038 |  |  |  | .074 |  |  |  | .019 |  | .138 | .407 | -.008 | -.173 |  |  | .143 | .029 | -.109 |
|  |  | lastpregnw | -.505 |  |  |  |  |  |  |  |  |  |  |  |  |  |  |  |  |  |  |  |  |  |  |  |  |  |  |  |  |  |  | .129 |  | 1.000 | -.086 | .095 |  |  |  | .077 |  |  |  | .013 |  | .018 | .041 | -.084 | -.017 |  |  | -.001 | .066 | .050 |
|  |  | toldbpcrp | -.403 |  |  |  |  |  |  |  |  |  |  |  |  |  |  |  |  |  |  |  |  |  |  |  |  |  |  |  |  |  |  | -.024 |  | -.086 | 1.000 | -.152 |  |  |  | -.129 |  |  |  | -.008 |  | -.084 | -.089 | .111 | .078 |  |  | -.043 | .041 | -.050 |
|  |  | partneracc | -.158 |  |  |  |  |  |  |  |  |  |  |  |  |  |  |  |  |  |  |  |  |  |  |  |  |  |  |  |  |  |  | .038 |  | .095 | -.152 | 1.000 |  |  |  | .081 |  |  |  | -.063 |  | .058 | -.041 | -.103 | -.026 |  |  | .038 | -.021 | -.004 |
|  |  | informed2r | -.315 |  |  |  |  |  |  |  |  |  |  |  |  |  |  |  |  |  |  |  |  |  |  |  |  |  |  |  |  |  |  | .074 |  | .077 | -.129 | .081 |  |  |  | 1.000 |  |  |  | -.083 |  | .111 | .020 | -.081 | -.039 |  |  | .134 | -.023 | -.090 |
|  |  | FirstANCBooking | -.114 |  |  |  |  |  |  |  |  |  |  |  |  |  |  |  |  |  |  |  |  |  |  |  |  |  |  |  |  |  |  | .019 |  | .013 | -.008 | -.063 |  |  |  | -.083 |  |  |  | 1.000 |  | -.247 | -.186 | .049 | .053 |  |  | -.025 | -.082 | -.171 |
|  |  | ModeTranspoCat(1) | -.079 |  |  |  |  |  |  |  |  |  |  |  |  |  |  |  |  |  |  |  |  |  |  |  |  |  |  |  |  |  |  | .138 |  | .018 | -.084 | .058 |  |  |  | .111 |  |  |  | -.247 |  | 1.000 | .265 | -.072 | -.029 |  |  | .036 | -.043 | .103 |
|  |  | ModeTranspoCat(2) | -.158 |  |  |  |  |  |  |  |  |  |  |  |  |  |  |  |  |  |  |  |  |  |  |  |  |  |  |  |  |  |  | .407 |  | .041 | -.089 | -.041 |  |  |  | .020 |  |  |  | -.186 |  | .265 | 1.000 | -.029 | .014 |  |  | .067 | .028 | -.023 |
|  |  | EducWomenCat(1) | -.392 |  |  |  |  |  |  |  |  |  |  |  |  |  |  |  |  |  |  |  |  |  |  |  |  |  |  |  |  |  |  | -.008 |  | -.084 | .111 | -.103 |  |  |  | -.081 |  |  |  | .049 |  | -.072 | -.029 | 1.000 | .747 |  |  | .101 | -.020 | -.092 |
|  |  | EducWomenCat(2) | -.459 |  |  |  |  |  |  |  |  |  |  |  |  |  |  |  |  |  |  |  |  |  |  |  |  |  |  |  |  |  |  | -.173 |  | -.017 | .078 | -.026 |  |  |  | -.039 |  |  |  | .053 |  | -.029 | .014 | .747 | 1.000 |  |  | .220 | .062 | -.039 |
|  |  | AttendPWCLast | -.264 |  |  |  |  |  |  |  |  |  |  |  |  |  |  |  |  |  |  |  |  |  |  |  |  |  |  |  |  |  |  | .143 |  | -.001 | -.043 | .038 |  |  |  | .134 |  |  |  | -.025 |  | .036 | .067 | .101 | .220 |  |  | 1.000 | -.240 | -.282 |
|  |  | Number_of_live_children(1) | -.173 |  |  |  |  |  |  |  |  |  |  |  |  |  |  |  |  |  |  |  |  |  |  |  |  |  |  |  |  |  |  | .029 |  | .066 | .041 | -.021 |  |  |  | -.023 |  |  |  | -.082 |  | -.043 | .028 | -.020 | .062 |  |  | -.240 | 1.000 | .307 |
|  |  | Number_of_live_children(2) | .059 |  |  |  |  |  |  |  |  |  |  |  |  |  |  |  |  |  |  |  |  |  |  |  |  |  |  |  |  |  |  | -.109 |  | .050 | -.050 | -.004 |  |  |  | -.090 |  |  |  | -.171 |  | .103 | -.023 | -.092 | -.039 |  |  | -.282 | .307 | 1.000 |
| Step 15 | | Constant | 1.000 |  |  |  |  |  |  |  |  |  |  |  |  |  |  |  |  |  |  |  |  |  |  |  |  |  |  |  |  |  |  | -.391 |  | -.599 |  | -.249 |  |  |  | -.392 |  |  |  | -.162 |  | -.120 | -.208 | -.380 | -.463 |  |  | -.300 | -.159 | .043 |
|  |  | time2reach | -.391 |  |  |  |  |  |  |  |  |  |  |  |  |  |  |  |  |  |  |  |  |  |  |  |  |  |  |  |  |  |  | 1.000 |  | .142 |  | .024 |  |  |  | .062 |  |  |  | .057 |  | .136 | .392 | -.004 | -.166 |  |  | .161 | .001 | -.126 |
|  |  | lastpregnw | -.599 |  |  |  |  |  |  |  |  |  |  |  |  |  |  |  |  |  |  |  |  |  |  |  |  |  |  |  |  |  |  | .142 |  | 1.000 |  | .102 |  |  |  | .054 |  |  |  | .034 |  | .006 | .030 | -.069 | -.026 |  |  | -.015 | .069 | .044 |
|  |  | partneracc | -.249 |  |  |  |  |  |  |  |  |  |  |  |  |  |  |  |  |  |  |  |  |  |  |  |  |  |  |  |  |  |  | .024 |  | .102 |  | 1.000 |  |  |  | .055 |  |  |  | -.063 |  | .050 | -.055 | -.086 | .004 |  |  | .043 | -.028 | -.020 |
|  |  | informed2r | -.392 |  |  |  |  |  |  |  |  |  |  |  |  |  |  |  |  |  |  |  |  |  |  |  |  |  |  |  |  |  |  | .062 |  | .054 |  | .055 |  |  |  | 1.000 |  |  |  | -.060 |  | .117 | .009 | -.082 | -.032 |  |  | .125 | -.025 | -.091 |
|  |  | FirstANCBooking | -.162 |  |  |  |  |  |  |  |  |  |  |  |  |  |  |  |  |  |  |  |  |  |  |  |  |  |  |  |  |  |  | .057 |  | .034 |  | -.063 |  |  |  | -.060 |  |  |  | 1.000 |  | -.244 | -.163 | .056 | .046 |  |  | -.010 | -.081 | -.185 |
|  |  | ModeTranspoCat(1) | -.120 |  |  |  |  |  |  |  |  |  |  |  |  |  |  |  |  |  |  |  |  |  |  |  |  |  |  |  |  |  |  | .136 |  | .006 |  | .050 |  |  |  | .117 |  |  |  | -.244 |  | 1.000 | .253 | -.063 | -.029 |  |  | .026 | -.055 | .096 |
|  |  | ModeTranspoCat(2) | -.208 |  |  |  |  |  |  |  |  |  |  |  |  |  |  |  |  |  |  |  |  |  |  |  |  |  |  |  |  |  |  | .392 |  | .030 |  | -.055 |  |  |  | .009 |  |  |  | -.163 |  | .253 | 1.000 | -.018 | .027 |  |  | .067 | .019 | -.033 |
|  |  | EducWomenCat(1) | -.380 |  |  |  |  |  |  |  |  |  |  |  |  |  |  |  |  |  |  |  |  |  |  |  |  |  |  |  |  |  |  | -.004 |  | -.069 |  | -.086 |  |  |  | -.082 |  |  |  | .056 |  | -.063 | -.018 | 1.000 | .749 |  |  | .094 | -.026 | -.078 |
|  |  | EducWomenCat(2) | -.463 |  |  |  |  |  |  |  |  |  |  |  |  |  |  |  |  |  |  |  |  |  |  |  |  |  |  |  |  |  |  | -.166 |  | -.026 |  | .004 |  |  |  | -.032 |  |  |  | .046 |  | -.029 | .027 | .749 | 1.000 |  |  | .210 | .060 | -.025 |
|  |  | AttendPWCLast | -.300 |  |  |  |  |  |  |  |  |  |  |  |  |  |  |  |  |  |  |  |  |  |  |  |  |  |  |  |  |  |  | .161 |  | -.015 |  | .043 |  |  |  | .125 |  |  |  | -.010 |  | .026 | .067 | .094 | .210 |  |  | 1.000 | -.233 | -.279 |
|  |  | Number_of_live_children(1) | -.159 |  |  |  |  |  |  |  |  |  |  |  |  |  |  |  |  |  |  |  |  |  |  |  |  |  |  |  |  |  |  | .001 |  | .069 |  | -.028 |  |  |  | -.025 |  |  |  | -.081 |  | -.055 | .019 | -.026 | .060 |  |  | -.233 | 1.000 | .309 |
|  |  | Number_of_live_children(2) | .043 |  |  |  |  |  |  |  |  |  |  |  |  |  |  |  |  |  |  |  |  |  |  |  |  |  |  |  |  |  |  | -.126 |  | .044 |  | -.020 |  |  |  | -.091 |  |  |  | -.185 |  | .096 | -.033 | -.078 | -.025 |  |  | -.279 | .309 | 1.000 |

| Model if Term Removed | | | | | |
| --- | --- | --- | --- | --- | --- |
| Variable | | Model Log Likelihood | Change in -2 Log Likelihood | df | Sig. of the Change |
| Step 1 | residence | -94.830 | 2.099 | 1 | .147 |
|  | time2reach | -97.960 | 8.360 | 1 | .004 |
|  | when2begin | -94.080 | .599 | 1 | .439 |
|  | culturevsp | -93.784 | .008 | 1 | .931 |
|  | lastpregnw | -95.943 | 4.325 | 1 | .038 |
|  | toldpregnc | -93.965 | .369 | 1 | .543 |
|  | toldbpcrp | -94.661 | 1.763 | 1 | .184 |
|  | partneracc | -99.756 | 11.951 | 1 | .001 |
|  | placedeliv | -95.374 | 3.188 | 2 | .203 |
|  | be4dischar | -94.093 | .626 | 1 | .429 |
|  | informed2r | -95.608 | 3.657 | 1 | .056 |
|  | Monthly_income | -95.275 | 2.990 | 2 | .224 |
|  | AgePregCat | -94.008 | .456 | 1 | .500 |
|  | FirstANCBooking | -97.082 | 6.604 | 1 | .010 |
|  | BPCRPKnowledge | -94.001 | .442 | 1 | .506 |
|  | ModeTranspoCat | -95.547 | 3.534 | 2 | .171 |
|  | EducWomenCat | -95.424 | 3.288 | 2 | .193 |
|  | EducHusbCat2 | -95.123 | 2.686 | 2 | .261 |
|  | AttendPWCLast | -110.753 | 33.946 | 1 | .000 |
|  | OccupWomLast | -93.954 | .347 | 1 | .556 |
|  | OccupHusbcatt | -93.913 | .266 | 1 | .606 |
|  | ReceivedadequateANCServices | -93.994 | .428 | 1 | .513 |
|  | Number_of_live_children | -95.763 | 3.965 | 2 | .138 |
| Step 2 | residence | -94.833 | 2.098 | 1 | .148 |
|  | time2reach | -97.969 | 8.370 | 1 | .004 |
|  | when2begin | -94.096 | .625 | 1 | .429 |
|  | lastpregnw | -96.007 | 4.447 | 1 | .035 |
|  | toldpregnc | -93.968 | .368 | 1 | .544 |
|  | toldbpcrp | -94.666 | 1.764 | 1 | .184 |
|  | partneracc | -100.189 | 12.811 | 1 | .000 |
|  | placedeliv | -95.386 | 3.204 | 2 | .201 |
|  | be4dischar | -94.119 | .670 | 1 | .413 |
|  | informed2r | -95.615 | 3.661 | 1 | .056 |
|  | Monthly_income | -95.284 | 3.001 | 2 | .223 |
|  | AgePregCat | -94.017 | .466 | 1 | .495 |
|  | FirstANCBooking | -97.114 | 6.660 | 1 | .010 |
|  | BPCRPKnowledge | -94.007 | .446 | 1 | .504 |
|  | ModeTranspoCat | -95.599 | 3.631 | 2 | .163 |
|  | EducWomenCat | -95.424 | 3.281 | 2 | .194 |
|  | EducHusbCat2 | -95.143 | 2.718 | 2 | .257 |
|  | AttendPWCLast | -111.378 | 35.189 | 1 | .000 |
|  | OccupWomLast | -93.956 | .344 | 1 | .558 |
|  | OccupHusbcatt | -93.916 | .264 | 1 | .607 |
|  | ReceivedadequateANCServices | -93.996 | .425 | 1 | .514 |
|  | Number_of_live_children | -95.770 | 3.973 | 2 | .137 |
| Step 3 | residence | -94.861 | 1.889 | 1 | .169 |
|  | time2reach | -98.422 | 9.012 | 1 | .003 |
|  | when2begin | -94.243 | .653 | 1 | .419 |
|  | lastpregnw | -96.155 | 4.478 | 1 | .034 |
|  | toldpregnc | -94.097 | .361 | 1 | .548 |
|  | toldbpcrp | -94.766 | 1.700 | 1 | .192 |
|  | partneracc | -100.549 | 13.266 | 1 | .000 |
|  | placedeliv | -95.527 | 3.223 | 2 | .200 |
|  | be4dischar | -94.210 | .588 | 1 | .443 |
|  | informed2r | -95.807 | 3.782 | 1 | .052 |
|  | Monthly_income | -95.525 | 3.218 | 2 | .200 |
|  | AgePregCat | -94.152 | .472 | 1 | .492 |
|  | FirstANCBooking | -97.479 | 7.125 | 1 | .008 |
|  | BPCRPKnowledge | -94.130 | .427 | 1 | .513 |
|  | ModeTranspoCat | -95.902 | 3.971 | 2 | .137 |
|  | EducWomenCat | -95.451 | 3.069 | 2 | .216 |
|  | EducHusbCat2 | -95.747 | 3.661 | 2 | .160 |
|  | AttendPWCLast | -111.574 | 35.316 | 1 | .000 |
|  | OccupWomLast | -94.010 | .188 | 1 | .665 |
|  | ReceivedadequateANCServices | -94.115 | .398 | 1 | .528 |
|  | Number_of_live_children | -96.005 | 4.178 | 2 | .124 |
| Step 4 | residence | -95.150 | 2.279 | 1 | .131 |
|  | time2reach | -98.593 | 9.167 | 1 | .002 |
|  | when2begin | -94.327 | .633 | 1 | .426 |
|  | lastpregnw | -96.225 | 4.429 | 1 | .035 |
|  | toldpregnc | -94.170 | .321 | 1 | .571 |
|  | toldbpcrp | -94.887 | 1.754 | 1 | .185 |
|  | partneracc | -100.568 | 13.116 | 1 | .000 |
|  | placedeliv | -95.555 | 3.091 | 2 | .213 |
|  | be4dischar | -94.340 | .660 | 1 | .416 |
|  | informed2r | -95.941 | 3.862 | 1 | .049 |
|  | Monthly_income | -95.550 | 3.080 | 2 | .214 |
|  | AgePregCat | -94.183 | .345 | 1 | .557 |
|  | FirstANCBooking | -97.525 | 7.030 | 1 | .008 |
|  | BPCRPKnowledge | -94.211 | .401 | 1 | .526 |
|  | ModeTranspoCat | -95.948 | 3.876 | 2 | .144 |
|  | EducWomenCat | -95.451 | 2.882 | 2 | .237 |
|  | EducHusbCat2 | -95.784 | 3.549 | 2 | .170 |
|  | AttendPWCLast | -111.574 | 35.129 | 1 | .000 |
|  | ReceivedadequateANCServices | -94.181 | .342 | 1 | .559 |
|  | Number_of_live_children | -96.283 | 4.547 | 2 | .103 |
| Step 5 | residence | -95.268 | 2.196 | 1 | .138 |
|  | time2reach | -98.654 | 8.967 | 1 | .003 |
|  | when2begin | -94.443 | .544 | 1 | .461 |
|  | lastpregnw | -96.354 | 4.366 | 1 | .037 |
|  | toldbpcrp | -95.740 | 3.139 | 1 | .076 |
|  | partneracc | -100.571 | 12.801 | 1 | .000 |
|  | placedeliv | -95.614 | 2.886 | 2 | .236 |
|  | be4dischar | -94.505 | .669 | 1 | .413 |
|  | informed2r | -96.171 | 4.002 | 1 | .045 |
|  | Monthly_income | -95.662 | 2.984 | 2 | .225 |
|  | AgePregCat | -94.345 | .349 | 1 | .555 |
|  | FirstANCBooking | -97.536 | 6.731 | 1 | .009 |
|  | BPCRPKnowledge | -94.336 | .330 | 1 | .565 |
|  | ModeTranspoCat | -96.293 | 4.244 | 2 | .120 |
|  | EducWomenCat | -95.603 | 2.865 | 2 | .239 |
|  | EducHusbCat2 | -95.878 | 3.416 | 2 | .181 |
|  | AttendPWCLast | -111.828 | 35.316 | 1 | .000 |
|  | ReceivedadequateANCServices | -94.299 | .257 | 1 | .612 |
|  | Number_of_live_children | -96.415 | 4.490 | 2 | .106 |
| Step 6 | residence | -95.372 | 2.145 | 1 | .143 |
|  | time2reach | -98.808 | 9.019 | 1 | .003 |
|  | when2begin | -94.514 | .430 | 1 | .512 |
|  | lastpregnw | -96.473 | 4.348 | 1 | .037 |
|  | toldbpcrp | -95.741 | 2.884 | 1 | .089 |
|  | partneracc | -100.584 | 12.570 | 1 | .000 |
|  | placedeliv | -95.621 | 2.643 | 2 | .267 |
|  | be4dischar | -94.569 | .539 | 1 | .463 |
|  | informed2r | -96.304 | 4.010 | 1 | .045 |
|  | Monthly_income | -95.803 | 3.009 | 2 | .222 |
|  | AgePregCat | -94.494 | .390 | 1 | .532 |
|  | FirstANCBooking | -97.581 | 6.564 | 1 | .010 |
|  | BPCRPKnowledge | -94.535 | .471 | 1 | .493 |
|  | ModeTranspoCat | -96.534 | 4.470 | 2 | .107 |
|  | EducWomenCat | -95.681 | 2.763 | 2 | .251 |
|  | EducHusbCat2 | -95.911 | 3.224 | 2 | .199 |
|  | AttendPWCLast | -111.872 | 35.147 | 1 | .000 |
|  | Number_of_live_children | -96.494 | 4.391 | 2 | .111 |
| Step 7 | residence | -95.646 | 2.302 | 1 | .129 |
|  | time2reach | -99.112 | 9.235 | 1 | .002 |
|  | when2begin | -94.722 | .456 | 1 | .500 |
|  | lastpregnw | -96.888 | 4.788 | 1 | .029 |
|  | toldbpcrp | -95.865 | 2.741 | 1 | .098 |
|  | partneracc | -100.635 | 12.281 | 1 | .000 |
|  | placedeliv | -95.712 | 2.436 | 2 | .296 |
|  | be4dischar | -94.754 | .518 | 1 | .472 |
|  | informed2r | -96.491 | 3.993 | 1 | .046 |
|  | Monthly_income | -96.136 | 3.283 | 2 | .194 |
|  | FirstANCBooking | -97.622 | 6.255 | 1 | .012 |
|  | BPCRPKnowledge | -94.725 | .462 | 1 | .497 |
|  | ModeTranspoCat | -96.683 | 4.377 | 2 | .112 |
|  | EducWomenCat | -96.054 | 3.119 | 2 | .210 |
|  | EducHusbCat2 | -95.992 | 2.995 | 2 | .224 |
|  | AttendPWCLast | -113.274 | 37.559 | 1 | .000 |
|  | Number_of_live_children | -96.601 | 4.213 | 2 | .122 |
| Step 8 | residence | -95.777 | 2.110 | 1 | .146 |
|  | time2reach | -99.269 | 9.093 | 1 | .003 |
|  | lastpregnw | -97.054 | 4.663 | 1 | .031 |
|  | toldbpcrp | -96.063 | 2.683 | 1 | .101 |
|  | partneracc | -100.677 | 11.909 | 1 | .001 |
|  | placedeliv | -95.810 | 2.175 | 2 | .337 |
|  | be4dischar | -94.959 | .474 | 1 | .491 |
|  | informed2r | -96.854 | 4.263 | 1 | .039 |
|  | Monthly_income | -96.560 | 3.676 | 2 | .159 |
|  | FirstANCBooking | -97.757 | 6.069 | 1 | .014 |
|  | BPCRPKnowledge | -95.014 | .584 | 1 | .445 |
|  | ModeTranspoCat | -96.854 | 4.263 | 2 | .119 |
|  | EducWomenCat | -96.274 | 3.104 | 2 | .212 |
|  | EducHusbCat2 | -96.133 | 2.822 | 2 | .244 |
|  | AttendPWCLast | -113.526 | 37.608 | 1 | .000 |
|  | Number_of_live_children | -96.838 | 4.231 | 2 | .121 |
| Step 9 | residence | -96.021 | 2.123 | 1 | .145 |
|  | time2reach | -100.147 | 10.374 | 1 | .001 |
|  | lastpregnw | -97.382 | 4.844 | 1 | .028 |
|  | toldbpcrp | -96.337 | 2.756 | 1 | .097 |
|  | partneracc | -100.760 | 11.602 | 1 | .001 |
|  | placedeliv | -95.924 | 1.929 | 2 | .381 |
|  | informed2r | -97.838 | 5.756 | 1 | .016 |
|  | Monthly_income | -96.663 | 3.407 | 2 | .182 |
|  | FirstANCBooking | -97.819 | 5.719 | 1 | .017 |
|  | BPCRPKnowledge | -95.194 | .468 | 1 | .494 |
|  | ModeTranspoCat | -97.342 | 4.765 | 2 | .092 |
|  | EducWomenCat | -96.354 | 2.788 | 2 | .248 |
|  | EducHusbCat2 | -96.368 | 2.818 | 2 | .244 |
|  | AttendPWCLast | -117.110 | 44.301 | 1 | .000 |
|  | Number_of_live_children | -97.178 | 4.437 | 2 | .109 |
| Step 10 | residence | -96.417 | 2.446 | 1 | .118 |
|  | time2reach | -100.409 | 10.430 | 1 | .001 |
|  | lastpregnw | -97.522 | 4.657 | 1 | .031 |
|  | toldbpcrp | -96.395 | 2.403 | 1 | .121 |
|  | partneracc | -100.760 | 11.134 | 1 | .001 |
|  | placedeliv | -96.151 | 1.915 | 2 | .384 |
|  | informed2r | -98.064 | 5.741 | 1 | .017 |
|  | Monthly_income | -96.811 | 3.235 | 2 | .198 |
|  | FirstANCBooking | -98.044 | 5.701 | 1 | .017 |
|  | ModeTranspoCat | -97.662 | 4.937 | 2 | .085 |
|  | EducWomenCat | -96.555 | 2.722 | 2 | .256 |
|  | EducHusbCat2 | -96.517 | 2.647 | 2 | .266 |
|  | AttendPWCLast | -117.139 | 43.891 | 1 | .000 |
|  | Number_of_live_children | -97.423 | 4.458 | 2 | .108 |
| Step 11 | residence | -97.077 | 1.851 | 1 | .174 |
|  | time2reach | -101.345 | 10.388 | 1 | .001 |
|  | lastpregnw | -98.258 | 4.215 | 1 | .040 |
|  | toldbpcrp | -97.180 | 2.058 | 1 | .151 |
|  | partneracc | -102.104 | 11.905 | 1 | .001 |
|  | informed2r | -99.152 | 6.003 | 1 | .014 |
|  | Monthly_income | -97.648 | 2.995 | 2 | .224 |
|  | FirstANCBooking | -99.004 | 5.705 | 1 | .017 |
|  | ModeTranspoCat | -98.942 | 5.581 | 2 | .061 |
|  | EducWomenCat | -97.775 | 3.249 | 2 | .197 |
|  | EducHusbCat2 | -97.556 | 2.809 | 2 | .245 |
|  | AttendPWCLast | -118.263 | 44.225 | 1 | .000 |
|  | Number_of_live_children | -98.275 | 4.248 | 2 | .120 |
| Step 12 | residence | -98.286 | 1.460 | 1 | .227 |
|  | time2reach | -103.067 | 11.023 | 1 | .001 |
|  | lastpregnw | -99.260 | 3.409 | 1 | .065 |
|  | toldbpcrp | -98.771 | 2.430 | 1 | .119 |
|  | partneracc | -102.929 | 10.748 | 1 | .001 |
|  | informed2r | -100.279 | 5.447 | 1 | .020 |
|  | Monthly_income | -99.530 | 3.948 | 2 | .139 |
|  | FirstANCBooking | -100.653 | 6.195 | 1 | .013 |
|  | ModeTranspoCat | -100.365 | 5.618 | 2 | .060 |
|  | EducWomenCat | -100.791 | 6.471 | 2 | .039 |
|  | AttendPWCLast | -120.109 | 45.107 | 1 | .000 |
|  | Number_of_live_children | -99.965 | 4.820 | 2 | .090 |
| Step 13 | time2reach | -103.067 | 9.563 | 1 | .002 |
|  | lastpregnw | -100.309 | 4.047 | 1 | .044 |
|  | toldbpcrp | -99.731 | 2.891 | 1 | .089 |
|  | partneracc | -103.271 | 9.971 | 1 | .002 |
|  | informed2r | -101.360 | 6.148 | 1 | .013 |
|  | Monthly_income | -99.685 | 2.798 | 2 | .247 |
|  | FirstANCBooking | -101.107 | 5.643 | 1 | .018 |
|  | ModeTranspoCat | -101.230 | 5.888 | 2 | .053 |
|  | EducWomenCat | -101.145 | 5.719 | 2 | .057 |
|  | AttendPWCLast | -122.162 | 47.753 | 1 | .000 |
|  | Number_of_live_children | -100.525 | 4.480 | 2 | .106 |
| Step 14 | time2reach | -104.623 | 9.876 | 1 | .002 |
|  | lastpregnw | -101.618 | 3.866 | 1 | .049 |
|  | toldbpcrp | -101.018 | 2.667 | 1 | .102 |
|  | partneracc | -104.612 | 9.855 | 1 | .002 |
|  | informed2r | -103.154 | 6.938 | 1 | .008 |
|  | FirstANCBooking | -104.215 | 9.061 | 1 | .003 |
|  | ModeTranspoCat | -102.399 | 5.428 | 2 | .066 |
|  | EducWomenCat | -103.469 | 7.569 | 2 | .023 |
|  | AttendPWCLast | -122.360 | 45.350 | 1 | .000 |
|  | Number_of_live_children | -102.385 | 5.400 | 2 | .067 |
| Step 15 | time2reach | -106.391 | 10.746 | 1 | .001 |
|  | lastpregnw | -103.296 | 4.556 | 1 | .033 |
|  | partneracc | -107.315 | 12.594 | 1 | .000 |
|  | informed2r | -105.444 | 8.851 | 1 | .003 |
|  | FirstANCBooking | -105.816 | 9.596 | 1 | .002 |
|  | ModeTranspoCat | -104.088 | 6.139 | 2 | .046 |
|  | EducWomenCat | -104.722 | 7.408 | 2 | .025 |
|  | AttendPWCLast | -125.831 | 49.625 | 1 | .000 |
|  | Number_of_live_children | -103.713 | 5.390 | 2 | .068 |

| Variables not in the Equation | | | | | |
| --- | --- | --- | --- | --- | --- |
|  | | | Score | df | Sig. |
| Step 2^a^ | Variables | culturevsp | .008 | 1 | .931 |
|  | Overall Statistics | | .008 | 1 | .931 |
| Step 3^b^ | Variables | culturevsp | .006 | 1 | .937 |
|  |  | OccupHusbcatt | .266 | 1 | .606 |
|  | Overall Statistics | | .273 | 2 | .872 |
| Step 4^c^ | Variables | culturevsp | .004 | 1 | .947 |
|  |  | OccupWomLast | .186 | 1 | .666 |
|  |  | OccupHusbcatt | .108 | 1 | .742 |
|  | Overall Statistics | | .460 | 3 | .928 |
| Step 5^d^ | Variables | culturevsp | .003 | 1 | .953 |
|  |  | toldpregnc | .320 | 1 | .572 |
|  |  | OccupWomLast | .146 | 1 | .702 |
|  |  | OccupHusbcatt | .114 | 1 | .735 |
|  | Overall Statistics | | .781 | 4 | .941 |
| Step 6^e^ | Variables | culturevsp | .003 | 1 | .958 |
|  |  | toldpregnc | .236 | 1 | .627 |
|  |  | OccupWomLast | .105 | 1 | .746 |
|  |  | OccupHusbcatt | .114 | 1 | .736 |
|  |  | ReceivedadequateANCServices | .262 | 1 | .609 |
|  | Overall Statistics | | 1.048 | 5 | .959 |
| Step 7^f^ | Variables | culturevsp | .010 | 1 | .919 |
|  |  | toldpregnc | .230 | 1 | .631 |
|  |  | AgePregCat(1) | .387 | 1 | .534 |
|  |  | OccupWomLast | .017 | 1 | .897 |
|  |  | OccupHusbcatt | .163 | 1 | .686 |
|  |  | ReceivedadequateANCServices | .305 | 1 | .581 |
|  | Overall Statistics | | 1.441 | 6 | .963 |
| Step 8^g^ | Variables | when2begin | .454 | 1 | .500 |
|  |  | culturevsp | .036 | 1 | .850 |
|  |  | toldpregnc | .178 | 1 | .673 |
|  |  | AgePregCat(1) | .412 | 1 | .521 |
|  |  | OccupWomLast | .016 | 1 | .900 |
|  |  | OccupHusbcatt | .181 | 1 | .670 |
|  |  | ReceivedadequateANCServices | .172 | 1 | .678 |
|  | Overall Statistics | | 1.908 | 7 | .965 |
| Step 9^h^ | Variables | when2begin | .410 | 1 | .522 |
|  |  | culturevsp | .085 | 1 | .771 |
|  |  | toldpregnc | .198 | 1 | .656 |
|  |  | be4dischar | .467 | 1 | .494 |
|  |  | AgePregCat(1) | .391 | 1 | .532 |
|  |  | OccupWomLast | .040 | 1 | .841 |
|  |  | OccupHusbcatt | .100 | 1 | .752 |
|  |  | ReceivedadequateANCServices | .075 | 1 | .784 |
|  | Overall Statistics | | 2.394 | 8 | .966 |
| Step 10^i^ | Variables | when2begin | .514 | 1 | .473 |
|  |  | culturevsp | .097 | 1 | .755 |
|  |  | toldpregnc | .110 | 1 | .740 |
|  |  | be4dischar | .355 | 1 | .551 |
|  |  | AgePregCat(1) | .388 | 1 | .533 |
|  |  | BPCRPKnowledge | .471 | 1 | .492 |
|  |  | OccupWomLast | .025 | 1 | .875 |
|  |  | OccupHusbcatt | .096 | 1 | .756 |
|  |  | ReceivedadequateANCServices | .166 | 1 | .683 |
|  | Overall Statistics | | 2.880 | 9 | .969 |
| Step 11^j^ | Variables | when2begin | .281 | 1 | .596 |
|  |  | culturevsp | .085 | 1 | .770 |
|  |  | toldpregnc | .051 | 1 | .821 |
|  |  | placedeliv | 1.486 | 2 | .476 |
|  |  | placedeliv(1) | .920 | 1 | .338 |
|  |  | placedeliv(2) | .594 | 1 | .441 |
|  |  | be4dischar | .162 | 1 | .687 |
|  |  | AgePregCat(1) | .202 | 1 | .653 |
|  |  | BPCRPKnowledge | .457 | 1 | .499 |
|  |  | OccupWomLast | .009 | 1 | .925 |
|  |  | OccupHusbcatt | .132 | 1 | .717 |
|  |  | ReceivedadequateANCServices | .016 | 1 | .900 |
|  | Overall Statistics | | 4.323 | 11 | .960 |
| Step 12^k^ | Variables | when2begin | .133 | 1 | .715 |
|  |  | culturevsp | .145 | 1 | .703 |
|  |  | toldpregnc | .030 | 1 | .863 |
|  |  | placedeliv | 1.623 | 2 | .444 |
|  |  | placedeliv(1) | 1.050 | 1 | .306 |
|  |  | placedeliv(2) | .683 | 1 | .409 |
|  |  | be4dischar | .174 | 1 | .676 |
|  |  | AgePregCat(1) | .046 | 1 | .830 |
|  |  | BPCRPKnowledge | .265 | 1 | .607 |
|  |  | EducHusbCat2 | 2.622 | 2 | .270 |
|  |  | EducHusbCat2(1) | .130 | 1 | .718 |
|  |  | EducHusbCat2(2) | 1.799 | 1 | .180 |
|  |  | OccupWomLast | .001 | 1 | .971 |
|  |  | OccupHusbcatt | .773 | 1 | .379 |
|  |  | ReceivedadequateANCServices | .014 | 1 | .907 |
|  | Overall Statistics | | 6.953 | 13 | .905 |
| Step 13^l^ | Variables | residence | 1.456 | 1 | .228 |
|  |  | when2begin | .100 | 1 | .752 |
|  |  | culturevsp | .117 | 1 | .732 |
|  |  | toldpregnc | .022 | 1 | .883 |
|  |  | placedeliv | 1.005 | 2 | .605 |
|  |  | placedeliv(1) | .408 | 1 | .523 |
|  |  | placedeliv(2) | .211 | 1 | .646 |
|  |  | be4dischar | .241 | 1 | .624 |
|  |  | AgePregCat(1) | .135 | 1 | .713 |
|  |  | BPCRPKnowledge | .507 | 1 | .476 |
|  |  | EducHusbCat2 | 2.290 | 2 | .318 |
|  |  | EducHusbCat2(1) | .114 | 1 | .736 |
|  |  | EducHusbCat2(2) | 1.576 | 1 | .209 |
|  |  | OccupWomLast | .089 | 1 | .765 |
|  |  | OccupHusbcatt | .257 | 1 | .612 |
|  |  | ReceivedadequateANCServices | .000 | 1 | .983 |
|  | Overall Statistics | | 8.155 | 14 | .881 |
| Step 14^m^ | Variables | residence | .311 | 1 | .577 |
|  |  | when2begin | .310 | 1 | .578 |
|  |  | culturevsp | .037 | 1 | .847 |
|  |  | toldpregnc | .003 | 1 | .954 |
|  |  | placedeliv | 1.189 | 2 | .552 |
|  |  | placedeliv(1) | .754 | 1 | .385 |
|  |  | placedeliv(2) | .518 | 1 | .472 |
|  |  | be4dischar | .074 | 1 | .786 |
|  |  | Monthly_income | 2.737 | 2 | .255 |
|  |  | Monthly_income(1) | .526 | 1 | .468 |
|  |  | Monthly_income(2) | 2.616 | 1 | .106 |
|  |  | AgePregCat(1) | .217 | 1 | .642 |
|  |  | BPCRPKnowledge | .225 | 1 | .635 |
|  |  | EducHusbCat2 | 3.189 | 2 | .203 |
|  |  | EducHusbCat2(1) | .122 | 1 | .727 |
|  |  | EducHusbCat2(2) | 2.175 | 1 | .140 |
|  |  | OccupWomLast | .016 | 1 | .899 |
|  |  | OccupHusbcatt | .968 | 1 | .325 |
|  |  | ReceivedadequateANCServices | .003 | 1 | .956 |
|  | Overall Statistics | | 10.497 | 16 | .839 |
| Step 15^n^ | Variables | residence | .560 | 1 | .454 |
|  |  | when2begin | .179 | 1 | .673 |
|  |  | culturevsp | .034 | 1 | .854 |
|  |  | toldpregnc | .711 | 1 | .399 |
|  |  | toldbpcrp | 2.466 | 1 | .116 |
|  |  | placedeliv | .823 | 2 | .663 |
|  |  | placedeliv(1) | .382 | 1 | .537 |
|  |  | placedeliv(2) | .224 | 1 | .636 |
|  |  | be4dischar | .161 | 1 | .689 |
|  |  | Monthly_income | 2.521 | 2 | .284 |
|  |  | Monthly_income(1) | .706 | 1 | .401 |
|  |  | Monthly_income(2) | 2.489 | 1 | .115 |
|  |  | AgePregCat(1) | .132 | 1 | .717 |
|  |  | BPCRPKnowledge | .030 | 1 | .862 |
|  |  | EducHusbCat2 | 3.370 | 2 | .185 |
|  |  | EducHusbCat2(1) | .173 | 1 | .678 |
|  |  | EducHusbCat2(2) | 2.396 | 1 | .122 |
|  |  | OccupWomLast | .003 | 1 | .959 |
|  |  | OccupHusbcatt | .713 | 1 | .399 |
|  |  | ReceivedadequateANCServices | .130 | 1 | .719 |
|  | Overall Statistics | | 12.847 | 17 | .746 |
| a. Variable(s) removed on step 2: culturevsp. | | | | | |
| b. Variable(s) removed on step 3: OccupHusbcatt. | | | | | |
| c. Variable(s) removed on step 4: OccupWomLast. | | | | | |
| d. Variable(s) removed on step 5: toldpregnc. | | | | | |
| e. Variable(s) removed on step 6: ReceivedadequateANCServices. | | | | | |
| f. Variable(s) removed on step 7: AgePregCat. | | | | | |
| g. Variable(s) removed on step 8: when2begin. | | | | | |
| h. Variable(s) removed on step 9: be4dischar. | | | | | |
| i. Variable(s) removed on step 10: BPCRPKnowledge. | | | | | |
| j. Variable(s) removed on step 11: placedeliv. | | | | | |
| k. Variable(s) removed on step 12: EducHusbCat2. | | | | | |
| l. Variable(s) removed on step 13: residence. | | | | | |
| m. Variable(s) removed on step 14: Monthly_income. | | | | | |
| n. Variable(s) removed on step 15: toldbpcrp. | | | | | |
